# Cytochalasans Act as Inhibitors of Biofilm Formation of *Staphylococcus aureus*

**DOI:** 10.3390/biom8040129

**Published:** 2018-10-30

**Authors:** Kamila Tomoko Yuyama, Lucile Wendt, Frank Surup, Robin Kretz, Clara Chepkirui, Kathrin Wittstein, Chollaratt Boonlarppradab, Sarunyou Wongkanoun, Jennifer Luangsa-ard, Marc Stadler, Wolf-Rainer Abraham

**Affiliations:** 1 Department Chemical Microbiology, Helmholtz Centre for Infection Research (HZI), Inhoffenstraße 7, 38124 Braunschweig, Germany; kamilatomoko@gmail.com; 2 Department Microbial Drugs, Helmholtz Centre for Infection Research (HZI), Inhoffenstraße 7, 38124 Braunschweig, Germany; lucile.wendt@helmholtz-hzi.de (L.W.); Frank.Surup@helmholtz-hzi.de (F.S.); kretzrob@hs-albsig.de (R.K.); clara.chepkirui@helmholtz-hzi.de (C.C.); Kathrin.Wittstein@helmholtz-hzi.de (K.W.); marc.stadler@helmholtz-hzi.de (M.S.); 3 National Centre for Genetic Engineering and Biotechnology (BIOTEC), NSTDA, 113 Thailand Science Park, Phahonyothin Road, Klong Nueng, Klong Luang, Pathum Thani 12120, Thailand; cboonlar@gmail.com (C.B.); sarunyou.wong@biotec.or.th (S.W.); jajen@biotec.or.th (J.L.-a.)

**Keywords:** ascomycota, bacterial pathogens, biofilm dispersion, chromatography, natural products, secondary metabolites, structure elucidation, Xylariales

## Abstract

During the course of our ongoing work to discover new inhibitors of biofilm formation of *Staphylococcus aureus* from fungal sources, we observed biofilm inhibition by cytochalasans isolated from cultures of the ascomycete *Hypoxylon fragiforme* for the first time. Two new compounds were purified by a bioassay-guided fractionation procedure; their structures were elucidated subsequently by nuclear magnetic resonance (NMR) spectroscopy and high-resolution mass spectrometry (HR-MS). This unexpected finding prompted us to test further cytochalasans from other fungi and from commercial sources for comparison. Out of 21 cytochalasans, 13 showed significant inhibition of *Staphylococcus aureus* biofilm formation at subtoxic levels. These findings indicate the potential of cytochalasans as biofilm inhibitors for the first time, also because the minimum inhibitory concentrations (MIC) are independent of the anti-biofilm activities. However, cytochalasans are known to be inhibitors of actin, making some of them very toxic for eukaryotic cells. Since the chemical structures of the tested compounds were rather diverse, the inclusion of additional derivatives, as well as the evaluation of their selectivity against mammalian cells vs. the bacterium, will be necessary as next step in order to develop structure-activity relationships and identify the optimal candidates for development of an anti-biofilm agent.

## 1. Introduction

Biofilm infections are a serious threat in hospitals; following the data of Centers for Disease Control and Prevention Report (2007) [1], around 1.7 million of infections occur per year and 99,000 deaths are caused by infections associated with biofilms. The major problems of biofilms are caused by the resilience of the structured bacterial communities embedded in an extracellular polymeric substance (EPS) matrix containing proteins, DNAs and exopolysaccharides, which offer protection against antimicrobials and the host immune system [2].

*Staphylococcus aureus* is a pathogenic Gram-positive bacterium, present in many diseases, such as: osteomyelitis, periodontitis or peri-implantitis, chronic wound infections, chronic rhinosinusitis, endocarditis, ocular infections and also in polymicrobial biofilm infections. Beyond that, this pathogen is often resistant to antibiotics, increases the infection in indwelling medical devices and contributes to nosocomial infections [3].

We were looking for secondary metabolites for the control of biofilms from various fungal sources, including tropical [4,5] as well as European species [6,7]. The rational assumption is that fungi grow in a wet environment propitious for biofilm development; however, they have developed strategies to protect themselves against biofilms. One of these strategies could be the biosynthesis of secondary metabolites that act as inhibitors of quorum sensing, i.e., the communication of microorganisms through small molecules that coordinate the virulence, formation and maintenance of biofilms [8].

*Hypoxylon fragiforme* is the type species of the genus *Hypoxylon*, which belongs to the family Hypoxylaceae [9], whose species are well-known for their diversity of secondary metabolites [10]. Stromata of *Hypoxylon fragiforme* are constantly associated with beech wood and the fungus actually belongs to the most frequently occurring macromycetes of the northern temperate hemisphere [11]. A previous study on the secondary metabolism of the fungus in different developmental conditions has revealed that the mature stromata contain predominantly azaphilones while the young stromata contain cytochalasins and other yet unidentified compounds [12]. Furthermore, there are reports on mellein derivatives, hypoxyxylerone and other cytochalasins that occur in cultures of the fungus under different fermentation conditions [13,14]. Lately, our research group in collaboration with the Sorbonne University, Paris, has reported several conjugated azaphilones that were first detected in fossil specimens dating back over 1000 years and then isolated from fresh material [15]. 

We have recently also reported using cultures of *H. fragiforme* in malt extract (ME) and potato dextrose (PD) media on the isolation and characterization of sclerin and its diacid, which inhibited the biofilm formation of *S. aureus* [7]. However, extracts from other media also inhibited biofilm and the active principles obtained after bioassay-guided fractionation turned out to be different molecules. The current study is dedicated to the characterization of these active principles and the biological evaluation of chemically similar compounds from different fungal sources. 

## 2. Materials and Methods

### 2.1. Reagents, Media Ingredients and Solvents

Acetonitrile, chloroform, ethyl acetate and methanol were purchased from J. T. Baker (München, Germany) respectively, D-chloroform, formic acid 98%, Casein-soja-peptone (CASO), Potato Dextrose (PD), Luria-Bertani broth (LB), sodium chloride (NaCl), potassium chloride (KCl), potassium dihydrogen phosphate (KH_2_PO_4_), D-methanol, and trifluoroacetic acid (TFA) from Carl Roth GmbH (Karlsruhe, Germany). Bacto malt extract, Bacto peptone and agar were from BD (La Point de Claix, France), D-glucose from Merck (Darmstadt, Germany). Disodium hydrogen phosphate (Na_2_HPO_4_) was purchased from J.T. Baker^®^ (Deventer, The Netherlands), crystal violet from Fluka (Steinheim, Germany), tetracycline and Potato-Dextrose agar (PDA) from Sigma Aldrich (Taufkirchen, Germany) respectively.

### 2.2. Microorganisms

*Staphylococcus aureus* DSM 1104 was purchased from the German Collection of Microorganisms and Cell Cultures (DSMZ, Braunschweig, Germany) and maintained on LB agar at 4 °C.

### 2.3. Fungal Specimens and Cultures Used in the Current Study and Origin of Reference Compounds

The fungus *H. fragiforme* was collected in the Harz Mountains, Germany and its culture was fermented, identified and tested against the biofilms of *S. aureus*, The strain of *H. fragiforme* used in the initial study was the same as reported previously [7]. The culture was derived from ascospores and is maintained under liquid nitrogen at the Helmholtz Centre for Infection Research.

The fermentation conditions used were the same as described previously [7], where mycelia pellets (5 × 5 mm) from *H. fragiforme* grown on malt extract agar (3% malt extract, 0.5% Bacto peptone and 1.5% agar) were transferred to 2 L Erlenmeyer flasks containing 23 mg of Rice (Kaufland, Braunschweig, Germany) and static incubated for 67 days at 22 °C in the dark; this incubation time was necessary for the fungus to grow, synthesize spores and visible secondary metabolites such as hypoxyxylerone [14], and consume the glucose available. After this time, the secondary metabolites were extracted with ethyl acetate and then dried on rotary evaporators. Then, the compounds were dissolved in acetonitrile for the purification and tested for antibiofilm activities.

Following the observations that cytochalasans from *Hypoxylon fragiforme* can inhibit biofilm formations of *S. aureus*, several specimens and mycelial cultures in which such metabolites had been concurrently detected were subjected to scale-up of fermentation and/or extraction of stromata (in case of the Hypoxylaceae) and subsequent isolation of their metabolites. The origin and taxonomy of these fungi is summarised below:

Stromata of *Hypoxylon fragiforme* were collected in the vicinity of Braunschweig, Germany in 2017 by L. Wendt and extracted as described previously [15]. While the more lipophilic fractions were used for isolation of the fragirubrins, which are described in the latter paper, we detected cytochalasin-like compounds by high-performance liquid chromatography-mass spectrometry (HPLC-MS) in the more polar fractions and subjected those to preparative chromatography, which yielded compounds **19**–**20** as described below. A voucher specimen of the material is kept in the fungarium of M. Stadler at the Helmholtz Centre for Infection Research, Braunschweig, Germany (acc. no STMA18022).

Stromata of *Daldinia eschscholtzii* (BBH 42278) and *Daldinia bambusicola* (BBH 42280) as well as *Hypoxylon* cf. *kretzschmariodes* (BBH 42276) were collected in Thailand, Chiang Mai Province, Ban Hua Thung community forest, on decaying wood on 3 November 2016 by P. Srikitikulchai and S. Wongkanoun. Voucher specimens and corresponding cultures are being maintained at the fungarium (BBH) and culture collection (BCC) of BIOTEC (Pathum Thani, Thailand). Aliquots of ca. 500 mg of both specimens were used for extraction, yielding ca. 50 mg of starting material of each specimen for the chromatography experiments that led to the isolation of compounds **9**–**14**. The identification of the specimens followed Stadler et al. [16] 

*Rosellinia rickii* strain (STMA 16008) was obtained from ascospores of a specimen collected by Kathrin Wittstein, Kely Cruz, Esteban B. Sir and Marc Stadler in December of 2015 in the vicinity of Calilegua, Argentina. The specimen is deposited as Fundacion M. Lillo in Tucuman and a corresponding culture is kept at the culture collection of the University of Buenos Aires. The fungus was identified by E. B. Sir using the monograph by Petrini [17]. 

Cytochalasins A–E (**1**–**5**) were purchased from Sigma-Aldrich. Sacchalasin A (**9**) and chaetoglobosin A (**21**) were obtained in the course of our previous studies [18,19].

### 2.4. Purification of the Compounds

Compounds **6** to **8** and **10** to **20** were purified by a preparative HPLC system (Gilson, Middleton, WI, USA) equipped with a GX-271 Liquid handler, a diode array detector (DAD) 172 and a 305 and 306 Pump.

Cytochalasin H (**6**) was separated using the following mobile phase A: H_2_O (Milli-Q, Millipore, Schwalbach, Germany) with 0.05% TFA; B: acetonitrile with 0.05% TFA. The elution gradient was: (i) 55% of solvent B for 3 min; (ii) 55 to 65% of solvent B during 15 min; (iii) 65 to 100% during 3 min; (iv) isocratic condition of 100% solvent B for 5 min. For the separation of the cytochalasins L-696,474 (**7**), the elution gradient started with 75% of solvent B during 3 min; followed by a gradient shift from 75 to 85% of solvent B during 15 min, 85% to 100% of solvent B during 3 min, and isocratic condition of 100% solvent B for 5 min. Ultraviolet (UV) detection was carried out at λ 210, 254 and 350 nm for all the runs.

19,20-Epoxycytochalasin C (**15**; 11 mg), 19,20-epoxycytochalasin D (**16**; 1.6 mg), 19,20-epoxycytochalasin N (**17**; 3.1 mg) and 18-deoxy-19,20-epoxycytochalasin Q (**18**; 1.2 mg) were isolated by preparative HPLC on a VP Nucleodur 100-10-C18 column (150 × 40 mm, 10 μm; Macherey-Nagel, Düren, Germany) using following conditions: solvent A: H_2_O + 0.05% TFA, solvent B: MeCN + 0.05% TFA, gradient: (i) 30–38% B in 8 min; (ii) 38–65% B in 40 min; (iii) 65–100% B in 10 min; flow rate: 30 mL/min.

Compounds **8**, **10**–**14** and **19**–**20** from stromata of *Daldinia* and *Hypoxylon* spp. were isolated by repetitive preparative HPLC. The preparative columns used were from Macherey-Nagel, Düren, Germany and employed depending on the weight of the samples; for crude extracts between 100 mg and 250 mg: VP Nucleodur C18 ec (250 × 40 mm); for crude extracts and intermediate fractions between 10 mg and 100 mg: VP Nucleodur C18 eq. column (250 × 20 mm; Machery-Nagel, Düren, Germany); For crude extracts and intermediate fractions with less than 10 mg: VP Nucleodur C18 ec 10-J-7 (250 × 10 mm). The mobile phase consisted of deionized water (solvent A) and acetonitrile (solvent B) at a flow rate of 20 mL/min. For sample preparation prior to preparative HPLC, the samples were dissolved in acetonitrile and filtered through a Strata X-33 μm polymer reversed phase tube (Phenomenex, Aschaffenburg, Germany) to remove lipids and debris. The fractions of the HPLC were collected in round bottle flasks according to the UV absorption of the chromatogram trace at 210 nm and small aliquots were withdrawn to perform HPLC-diode array detection (DAD)/MS. Then, the acetonitrile was evaporated with a rotary evaporator. The resulting aqueous fractions were frozen and freeze-dried in an Alpha 1–4 LSC freeze dryer (Christ, Osterode, Germany). Samples that appeared pure by electrospray ionization-liquid chromatography mass spectrometry (ESI-LCMS) were subjected to nuclear magnetic resonance (NMR) spectroscopy and high-resolution (HR) mass spectrometry and later on (as their purity and identity were established), to the biological assays.

The identification of the compounds was confirmed by high-resolution electrospray ionisation mass spectrometry (HR-ESIMS), using the same instrumentals setting as reported by Narmani et al. [19]. NMR spectra were recorded on Bruker Avance III 500 MHz spectrometer with a BBFO (plus) SmartProbe (^1^H 500 MHz, ^13^C 126 MHz), and a Bruker Avance III 700 MHz spectrometer with a 5 mm TCI cryoprobe (^1^H 700 MHz, ^13^C 175 MHz). Chemical shifts δ were referenced to the solvents: acetone-*d*_6_ (^1^H, δ = 2.05 ppm; ^13^C, δ = 29.3 ppm), acetonitrile-*d*_3_ (^1^H, δ = 1.94 ppm; ^13^C, δ = 1.9 ppm), chloroform-*d* (^1^H, δ = 7.27 ppm; ^13^C, δ = 77.0 ppm), methanol-*d*_4_ (^1^H, δ = 3.31 ppm; ^13^C, δ = 48.15 ppm).

### 2.5. Spectral Data

#### 2.5.1. Phenochalasin C (**19**)

Colorless oil. [α]^25^_D_ = +0.9 (c 0.2, MeOH). UV (MeOH) λ_max_ (log ε): 226 nm (3.80); 269 nm (3.28). ^1^H NMR (500 MHz, CDCl_3_): see Table 1; ^13^C NMR (125 MHz, CDCl_3_): see Table 1. ESIMS *m*/*z* 450.28 ([M + H]^+^, 448.26 ([M − H]^−^. HR-ESIMS *m*/*z* 450.2638 ([M + H]^+^, calcd for C_28_H_36_NO_4_ 450.2639); 472.2456 ([M + Na]^+^, calcd. for C_28_H_35_NO_4_Na 472.2458).

#### 2.5.2. Phenochalasin D (**20**)

Colorless oil. [α]^25^_D_ = −0.03 (c 0.2, MeOH). UV (MeOH) λ_max_ (log ε): 226 nm (3.74); 254 nm (3.43). ^1^H NMR (700 MHz, CDCl_3_): see Table 1; ^13^C NMR (175 MHz, CDCl_3_): see Table 1. ESIMS *m*/*z* 434.28 ([M + H]^+^, 432.28 ([M − H]^−^. HR-ESIMS *m*/*z* 434.2690 ([M + H]^+^, calcd. for C_28_H_36_NO_3_ 434.2690); 456.2504 ([M + Na]^+^, calcd. for C_28_H_35_NO_3_Na 456.2509).

### 2.6. Bioassays

To analyze the minimal inhibitory concentration (MIC) of the cytochalasans, a pre-inoculum of *S. aureus* was cultivated in LB for 24 h and adjusted to reach the turbidity of 0.5 McFarland, then transferred to microtiter plates, containing serial dilutions of the cytochalasans (256 to 3 μg mL^−1^) dissolved in methanol. The microtiter plates were incubated at 37 °C in a Bioscreen-C automated growth curve analysis system (Oy Growth Curves AB Ltd., Helsinki, Finland). During 24 h, the machine measured each 15 min an optical density at 600 nm (OD_600_) of bacterial growth [7]. To evaluate bactericidic or bacteriostatic effects, aliquots from different concentrations in the wells were inoculated after OD measurements in LB agar for bacterial assays for 24 h. LB medium and methanol were used as negative and tetracycline (100 μg mL^−1^) as positive controls. Experiments were made in triplicate.

For inhibition of biofilm formation, a pre-inoculum of *S. aureus* grown in CASO with 4% of glucose was adjusted to reach the turbidity of 0.5 McFarland and was transferred to 96-well tissue microtiter plates (TPP, Trasadingen, Switzerland), containing serial dilutions of the cytochalasans (256 to 3 μg mL^−1^) dissolved in methanol. Plates were covered with a sterile adhesive porous paper (Kisker Biotech GmbH, Steinfurt, Germany). After 20 h, the biofilms in the microtiter plates were indirectly measured by staining with crystal violet following a published protocol [20]. All experiments were performed in triplicate with two repetitions. 

## 3. Results

### 3.1. Structure Elucidation of the New Compounds

**Figure 1:**
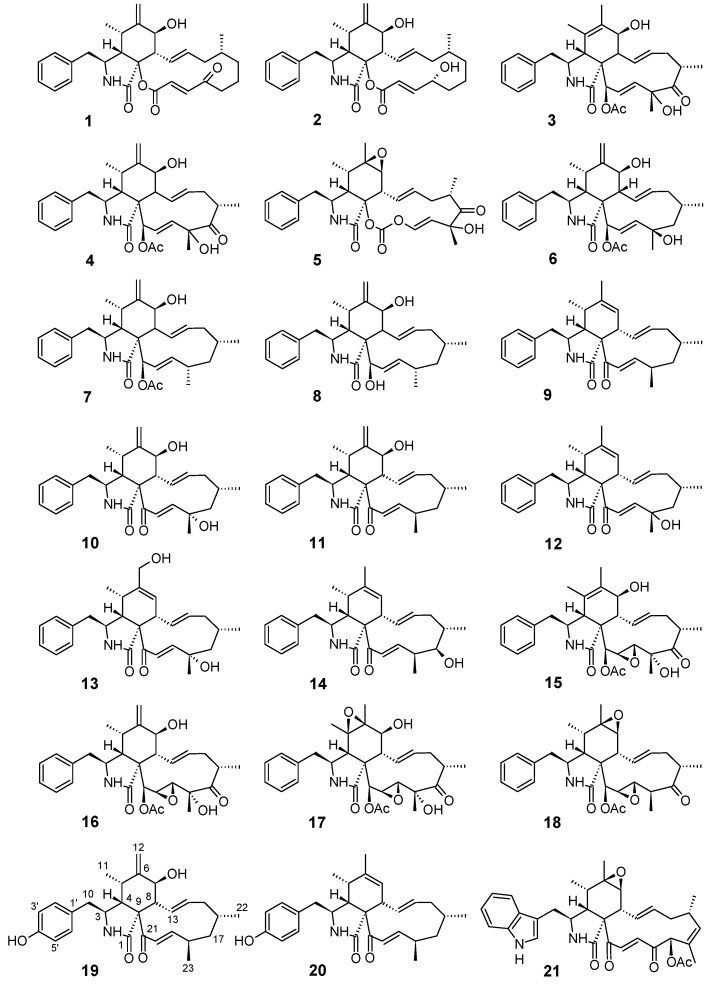
Chemical structures of the cytochalasins that were tested and isolated in the present study. For details of individual molecules see Table 1.

The chemical structures of all cytochalasans tested are depicted in Figure 1, but we here only describe the structure elucidation of the novel natural products that were obtained in the course of our study. Their NMR data are depicted in Table 1.

Metabolite **19** was isolated as a colorless oil by reversed-phase HPLC from a fruiting body extract of *Hypoxylon* cf. *kretzschmarioides* BBH 42276. Its molecular formula C_28_H_35_NO_4_ was deduced from its [M + H]^+^ and [M + Na]^+^ peaks at *m/z* 450.2634 and 472.2456, respectively. ^1^H and heteronuclear single quantum correlation (HSQC) NMR spectra revealed the presence of three methyls, an exocyclic as well as three aliphatic methylenes, and six olefinic (two with dual intensity) as well as seven aliphatic methines. In addition, the ^13^C spectrum indicated a conjugated ketone, a carboxylic carbon, and four further carbons devoid of bound protons. A large spin system was constructed by ^1^H,^1^H correlation spectroscopy (COSY) and total correlation spectroscopy (TOCSY) correlations ranging from 7–H/8–H/13–H/14–H/15–H_2_/16–H(22–H_3_)/17–H_2_/18–H(23–H_3_)/19–H/20–H, in addition to smaller ones from 11–H_3_/5–H/4–H/3–H/10–H_2_ and 2′–H/3′–H, respectively. These spin systems were connected by heteronuclear multiple bond correlations (HMBC), especially to note those from 12–H_a/b_ to C–5/C–6/C–7, from 8–H to C–1/C–4/C–9/C–21, from 19–H and 20–H to C–21, from 4–H to C–1/C–6/C–8/C–9/C–21 and from 2′/6′–H to C–4′/C–10, to form a cytochalasin skeleton. Its closest structural relative is (7*S*,13*E*,16*S*,18*R*,19*E*)-16,18-dimethyl-7-hydroxy-10-phenyl-[11]-cytochalasa-6(12),13,19-triene-1,21-dione, the 4′-dehydroxyderivative of **19** [21]. Because the ^13^C chemical shifts of the main backbone are virtually indistinguishable, an identical stereochemistry was concluded for **19**. The stereochemistry was supported by rotating-frame nuclear Overhauser effect correlation spectroscopy (ROESY) data, since ROESY correlations between 13–H and 20–H as well as 14–H and 19–H supported the typical conformation for the eleven membered ring system [22]. ROESY correlations between 23–H_3_ and 16–H and 19–H, which are located above the molecular main plain, confirm the upwards orientations of 23–H_3_ and thus an 18*S* configuration.

For the structural similarities to other compounds of this class in which a tyrosin rather than a phenylalanin moiety has been incorporated into the cytochalasin backbone, we propose the generic name phenochalasin C for compound **19** due to structural resemblance to phenochalasins A and B [23]. Its systematic name is (7*S*,13*E*,16*S*,18*R*,19*E*)-16,18-dimethyl-7-hydroxy- 10-(4′-hydroxyphenyl)-[11]-cytochalasa-6(12),13,19-triene-1,21-dione [24].

Metabolite **20** was analyzed for its molecular formula C_28_H_35_NO_3_ by HR-ESIMS, indicating the formal loss of an oxygen atom compared to **19**. The NMR data of **20** were highly similar to those of **19**. Key differences, as indicated by ^1^H and HSQC data, were the replacement of exomethylene CH_2_–12 by a methyl and oxymethine CH–7 by an olefinic methine, respectively. Consequently, **19** is the 4′-hydroxyderivative of sacchalasin A (**9**), isolated from a fruiting body of *Daldinia sacchari* [19]. Its systematic name is (6*Z*,13*E*,16*S*,18*R*,19*E*)-16,18-dimethyl-7-hydroxy- 10-phenyl-[11]-cytochalasa6,13,19-triene-1,21-dione [24], and was named phenochalasin D.

Further known cytochalasans were isolated from other species of Sordariomycetes and identified by comparing the ^1^H and ^13^C chemical shifts and the HRMS data to those reported previously ([25] for **6**; [26] for **7**, [27] for **8**; [28] for 1**0**, [21] for **11**–**13**, [27] for **15**–**18**). However, upon comparison of our NMR data with those published previously [26,27] for l-696,474 (**7**) and 21-*O*-deacetyl-l-696,474 (**8**), respectively, it was found that ^1^H and ^13^C assignments for methyl groups CH_3_-22 and CH_3_-23 were different to those in the literature. As deduced from the HMBC spectra of **8** it is obvious that the methyl signal at δ_H_ 1.04 correlates to an olefinic carbon at δ_C_ 134.3, assigned as the 23–H_3_/C–19 correlation. Subsequently, the carbon chemical shift δ_C_ 22.4 for CH_3_–23 was assigned due to its HSQC correlation to δ_H_ 1.04. The HMBC correlations of CH_3_–22 (δ_H_ 1.01/δ_C_ 25.2) to C–15/C–16/C–17 at δ_C_ 42.3; 33.5 and 48.3, respectively, confirm this assignment. For l-696,474 (**7**), methyl groups CH_3_–22 and CH_3_–23 were reassigned analogously.

### 3.2. Anti-Biofilm Activities of the Tested Cytochalasins

Although cytochalasins showed very weak to no antimicrobial activities against *S. aureus*, with cytochalasin A (**1**) being the only active metabolite with at a MIC of 32 μg mL^−1^ (bacteriostatic effect), they presented higher effects against biofilms from this bacterium at subtoxic levels (Table 2). Because compound **1** was the only one that showed such moderate bacteriostatic activities, we have analysed the sample from Sigma and included the NMR and HPLC-MS data in the supporting information (see Figures S2 and S3). The compound showed over 90% purity, but we cannot exclude that the observed impurities may have contributed to the observed antibacterial effects.

Cytochalasin A (**1**), the two derivatives **10**, **12** and **13** from *Daldinia eschscholtzii* as well as chaetoglobosin A (**21**) were the most potent ones as they inhibited 70–91% of biofilm formation in *Staph aureus*. Chaetoglobosin A (**21**) inhibited 85% of the biofilm at one third of its MIC and 61% of the biofilm at one-eighth of its MIC, while the other mentioned compounds **10**, **12** and **13** were active at a concentration of 256 μg mL^−1^ around 74–85% and at 128 μg mL^−1^ inhibited 45–55% the biofilm formations at a MIC higher than 256 μg mL^−1^. Furthermore, compound **1** inhibited 91% of the biofilm with half of the MIC.

Cytochalasin C (**3**), L-696,474 (**7**), 19,20-epoxycytochalasin C (**15**), and the new phenochalasin D (**20**) inhibited around 40–60% of the biofilm also at subtoxic levels. Cytochalasin A (**1**) and L-696,474 (**7**), inhibited 44% and 91% of biofilm formation, respectively at 16 μg mL^−1^, demonstrating a good antibiofilm potential at low concentrations. The other cytochalasans tested showed a weak 20–40% or no effect against the biofilms. The results indicate that the biofilm inhibitions were independent of the MIC, as reported previously [7,29].

In relation to the chemical structure of cytochalasans, these preliminary results are not very easy to interpret, but should give rise to further studies. The results obtained indicate that in some cytochalasins an isomeric double bond could destroy the ability to block biofilms (e.g., **15** and **16**). Acetylation can considerably increase this activity (**8** to **7**), as a phenol does at C-4′ (e.g., **11** to **19** or **9** to **20**), while epoxidations seem to have a negative effect (e.g., **15** to **17**). No attempts were made as yet to determine the mode of action of the compounds, since this would definitely afford extensive additional experimental work that would go beyond the scope of the present study. However, we speculate that the target of cytochalasans is probably not in the synthesis of the cell wall polymers since only bacteriostatic but no bacteriocidal effects have been observed. As the production of exocellular polymeric substances (EPS) is drastically reduced under the influence of certain cytochalasins, their production and/or export may be the main target. As no mechanism of action is known, it is even possible that these compounds may have different targets in the bacterial cell complicating any structure activity relations even further. For instance, the fact that chaetoglobosin A (**21**) was the only compound tested that has a tryptophan (rather than phenylalanin or tyrosin) incorporated, but turned out to be one of the most potent metabolites, suggests that further metabolites of this type should be tested in the future. A comparison of the activities of the series of compounds from *R. rickii* (**15**–**18**) suggests that the neither an epoxide nor an exomethylene group in the six-membered ring is favorable for the anti-biofilm activity, and the only compound (**15**) of the epoxy cytochalasin series that showed significant activity was still less potent than some metabolites that are devoid of the epoxide at C–19/C–20. The significant differences in the potency of the known cytochalasins **1**–**6** and the highly similar compounds from species of Hypoxylaceae (**6**–**14** and **19**–**20**) are rather difficult to explain.

## 4. Discussion

Cytochalasans are fungal polyketide-non ribosomal peptides, characterized by a substituted isoindole scaffold fused with a macrocyclic ring, derived from a highly reduced polyketide backbone and an amino acid [30]. The first cytochalasans was discovered during the 1960s [31,32] and until now more than 300 cytochalasans are estimated to have been described and isolated from many fungi.

Cytochalasans are known to exhibit diverse effects in biological systems. For some cytochalasans, e.g., cytochalasin H (**6**), antibacterial activities have been reported but mainly against Gram-negative pathogens [33], while only cytochalasin A (**1**) was moderately active against the Gram-positive *Bacillus subtilis* [34]. Cytochalasins A (**1**) and D (**4**) acted against the pathogenic fungus *Botrytis cinerea* [34] and several cytochalasans, e.g., cytochalasin E (**5**), displayed cytotoxic activities [35,36,37]. For L-696,474 (**7**), inhibition of HIV-1 protease was reported [26]. However, in particular, cytochalasans are famous for the capping of actin filaments. As a consequence, the cytokinesis is blocked and the nuclear division is not affected, resulting in multinucleated cells [38,39,40]. If the cells are exposed at higher concentrations, they become denucleated [38]. These properties are being exploited for the use of cytochalasans as biochemical tools in the study of cell cycles, the cytoskeleton, cell adhesion, motility, signalling and cytokinesis [39,40]. Some studies showed that cytochalasans exhibit high cytotoxicity in prelinical trials; however, two classes of cytochalasin have recently been discovered, the cytotoxic and the cytostatic cytochalasans [41,42]. The cytostatic cytochalasin have shown good potential in cancer therapies [43,44].

Herein we reported for the first time the cytochalasans acting against biofilms of *S. aureus*. The cytochalasans showed weak antibacterial activities, in accordance with Betina et al. [30]. However, in contrast, the inhibition of biofilm formation of some compounds were really impressive, showing high (70–90%), good (40–60%) and moderate (20–40%) activities demonstrating that these compounds interfere with biofilm formation, since the MIC were independent of the antibiofilm activity as described in our previous studies [4,6,7]. Cytochalasans add to the broad diversity of compounds blocking *S. aureus* biofilm development, e.g., coprinuslactone [6], Roussoellic acid [5], RNAIII inhibiting peptide (RIP) [45] or usnic acid [46].

The fungus *Hypoxylon fragiforme* synthesizes different inhibitors of biofilms, depending on the culture media in which it was grown. Interestingly, the production of cytochalasins was previously reported to be greatly enhanced in nature during spring and early summer in the growing stromata and the anamorph of the fungus that colonised the woody substrate {12]. They disappeared from the stromata as the ascospores became mature and the characteristic azaphilone pigments [10,15] were produced instead. This phenomenon could be attributed to the fact that the fungus uses the metabolites as a means of defence against competing organisms. Since fungi are competing in nature not only with numerous other fungi, but even with bacteria, it would constitute a selective advantage if their secondary metabolites can address different molecular target sites in different organism groups. Previously it was reported [7] that sclerin and its diacid-produced Potato Dextrose/Malt extract media, have specific activities against the biofilms of *S. aureus*. When this fungus was fermented in other media (Rice/Minimal medium) compounds other than cytochalasans, were formed and also inhibited the formation of *S. aureus* biofilms. This provides evidence that the inhibition of bacterial biofilms is really important for the fungus, because even changing the composition of the media, the fungus synthesizes compounds which block biofilm formation, confirming in this way our hypothesis that fungi protect themselves by producing compounds against infections involving biofilms.

Therefore, cytochalasans can be used not only mainly to study the cell cycles, but also to control biofilm infections, since some cytochalasans, such as **10** inhibited up to 85% of the biofilm formations of *S*. *aureus* at concentrations of 64–16 μg mL^−1^, with a MIC higher than 256 μg mL^−1^, demonstrating the potential of this activity. Many molecular studies are necessary to discover the mechanisms of inhibition of biofilm formations, but if the cytochalasans are combined with antibiotics [47,48], this can be a promising strategy to treat staphylococcal infections.

## 5. Conclusions

Various biological activities are known for cytochalasans, but those can mainly be associated by their interference with the cytoskeleton of eukaryotes. Here, we report for the first time an activity of these compounds against biofilm formation of the prokaryotic *S. aureus*. This suggests that the compounds address different target sites in fungi and bacteria. Inhibitions of the biofilm formations were really significant, inhibiting in high (70–91%), good (40–70%) and moderate (20–40%) levels. For all the inhibitions, MICs were independent of the biofilm inhibitions, indicating that the compounds only inhibit the biofilm formation but did not kill the bacterium. The mechanism of inhibition still remains to be elucidated, e.g., by testing the compounds on their activities against key proteins of quorum sensing in *S. aureus*. Their combination with antibiotics may become good alternative for the treatment of staphyloccocal infections in the future, in case cytochalasins with low toxicity and strong biofilm inhibition can be found. For this purpose, additional derivatives of the cytochalasan type should be made available for testing, and it could also be worthwhile to study this phenomenon in other bacteria. Since actin is not present in bacteria, the target site in *S. aureus* must be different and remains to be discovered. Our next step will be to test the compounds concurrently against mammalian cells for actin inhibition and add further derivatives to the cytochalasin library in an attempt to get better data that allow for evaluation of structure–activity relationships. At the same time, the search for additional inhibitors of biofilm formation in fungi appears promising, and even well-known classes of fungal metabolites may turn out to have potential.

## Figures and Tables

**Table 1:** Nuclear magnetic resonance (NMR) data of new metabolites **18**–**20** in CHCl_3_-*d*.

	19 ^a^		20 ^b^	
	δ_C_, Mult.	δ_H_, Mult.	δ_C_, Mult.	δ_H_, Mult.
1	173.6, C		174.3, C	
2		5.49, br s		5.42, br s
3	53.3, CH	3.25, m	55.0, CH	3.20, m
4	44.8, CH	3.30, dd (5.7, 2.4)	48.4, CH	3.20, m
5	31.7, CH	2.79, m	34.8, CH	2.43, m
6	148.6, C		140.2, C	
7	71.6, CH	4.10, d (10.1)	125.7, CH	5.48, m
8	51.8, CH	2.44, m	49.7, CH	2.58, d (9.6)
9	63.4, C		68.4, C	
10	43.3, CH_2_	2.63, dd (13.4, 5.2) 2.43, m	44.2, CH_2_	2.73, dd (13.7, 4.4) 2.41, dd (13.7, 8.8)
11	13.2, CH_3_	1.02, m	13.5, CH_3_	1.18, br d (7.3)
12	114.1, CH_2_	5.28, br s 5.09, br s	20.0, CH_3_	1.75, q (1.3)
13	126.9, CH	5.85, dd (15.6, 9.8)	128.1, CH	5.85, ddd (15.5, 9.6, 1.3)
14	138.7, CH	5.22, ddd (15.6, 10.9, 4.8)	135.9, CH	5.22, ddd (15.5, 10.9, 4.8)
15	42.9, CH_2_	2.02, m 1.81, m	42.7, CH_2_	2.01, m 1.78, m
16	28.7, CH	1.45, m	28.7, CH	1.48, m
17	46.7, CH_2_	1.94, m 1.55, m	46.4, CH_2_	1.95, m 1.52, m
18	34.3, CH	2.63, m	34.4, CH	2.63, m
19	154.75, CH	6.52, dd (15.9, 6.9)	153.2, CH	6.46, dd (15.9, 6.9)
20	132.1, CH	7.05, br d (15.9)	132.9, CH	7.12, dd (15.9, 1.4)
21	196.6		198.1, C	
22	26.2, CH_3_	1.04, m	26.1, CH_3_	1.02, d (6.9)
23	17.6, CH_3_	1.14, d (7.0)	17.6, CH_3_	1.13, d (6.9)
1′	129.2, C		129.5, C	
2′/6′	130.3, CH	6.99, br d (8.4)	130.3, CH	6.99, br d (8.4)
3′/5′	115.6, CH	6.77, br d (8.4)	115.6, CH	6.77, br d (8.4)
4′	154.69, C	OH: 5.41, s	154.6, C	OH: 5.09, s

^a^ 500 Mhz for ^1^H, 125 MHz for ^13^C, ^b^ 700 Mhz for ^1^H, 175 MHz for ^13^C.

**Table 2:** Origin and anti-biofilm activities of the tested cytochalasans. BCC: BIOTEC culture collection, BBH: BIOTEC Bangkok Herbarium.

Compound	Source, Producing Fungus/Strain	MIC (μg mL^−1^)	Inhibition of Biofilm Formation (%)	Potency of Biofilm Inhibition ^1^
Cytochalasin A (**1**)	*Drechslera dermatioidea* (Sigma)	32^S^	91 ± 1.4 (16 μg mL^−1^)	+++
Cytochalasin B (**2**)	*Drechslera dermatioidea* (Sigma)	>256	-	-
Cytochalasin C (**3**)	*Metarrhizium anisopliae* (Sigma)	>256	42 ± 6.2 (256 μg mL^−1^) 27 ± 2.9 (128 μg mL^−1^) 21 ± 2.3 (64 μg mL^−1^)	++
Cytochalasin D (**4**)	*Zygosporium mansonii* (Sigma)	>256	-	-
Cytochalasin E (**5**)	*Aspergillus clavatus* (Sigma)	>256		-
Cytochalasin H (**6**) [12,25]	*Hypoxylon fragiforme* (cultures)	>256	-	-
L-696,474 (**7**) [26]	*Hypoxylon fragiforme* (cultures)	>256	44 ± 0.02 (64 μg mL^−1^) 46 ± 1.2 (32 μg mL^−1^) 44 ± 0.05 (16 μg mL^−1^)	++
21-*O*-Deacyl-l-696,474 (**8**) [27]	*Hypoxylon fragiforme* (stromata)	>256	33 ± 9.1 (256 μg mL^−1^) 28 ± 15.3 (64 μg mL^−1^)	+
Saccalasin A (**9**) [19]	*Daldinia bambusicola* BCC 42280*Daldinia sacchari*	>256	36 ± 8.8 (256 μg mL^−1^) 33 ± 4.3 (128 μg mL^−1^) 14 ± 1.8 (32 μg mL^−1^)	+
**10** [21]	*Daldinia eschscholtzii* BBH 42278	>256	85 ± 5.4 (256 μg mL^−1^) 54 ± 6.0 (128 μg mL^−1^)	+++
**11** [22]	*Daldinia eschscholtzii* BBH 42278	>256	-	-
**12** [23]	*Daldinia eschscholtzii* BBH 42278	>256	76 ± 10.8 (256 μg mL^−1^) 51 ± 4.8 (128 μg mL^−1^)	+++
**13** [23]	*Daldinia eschscholtzii* BBH 42278	>256	73.7 ± 16.8 (256 μg mL^−1^) 44.8 ± 17.0 (128 μg mL^−1^) 30.6 ± 15.6 (64 μg mL^−1^) 26.8 ± 13.5 (4 μg mL^−1^)	+++
**14** [23]	*Daldinia eschscholtzii* BBH 42278	>256	32 ± 0.7 (256 μg mL^−1^)	+
19,20-Epoxycytochalasin C (**15**) [28]	*Rosellinia rickii* (culture)	>256	40 ± 6.0 (256 μg mL^−1^) 22 ± 12.6 (128 μg mL^−1^)	++
**16**–**18** [28]	*Rosellinia rickii* (culture)	>256	-	-
Phenochalasin C (**19**)	*Hypoxylon* cf. *kretzschmarioides* BBH 42276	>256	31 ± 6.4 (256 μg mL^−1^) 14 ± 1.7 (128 μg mL^−1^)	+
Phenochalasin D (**20**)	*Hypoxylon* cf. *kretzschmarioides* BBH 42276	>256	43 ± 4.0 (256 μg mL^−1^)46 ± 1.5 (64 μg mL^−1^) 33 ± 6.7 (32 μg mL^−1^) 15 ± 2.0 (16 μg mL^−1^)	++
Chaetoglobosin A (**21**) [18]	*Ijuhya vitellina* (culture)	256	87.3 ± 4.4 (128 μg mL^−1^) 85.1 ± 4.7 (64 μg mL^−1^) 61.2 ± 1.4 (32 μg mL^−1^) 17.8 ± 1.1 (8 μg mL^−1^)	+++

^1^ +++ 70–91%, ++ 40–70%, + 20–40% biofilm inhibition.

## Supplementary Materials

Supplementary materials can be found at http://www.mdpi.com/2218-273X/8/4/129/s1, Figure S1: COSY, TOCSY, HMBC and ROESY correlations indicating the structures of phenochalasin C (**19**) and phenochalasin D (**20**); Table S1: NMR data (^1^H 500 MHz, ^13^C 125 MH) of **7** in CDCl_3_, Table S2: NMR data (^1^H 700 MHz, ^13^C 175 MH) of **8** in CDCl_3_, Figure S2: HPLC-HRESIMS data of cytochalasin A (**1**); Figure S3: ^1^H NMR spectrum (700 MHz, CDCl_3_) of cytochalasin A (**1**); Figure S4: ^1^H NMR spectrum (500 MHz, CDCl_3_) of cytochalasin H (**6**); Figure S5: ^13^C NMR spectrum (125 MHz, CDCl_3_) of cytochalasin H (**6**); Figure S6: ^1^H NMR spectrum (500 MHz, CDCl_3_) of L-696,474 (**7**); Figure S7: ^13^C NMR spectrum (125 MHz, CDCl_3_) of L-696,474 (**7**); Figure S8: HSQC NMR spectrum (500 MHz, CDCl_3_) of L-696,474 (**7**); Figure S9: HMBC NMR spectrum (500 MHz, CDCl_3_) of L-696,474 (**7**); Figure S10: ^1^H NMR spectrum (700 MHz, CDCl_3_) of 21-*O*-Deacetyl-l-696,474 (**8**); Figure S11. ^13^C NMR spectrum (175 MHz, CDCl_3_) of 21-*O*-Deacetyl-l-696,474 (**8**); Figure S12: COSY NMR spectrum (700 MHz, CDCl_3_) of 21-*O*-Deacetyl-l-696,474 (**8**); Figure S13: HSQC NMR spectrum (700 MHz, CDCl_3_) of 21-*O*-Deacetyl-l-696,474 (**8**); Figure S14: HMBC NMR spectrum (700 MHz, CDCl_3_) of 21-*O*-Deacetyl-l-696,474 (**8**); Figure S15: Detail of the HMBC NMR spectrum (700 MHz, CDCl_3_) of 21-*O*-Deacetyl-l-696,474 (**8**); Figure S16. ^1^H NMR spectrum (500 MHz, CDCl_3_) of compound **10**; Figure S17: ^13^C NMR spectrum (125 MHz, CDCl_3_) of compound **10**; Figure S18: ^1^H NMR spectrum (500 MHz, CD_3_CN) of compound **11**; Figure S19: ^13^C NMR spectrum (125 MHz, CD_3_CN) of compound **11**; Figure S20: ^13^C NMR spectrum (175 MHz, CDCl_3_) of compound **11**; Figure S21: ^1^H NMR spectrum (700 MHz, CDCl_3_) of compound **12**; Figure S22: ^13^C NMR spectrum (175 MHz, CDCl_3_) of compound **12**; Figure S23. ^1^H NMR spectrum (700 MHz, CDCl_3_) of compound **13**; Figure S24: ^1^H NMR spectrum (125 MHz, CDCl_3_) of compound **13**; Figure S25: ^1^H NMR spectrum (700 MHz, CDCl_3_) of compound **14**; Figure S26: ^13^C NMR spectrum (175 MHz, CDCl_3_) of compound **14**; Figure S27: ^1^H NMR spectrum (500 MHz, DMSO-*d*_6_) of 19,20-epoxycytochalasin C (**15**); Figure S28: ^13^C NMR spectrum (125 MHz, DMSO-*d*_6_) of 19,20-epoxycytochalasin C (**15**); Figure S29: ^1^H NMR spectrum (500 MHz, CH_3_OH-*d*_4_) of 19,20-epoxycytochalasin D (**16**); Figure S30: ^13^C NMR spectrum (125 MHz, CH_3_OH-*d*_4_) of 19,20-epoxycytochalasin D (**16**); Figure S31:. ^1^H NMR spectrum (500 MHz, CH_3_OH-*d*_4_) of 19,20-epoxycytochalasin N (**17**); Figure S32: ^13^C NMR spectrum (125 MHz, CH_3_OH-*d*_4_) of 19,20-epoxycytochalasin N (**17**); Figure S33: ^1^H NMR spectrum (500 MHz, acetone-*d*_6_) of 18-deoxy-9,20-epoxycytochalasin Q (**18**); Figure S34: ^13^C NMR spectrum (175 MHz, acetone-*d*_6_) of 18-deoxy-9,20-epoxycytochalasin Q (**18**); Figure S35:. HPLC-HRESIMS data of phenochalasin C (**19**); Figure S36: ^1^H NMR spectrum (500 MHz, CDCl_3_) of phenochalasin C (**19**); Figure S37: ^13^C NMR spectrum (125 MHz, CDCl_3_) of phenochalasin C (**19**); Figure S38: COSY NMR spectrum (500 MHz, CDCl_3_) of phenochalasin C (**19**); Figure S39: ROESY NMR spectrum (500 MHz, CDCl_3_) of phenochalasin C (**19**); Figure S40. HSQC NMR spectrum (500 MHz, CDCl_3_) of phenochalasin C (**19**); Figure S41: HMBC NMR spectrum (500 MHz, CDCl_3_) of phenochalasin C (**19**); Figure S42: HPLC-HRESIMS data of phenochalasin D (**20**); Figure S43: ^1^H NMR spectrum (700 MHz, CDCl_3_) of phenochalasin D (**20**); Figure S44: ^13^C NMR spectrum (175 MHz, CDCl_3_) of phenochalasin D (**20**); Figure S45: COSY NMR spectrum (700 MHz, CDCl_3_) of phenochalasin D (**20**); Figure S46: ROESY NMR spectrum (700 MHz, CDCl_3_) of phenochalasin D (**20**); Figure S47: HSQC NMR spectrum (700 MHz, CDCl_3_) of phenochalasin D (**20**); Figure S48: HMBC NMR spectrum (700 MHz, CDCl_3_) of phenochalasin D (**20**).
