# Supplementary material for: Cytochalasans Act as Inhibitors of Biofilm Formation of Staphylococcus aureus
Source: Biomolecules. 2018 Oct 30;8(4):129. doi: 10.3390/biom8040129 (PMC6316226; doi:10.3390/biom8040129)
Supplement: Supplementary file 1 [file biomolecules-08-00129-s001.pdf]

Article

Cytochalasans act as inhibitors of biofilm formation in *Staphylococcus aureus*

Kamila Tomoko Yuyama[1,2], Lucile Wendt[2], Frank Surup[2], Robin Kretz[2], Clara Chepkirui[2], Kathrin Wittstein[2], Chollaratt Boonlarppradab[3], Sarunyou Wongkanoun[3], Jennifer Luangsa-ard[3], Marc Stadler[2] and Wolf-Rainer Abraham[1,*]

[1] Dept. Chemical Microbiology, Helmholtz Centre for Infection Research (HZI), Inhoffenstraße 7, 38124 Braunschweig, Germany; kamila.yuyama@helmholtz-hzi.de, wolf-rainer.abraham@helmholtz-hzi.de
[2] Dept. Microbial Drugs, Helmholtz Centre for Infection Research (HZI), Inhoffenstraße 7, 38124 Braunschweig, Germany ; lucile.wendt@helmholtz-hzi.de, Frank.Surup@helmholtz-hzi.de, kretzrob@hs-albsig.de, clara.chepkirui@helmholtz-hzi.de, Kathrin.Wittstein@helmholtz-hzi.de, marc.stadler@helmholtz-hzi.de
[3] National Centre for Genetic Engineering and Biotechnology (BIOTEC), NSTDA, 113 Thailand Science Park, Phahonyothin Road, Klong Nueng, Klong Luang, Pathum Thani, 12120 Thailand; cboonlar@gmail.com, sarunyou.wong@biotec.or.th, jajen@biotec.or.th
* Correspondence: wolf-rainer.abraham@helmholtz-hzi.de; Tel.: +49-531-6181-4300

-------------------- Supporting Information --------------------

19

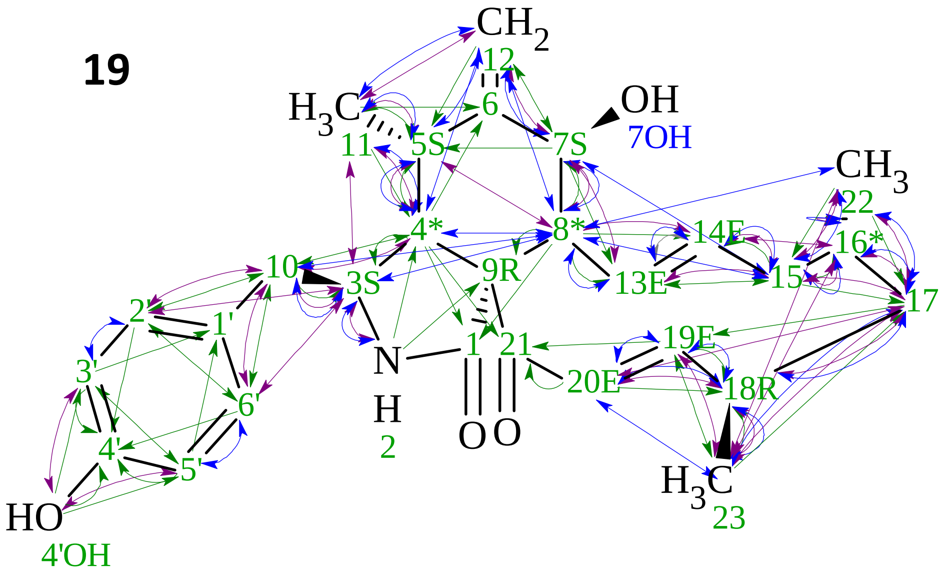

20

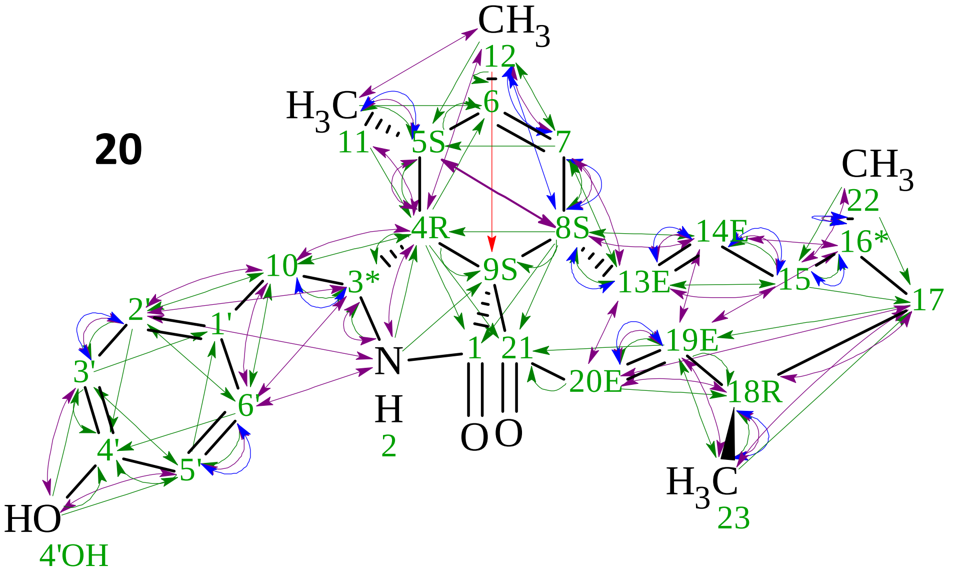

Figure S1. COSY and TOCSY (blue arrows), HMBC (green arrows) and ROESY (violet arrows) correlations indicating the structures of phenochalasin C (**19**) and phenochalasin D (**20**).

Table S1. NMR data (^{1}H 500 MHz, ^{13}C 125 MH) of **7** in $CDCl_3$.

Atom#	C Shift	XHn	H Shift	H Multiplicity	C to H HMBC
1'	137.49	C			
2', 6'	128.99	CH	7.16	d (7.0)	10, 4'
3	53.73	CH	3.27	m	
3', 5'	128.94	CH	7.33	m	1'
4	50.63	CH	2.14	m	
4'	127.05	CH	7.25	m	3', 5'
5	32.95	CH	2.82	br s	
6	148.02	C			
7	69.52	CH	3.84	br d (10.8)	
8	47.24	CH	2.93	s	
10	45.70	CH2	2.64	dd (13.4, 9.9)	4, 3, 3', 5', 1'
10	45.70	CH2	2.89	br dd (13.4, 4.3)	3, 3', 5', 1'
11	14.27	CH3	1.04	d (6.7)	5, 4, 6
12	113.92	CH2	5.37	s	5, 7
12	113.92	CH2	5.13	s	5, 7
13	127.44	CH	5.78	br dd (15. 6, 10.1)	
14	138.39	CH	5.35	br s	
15	42.47	CH2	1.78	br s	16, 17
15	42.47	CH2	2.00	br dd (12.5, 4.6)	22, 16, 17, 13, 14
16	33.32	CH	1.39	m	
17	48.39	CH2	1.39	m	
17	48.39	CH2	1.62	m	
18	34.18	CH	2.13	br s	
19	135.79	CH	5.72	br ddd (16.5, 7.1, 1.8)	
20	125.38	CH	5.96	br d (16.5)	
21	78.54	CH	5.57	br s	8, 20, 19, 24
22	25.25	CH3	1.009	m	16, 15, 17
23	22.07	CH3	1.012	d (6.9)	17, 19
24	170.13	C			
25	20.89	CH3	2.24	s	21, 24
3NH		NH	5.47	br s	

Table S2. NMR data (^{1}H 700 MHz, ^{13}C 175 MH) of **8** in $CDCl_3$.

Atom#	C Shift	XHn	H Shift	H Multiplicity	COSY	N/ROESY	C to H HMBC
1	175.70	C					
1'	137.71	C					
2		NH	5.41	br s		3, 2', 6'	4, 9, 3
2', 6'	129.08	CH	7.17	br d (7.3)	3', 5'	10, 10, 3, 2	10, 4'
3	53.79	CH	3.30	m	10, 4, 10	11, 4, 10, 12, 2', 6', 2	5, 4, 1', 1
3', 5'	128.89	CH	7.33	m	2', 6', 4'		1'
4	50.14	CH	2.65	t (4.4)	3	11, 8, 10, 3, 21	11, 5, 10, 9, 21, 6, 1
4'	127.03	CH	7.26	m	3', 5'		2', 6'
5	33.04	CH	2.93	m	11	11, 8	11
6	148.55	C					
7	69.73	CH	3.85	br d (10.8)	8, 12, 12	12, 13, 7OH	12, 13, 6
7OH		OH	2.06	s		7	8, 7, 6
8	46.09	CH	2.84	t (10.2)	7, 13	4, 5, 14	9, 7, 21, 13, 14, 6, 1
9	53.04	C					
10	45.72	CH2	2.60	dd (13.6, 9.9)	10, 3	10, 21, 2', 6'	4, 3, 3', 5', 2', 6', 1'
10	45.72	CH2	2.94	m	10, 3	11, 10, 4, 3, 2', 6'	4, 3, 2', 6', 1'
11	14.16	CH3	1.14	d (6.7)	5	4, 5, 10, 3, 12	5, 4, 6
12	113.62	CH2	5.35	s	7, 12	7	5, 7, 6
12	113.62	CH2	5.13	s	7, 12	11, 3	5, 7, 6
13	127.97	CH	5.79	dd (15.6, 9.8)	8, 14	17, 7	15, 8, 7
14	137.75	CH	5.27	br d (5.0)	21OH, 15, 13, 15	16, 15, 8	16, 15, 8
15		CH2	1.76	br s	14		16, 17, 13, 14
15	42.26	CH2	1.98	br dd (12.7, 4.7)	21OH, 14	22, 16, 17, 21OH, 14	22, 16, 17, 13, 14
16	33.45	CH	1.37	m	22, 17	15, 18, 14, 19	17
17	48.26	CH2	1.38	m	17	22, 23, 17, 15, 18, 19	22, 16
17	48.26	CH2	1.64	m	16, 17, 18	23, 17, 18, 13, 20	18, 15, 19
18	34.09	CH	2.18	br d (4.1)	23, 17	23, 16, 17, 17, 19	23, 20, 19
19	134.26	CH	5.93	m	20	16, 17, 18, 20	23, 18, 21
20	131.17	CH	6.21	dd (16.7, 2.7)	21, 19	23, 17, 21, 19	18, 21
21	77.38	CH	4.20	m	21OH, 20	10, 4, 20	8, 4, 9, 20, 19
21OH	42.26	OH	1.77	m	15, 21, 14	15	9, 21
22	25.15	CH3	1.01	m	16	17, 15	16, 15, 17
23	24.40	CH3	1.04	m	18	17, 17, 18	17, 18, 19

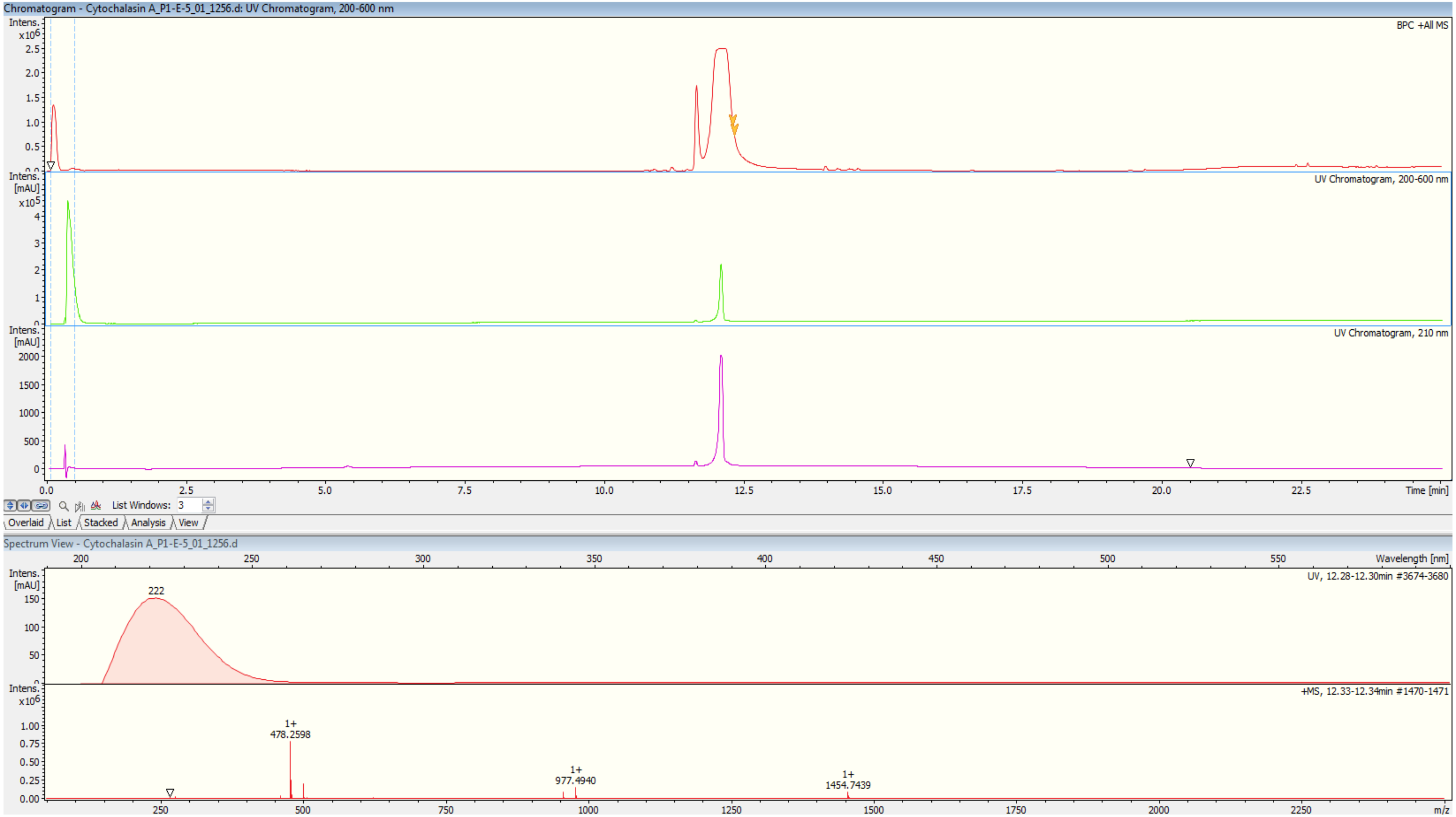

Figure S2. HPLC-HRESIMS data of cytochalasin A (**1**).

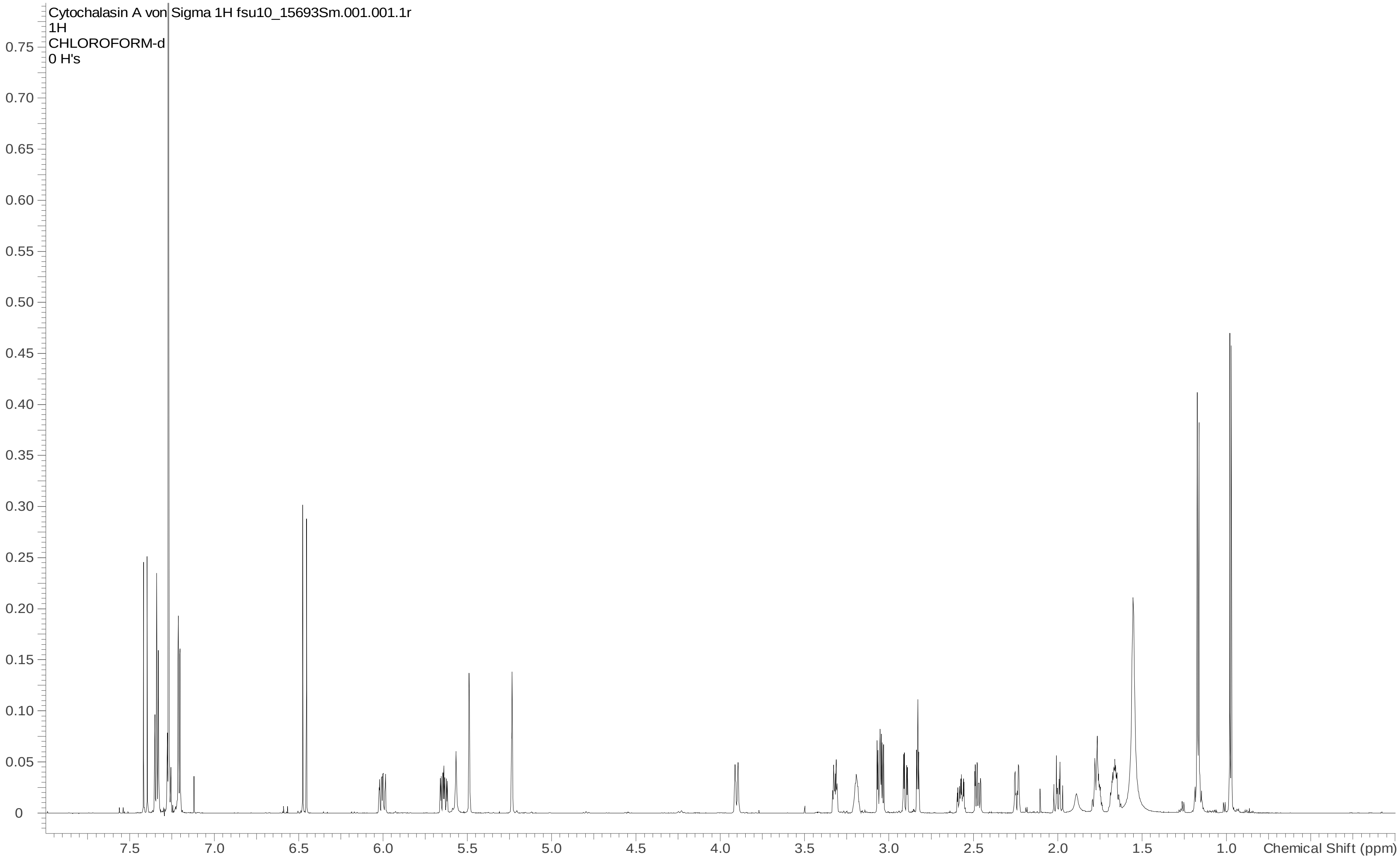

Figure S3. ^{1}H NMR spectrum (700 MHz, $CDCl_3$) of cytochalasin A (**1**).

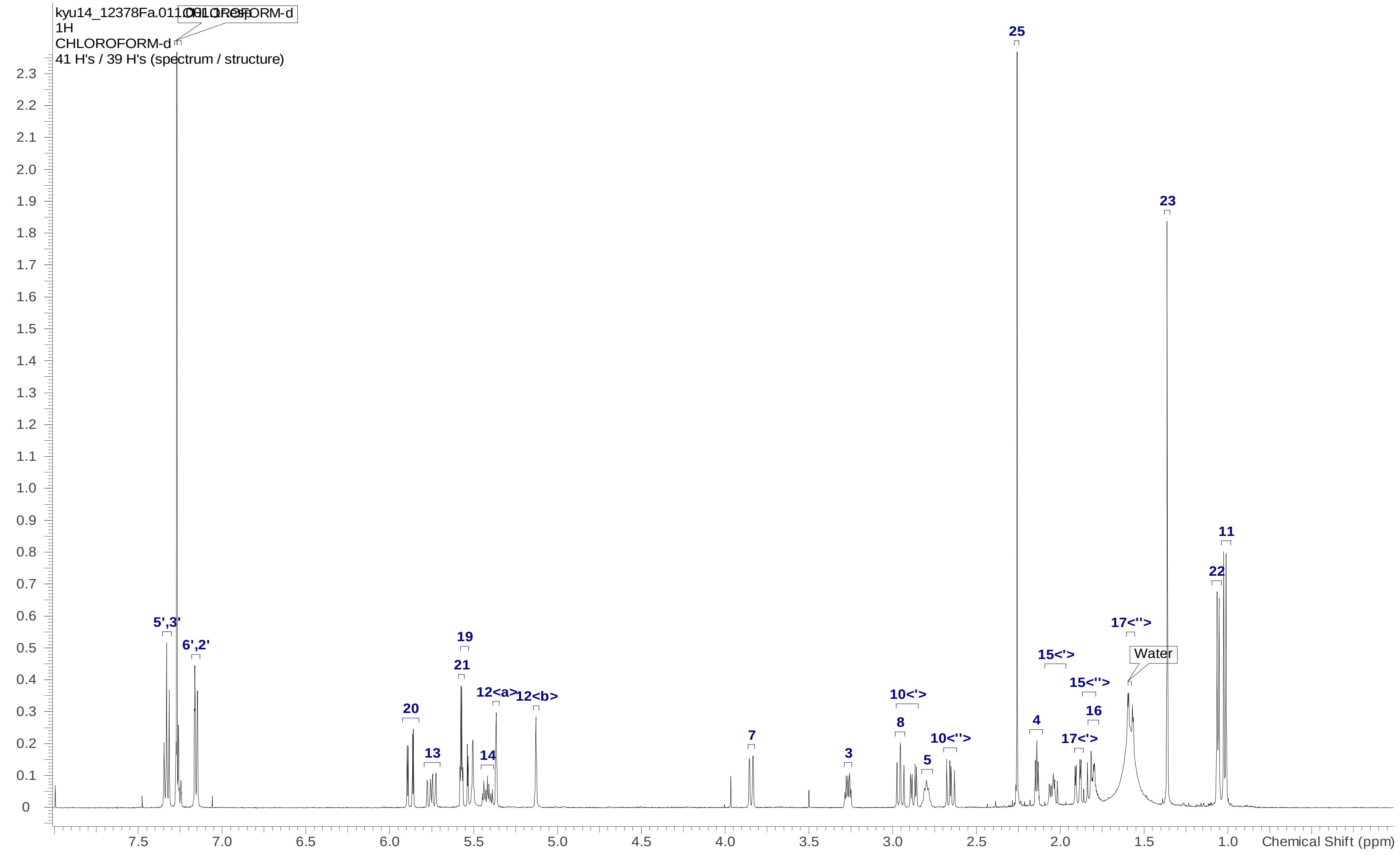

Figure S4. ^{1}H NMR spectrum (500 MHz, $CDCl_3$) of cytochalasin H (**6**).

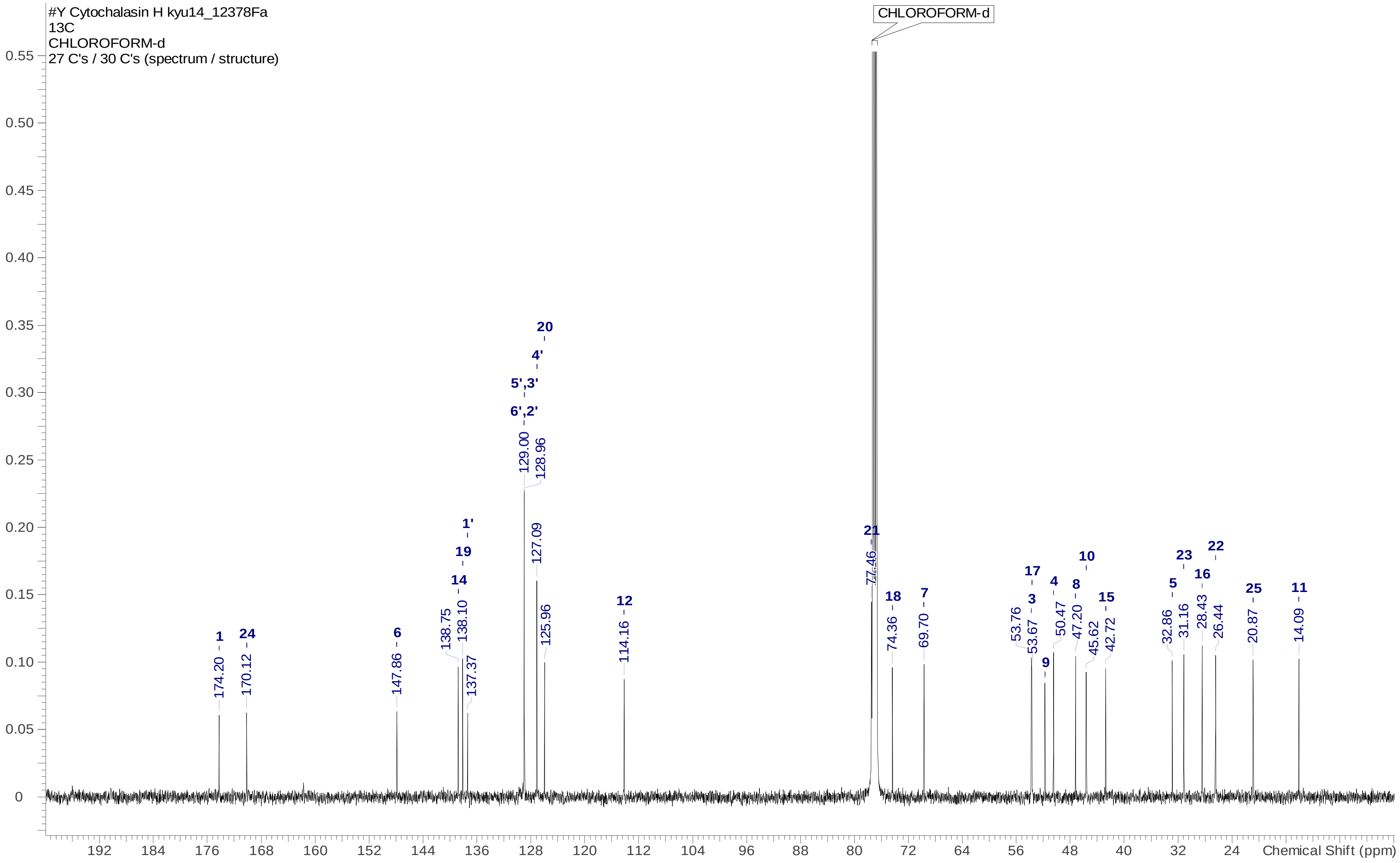

Figure S5. ^{13}C NMR spectrum (125 MHz, $CDCl_3$) of cytochalasin H (**6**).

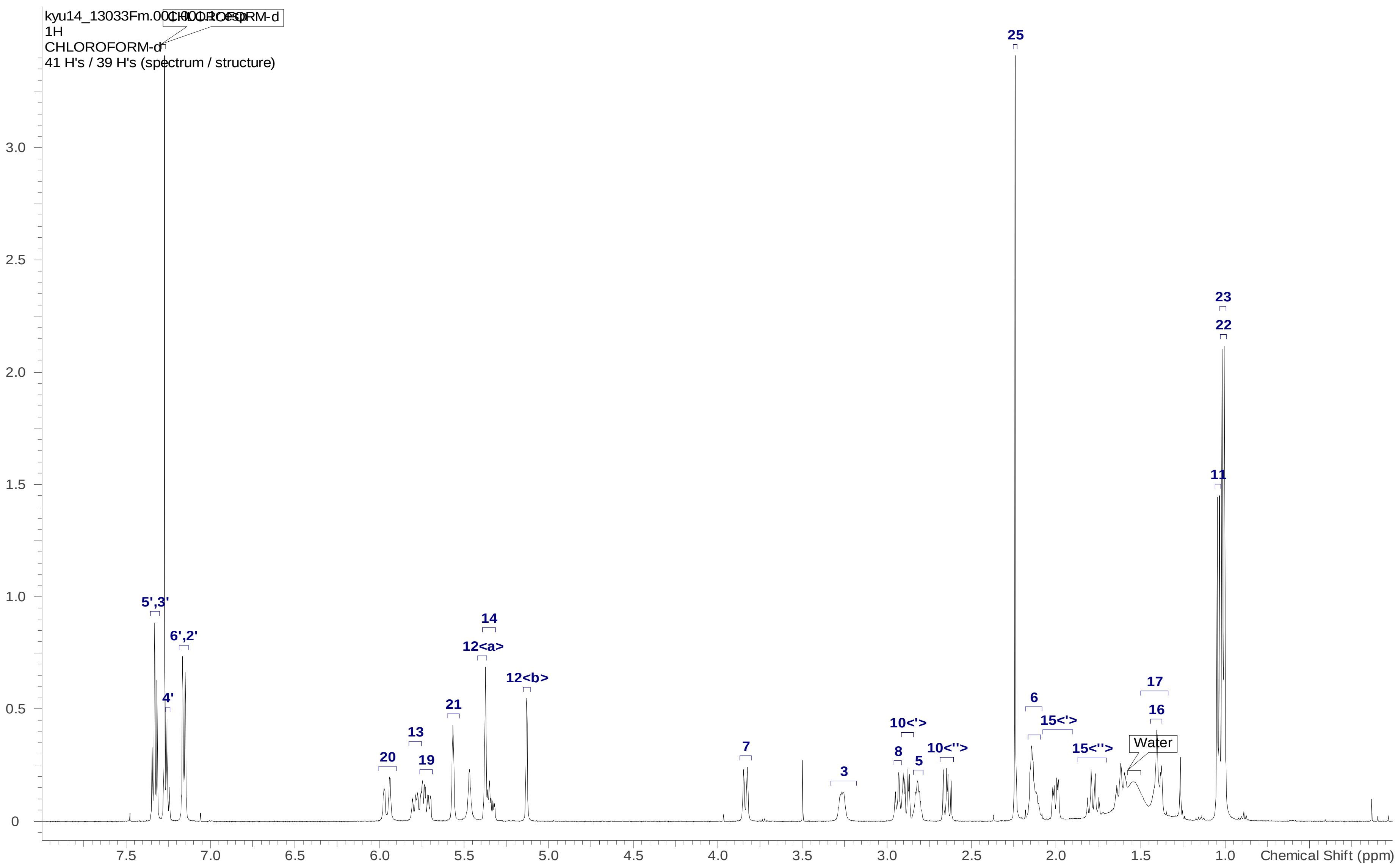

Figure S6. ^{1}H NMR spectrum (500 MHz, $CDCl_3$) of L-696,474 (**7**).

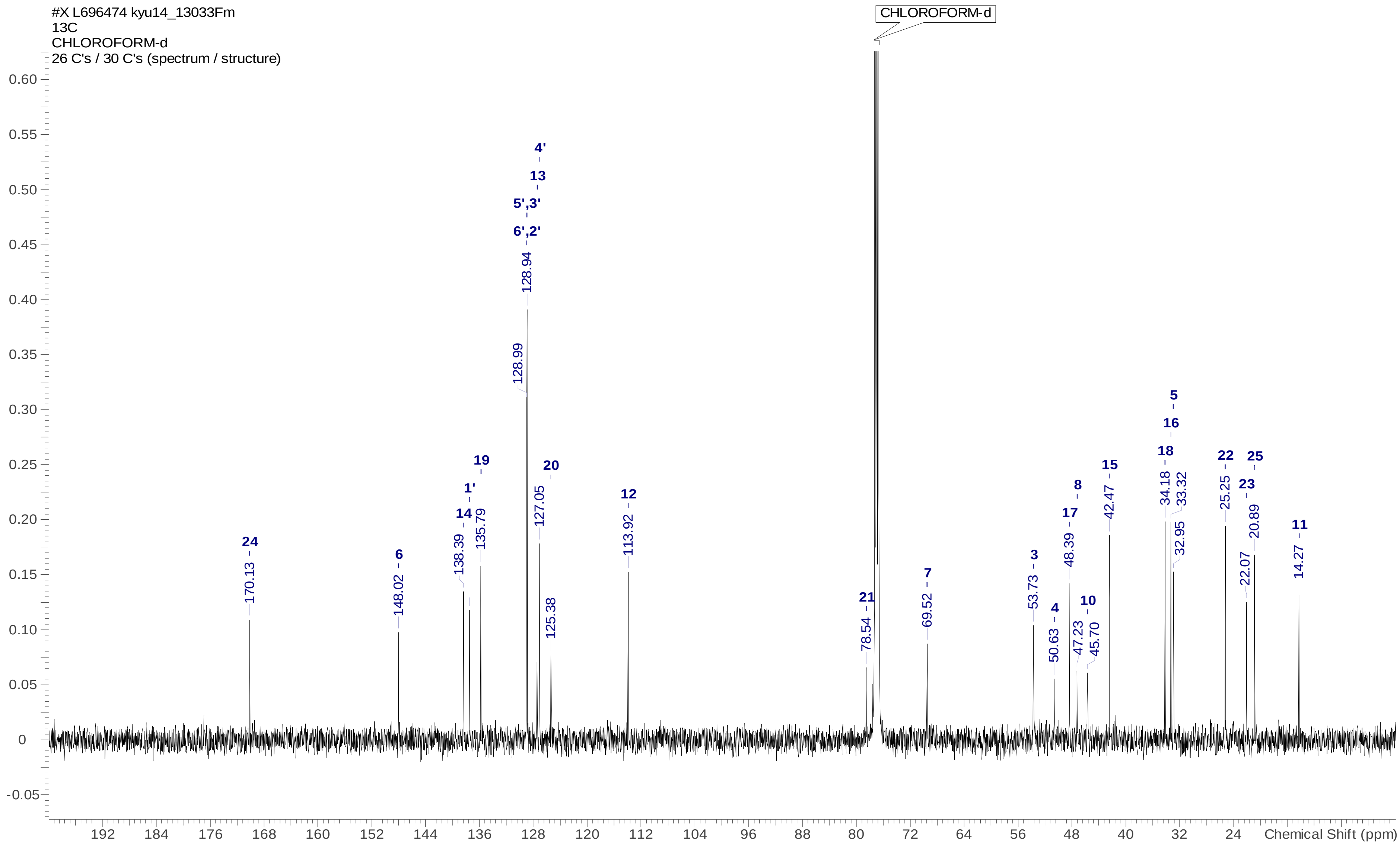

Figure S7. ^{13}C NMR spectrum (125 MHz, $CDCl_3$) of L-696,474 (**7**).

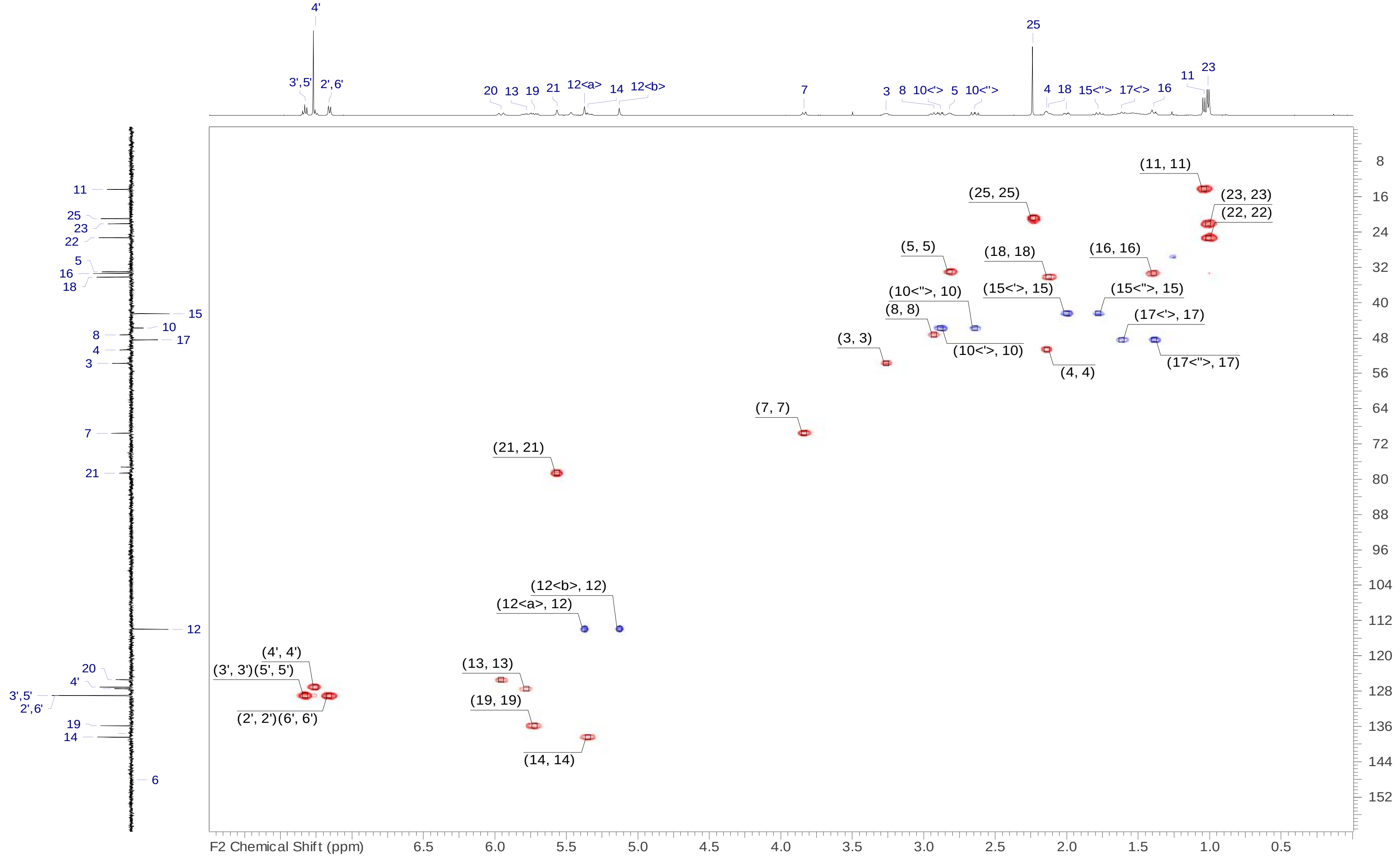

Figure S8. HSQC NMR spectrum (500 MHz, $CDCl_3$) of L-696,474 (**7**).

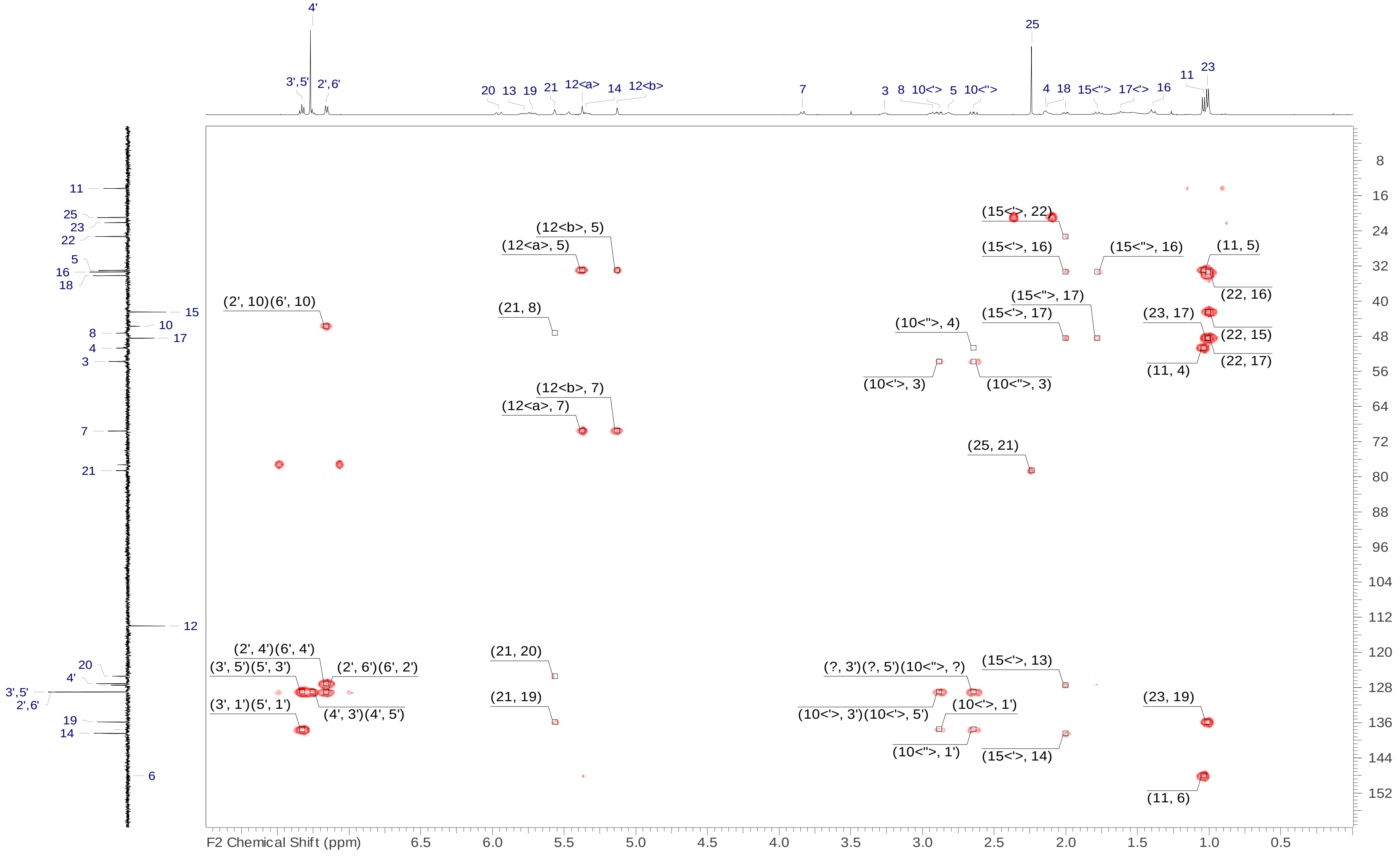

Figure S9. HMBC NMR spectrum (500 MHz, $CDCl_3$) of L-696,474 (**7**).

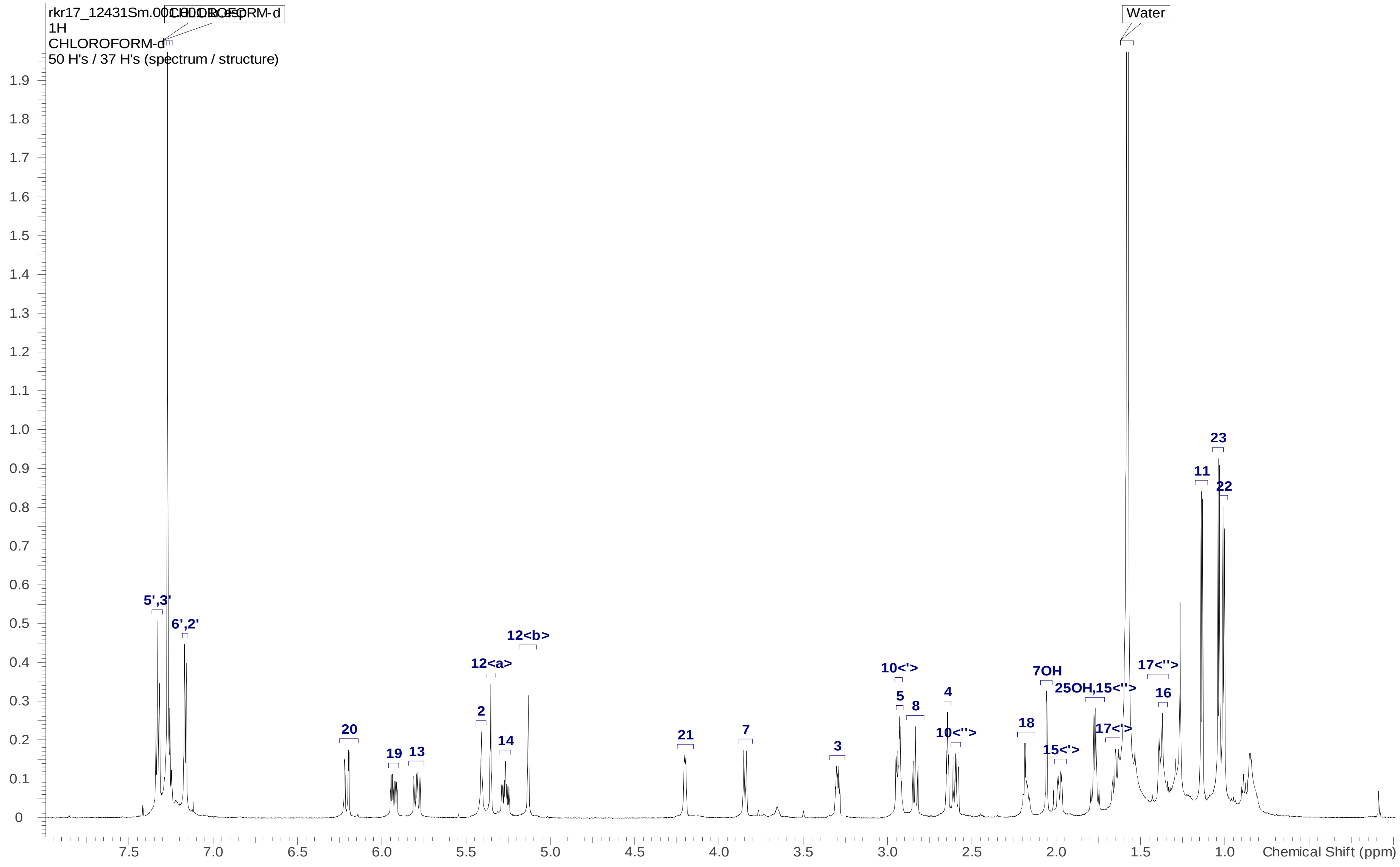

Figure S10. ^{1}H NMR spectrum (700 MHz, $CDCl_3$) of 21-*O*-Deacetyl-L-696,474 (**8**).

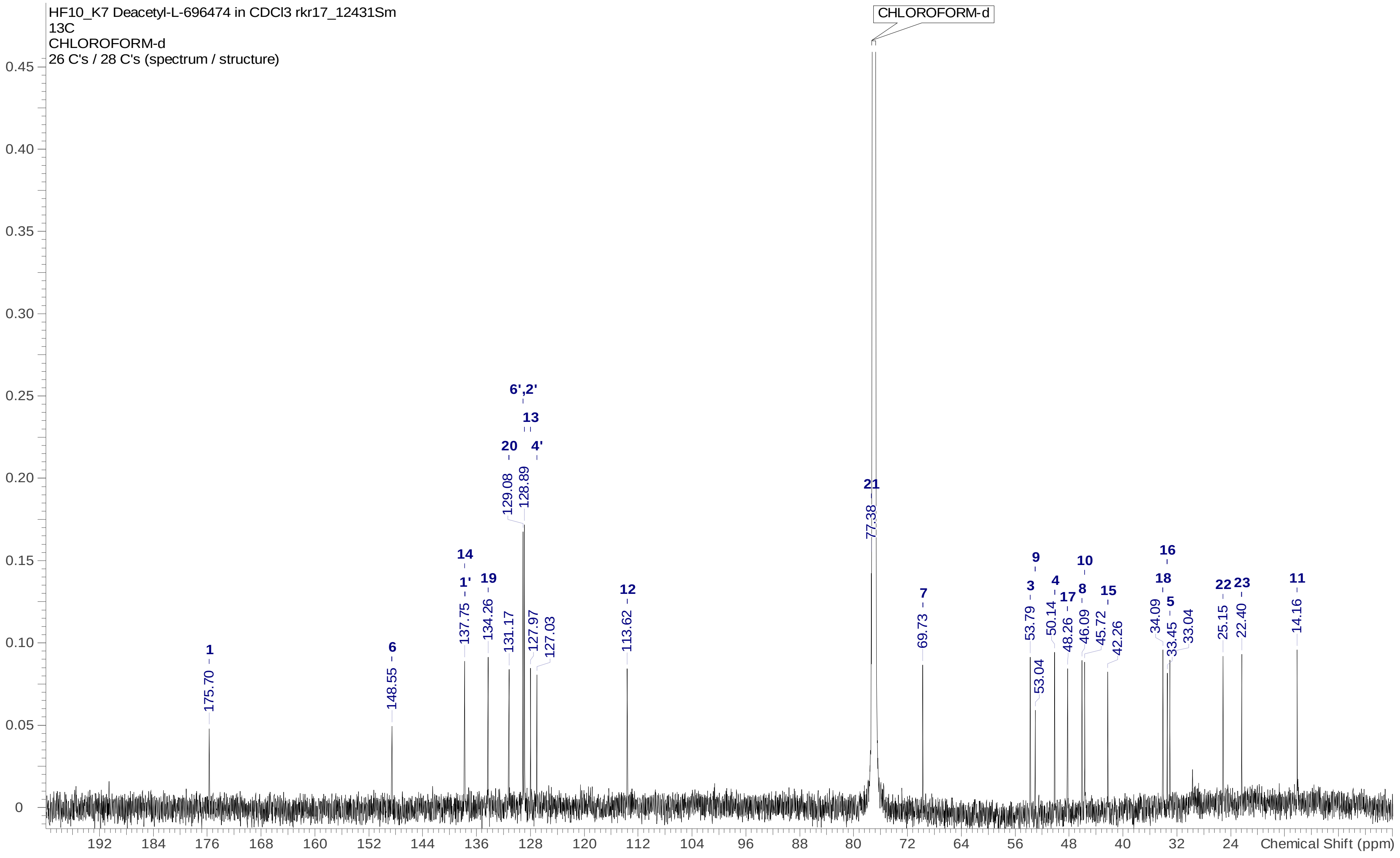

Figure S11. ^{13}C NMR spectrum (175 MHz, $CDCl_3$) of 21-*O*-Deacetyl-L-696,474 (**8**).

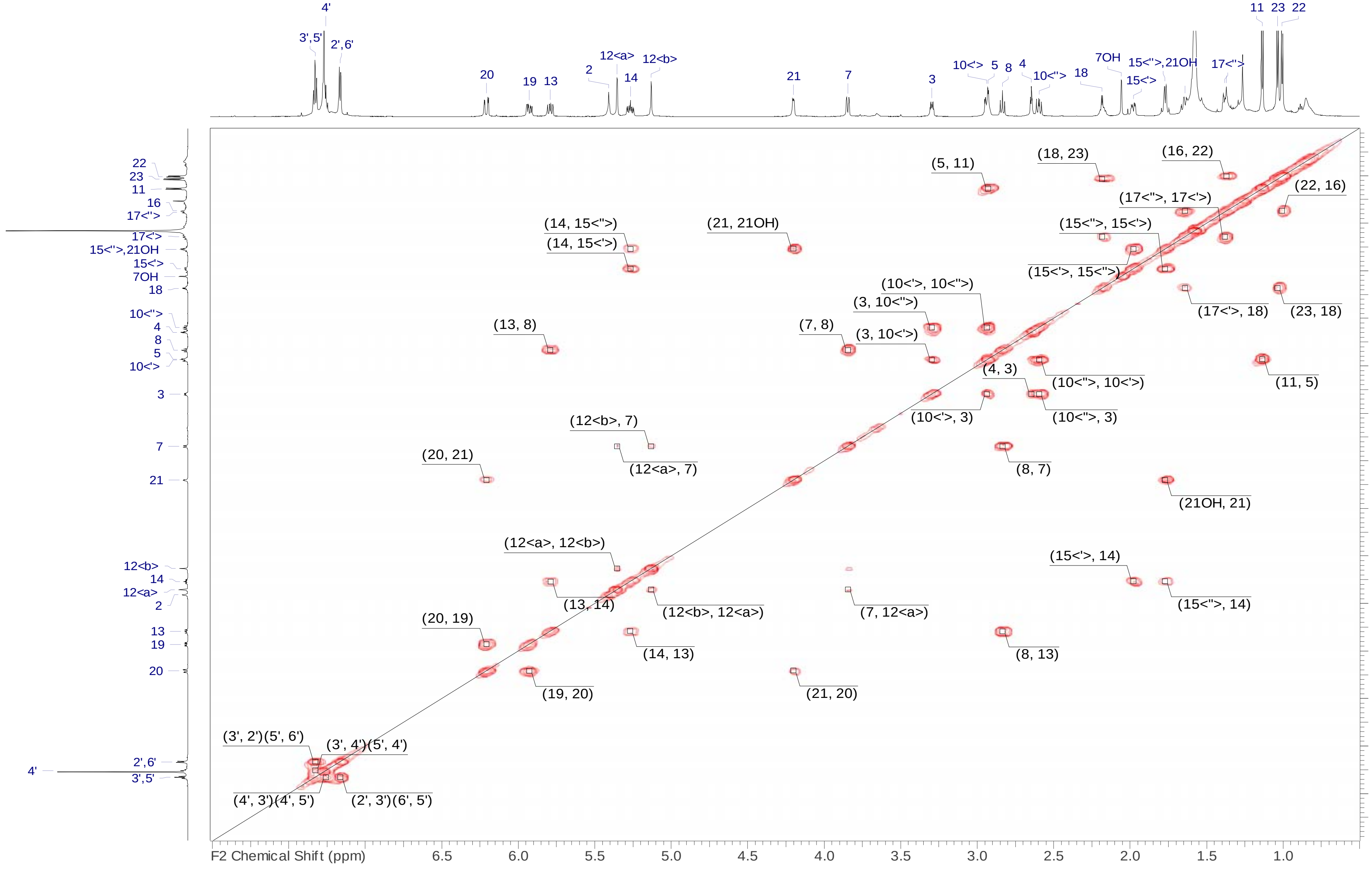

Figure S12. COSY NMR spectrum (700 MHz, $CDCl_3$) of 21-*O*-Deacetyl-L-696,474 (**8**).

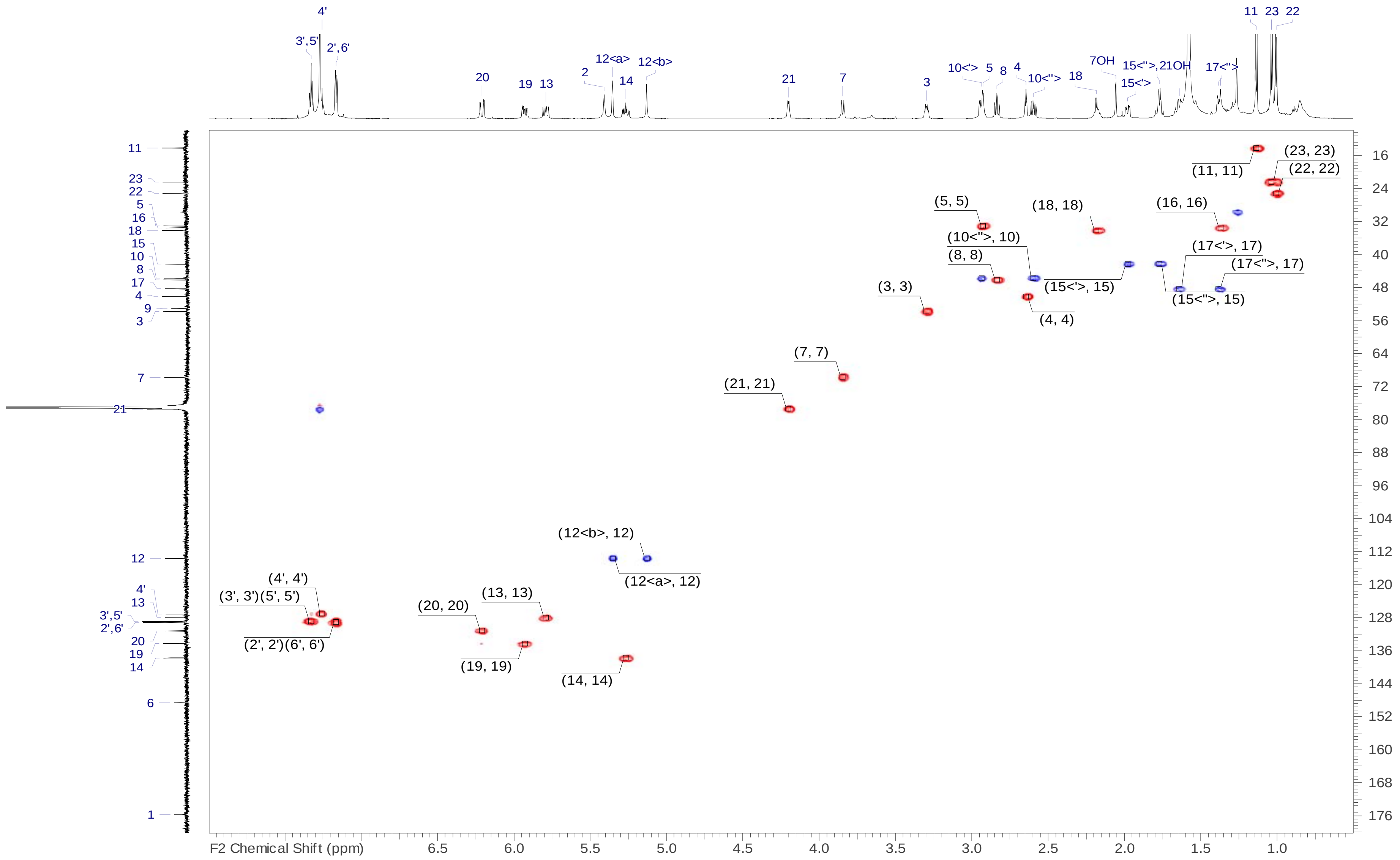

Figure S13. HSQC NMR spectrum (700 MHz, $CDCl_3$) of 21-*O*-Deacetyl-L-696,474 (**8**).

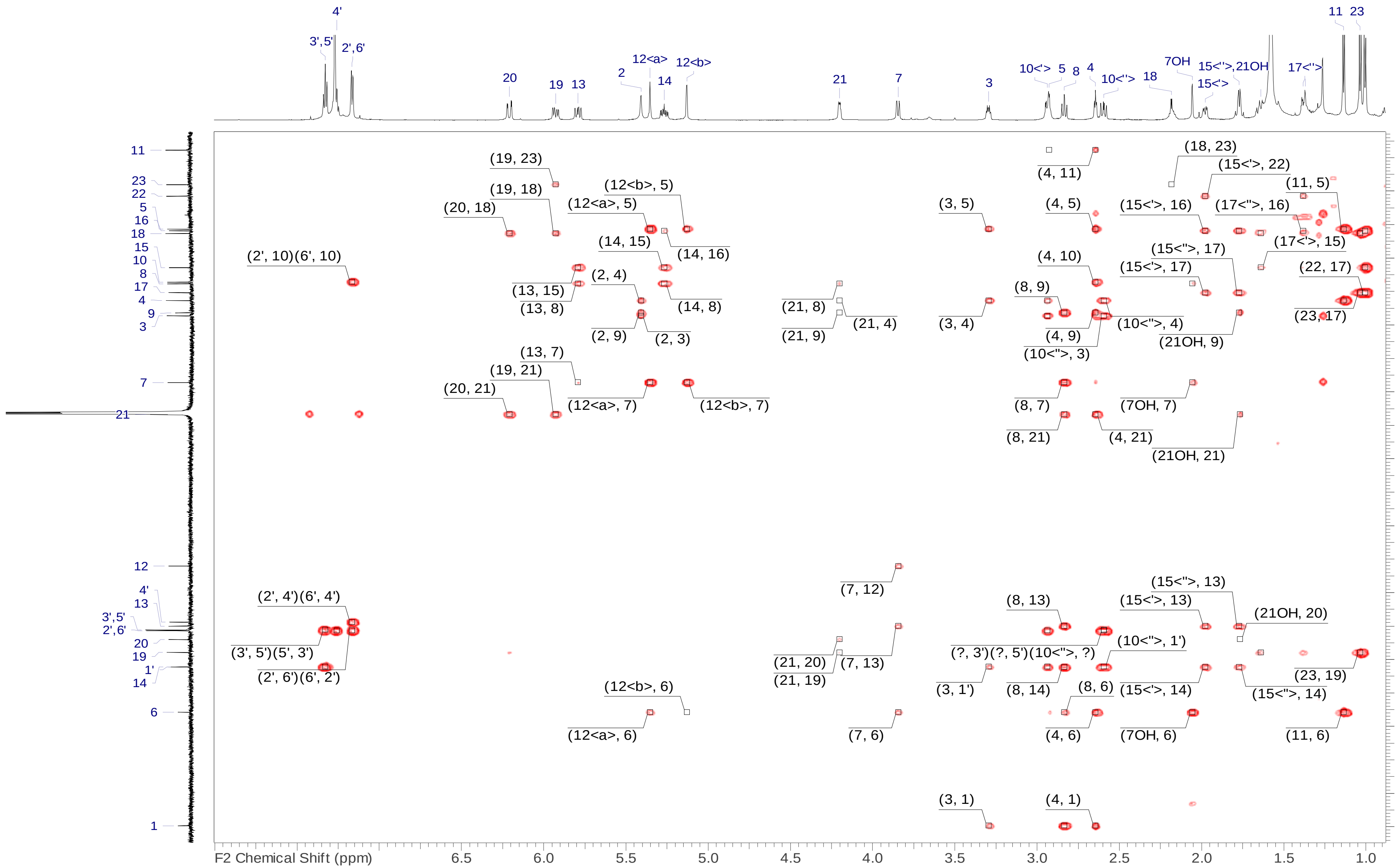

Figure S14. HMBC NMR spectrum (700 MHz, $CDCl_3$) of 21-*O*-Deacetyl-L-696,474 (**8**).

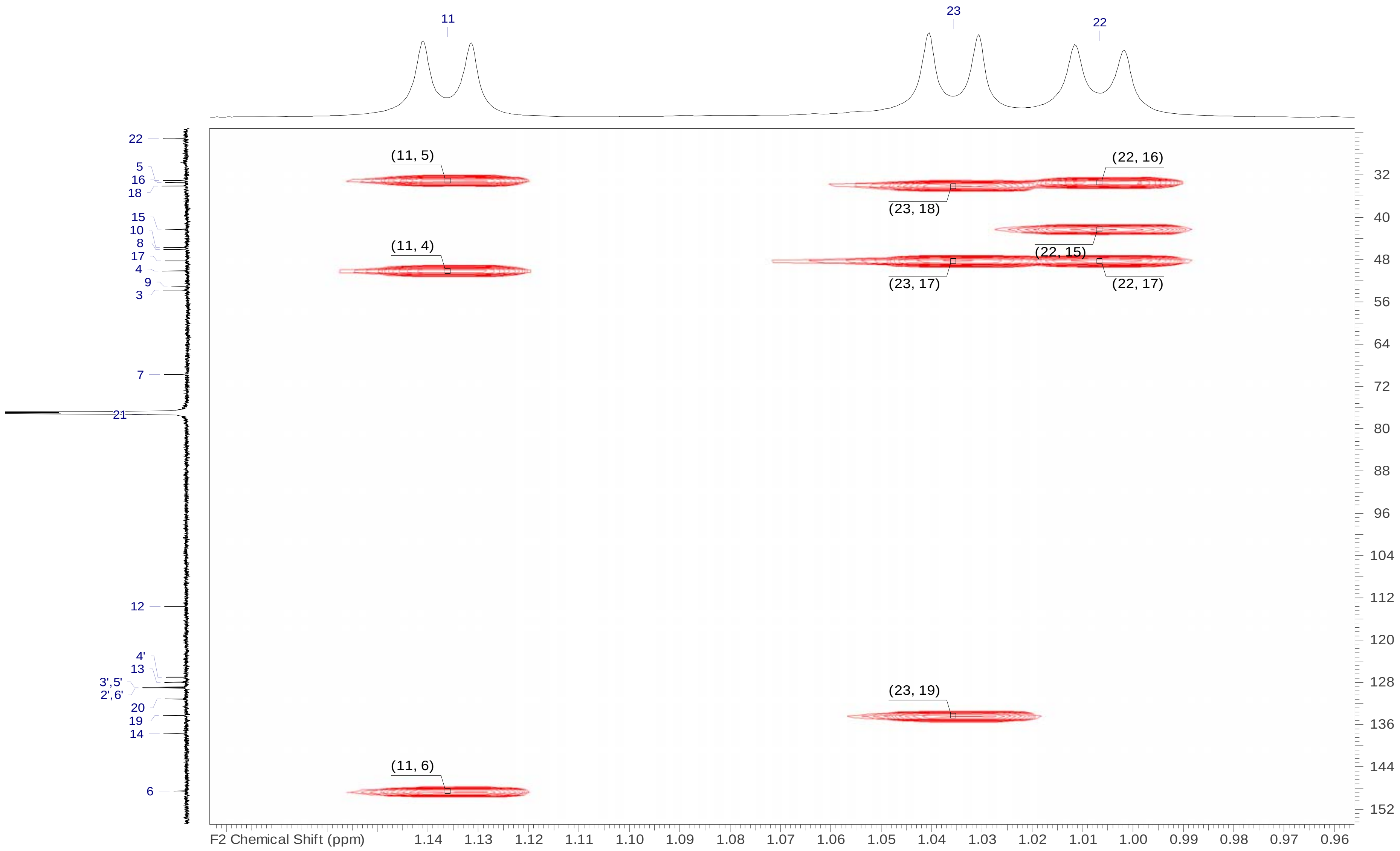

Figure S15. Detail of the HMBC NMR spectrum (700 MHz, $CDCl_3$) of 21-*O*-Deacetyl-L-696,474 (**8**).

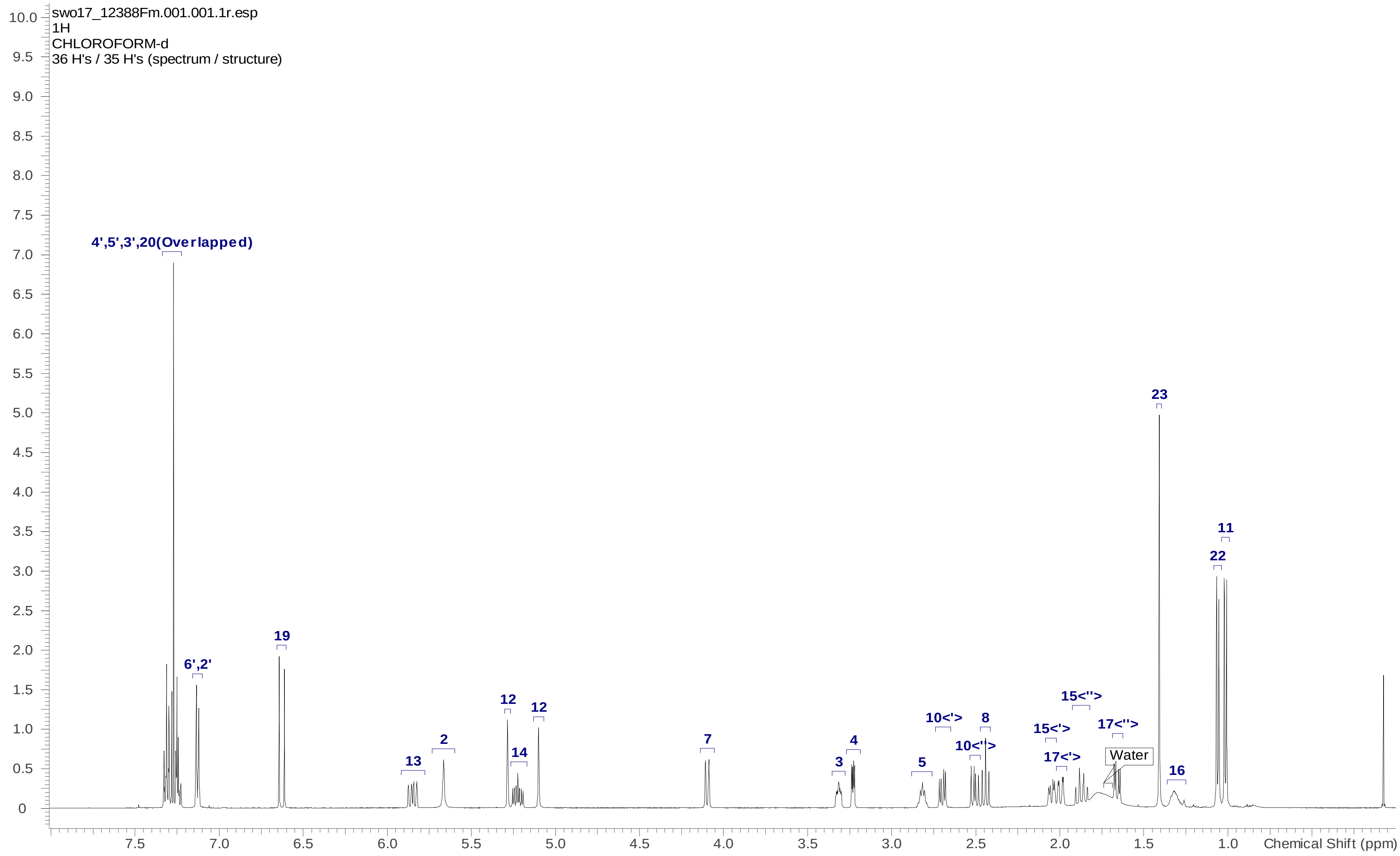

Figure S16. ^{1}H NMR spectrum (500 MHz, $CDCl_3$) of compound **10**.

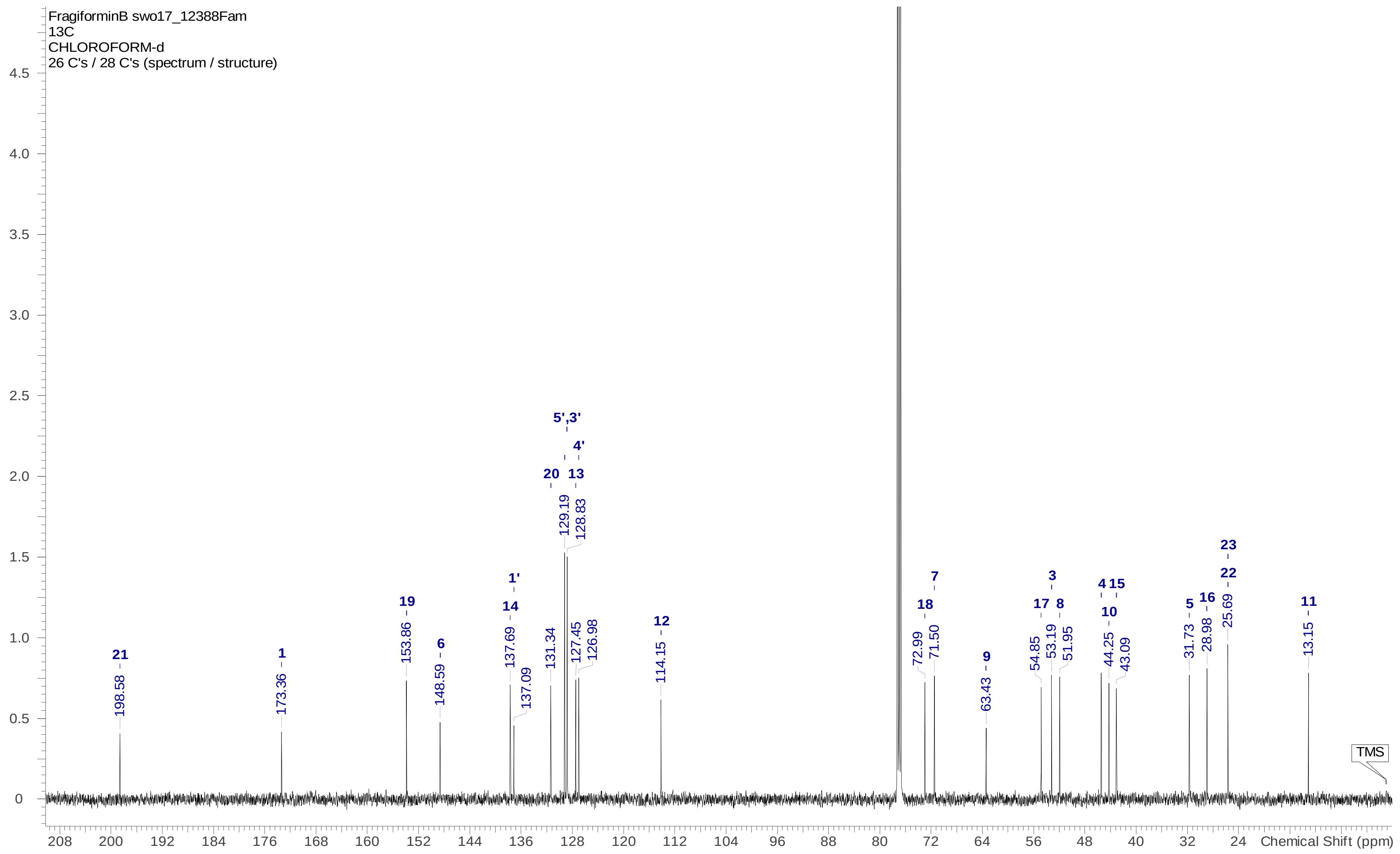

Figure S17. ^{13}C NMR spectrum (125 MHz, $CDCl_3$) of compound **10**.

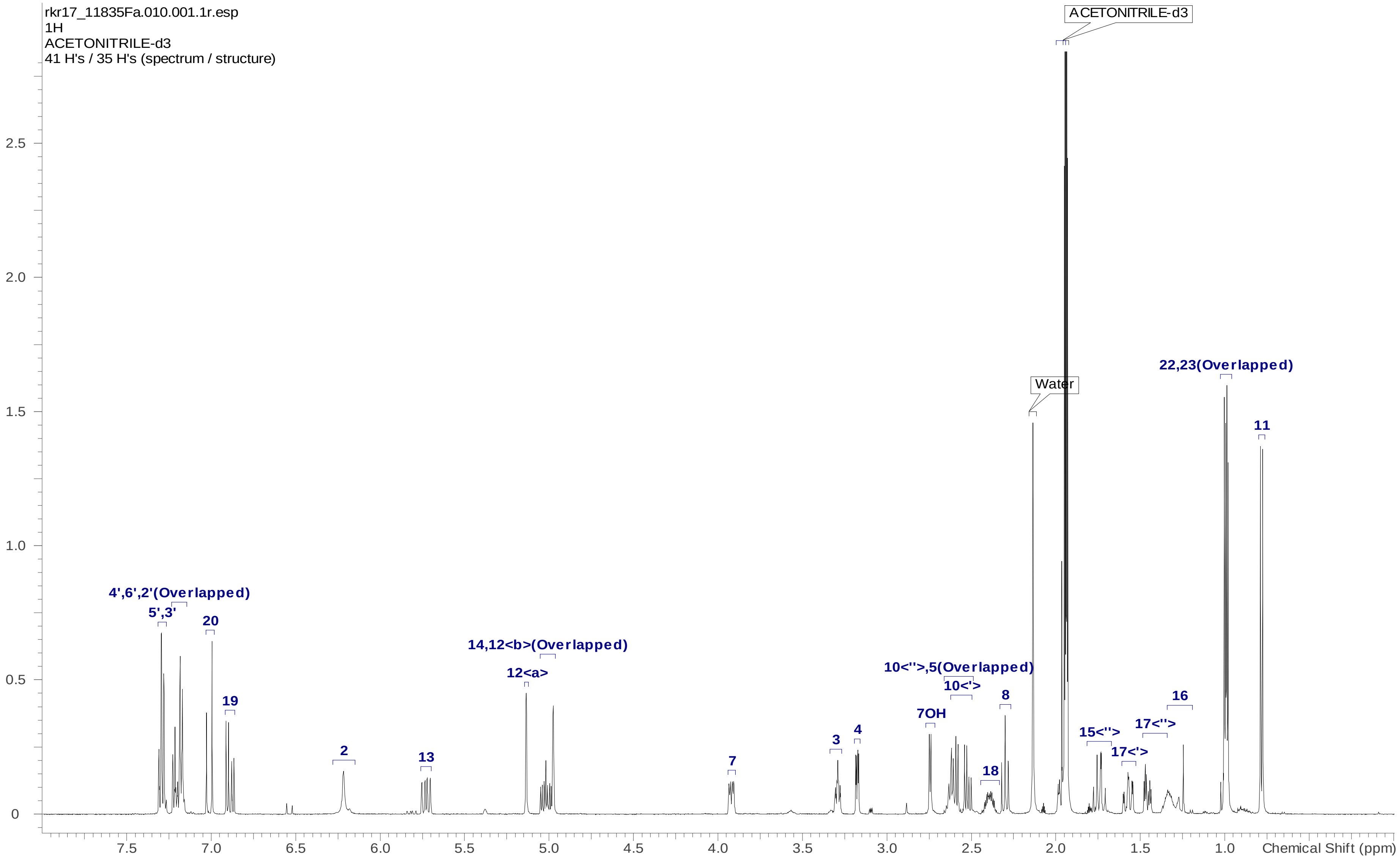

Figure S18. ^{1}H NMR spectrum (500 MHz, CD_3CN) of compound **11**.

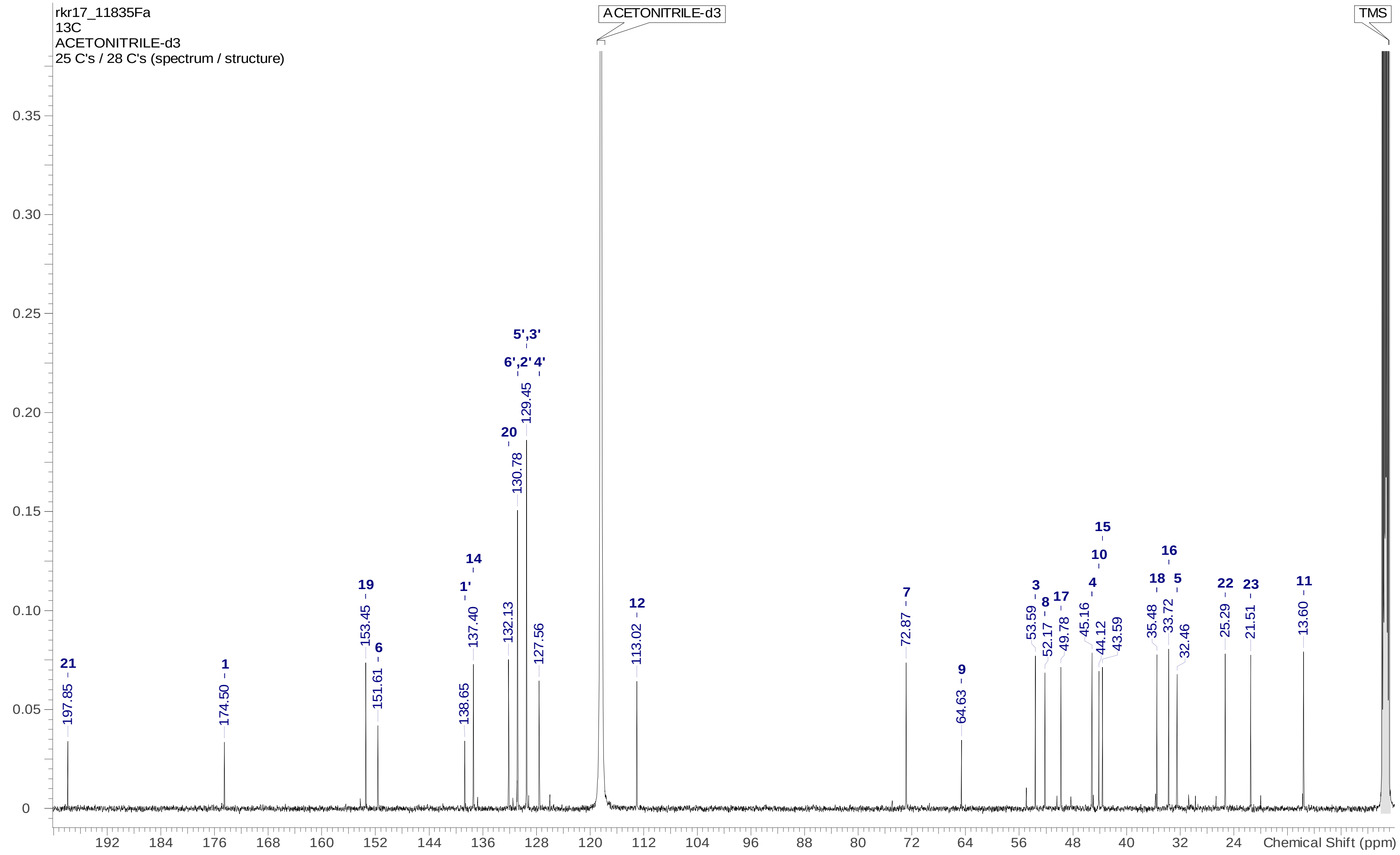

Figure S19. ^{13}C NMR spectrum (125 MHz, CD_3CN) of compound **11**.

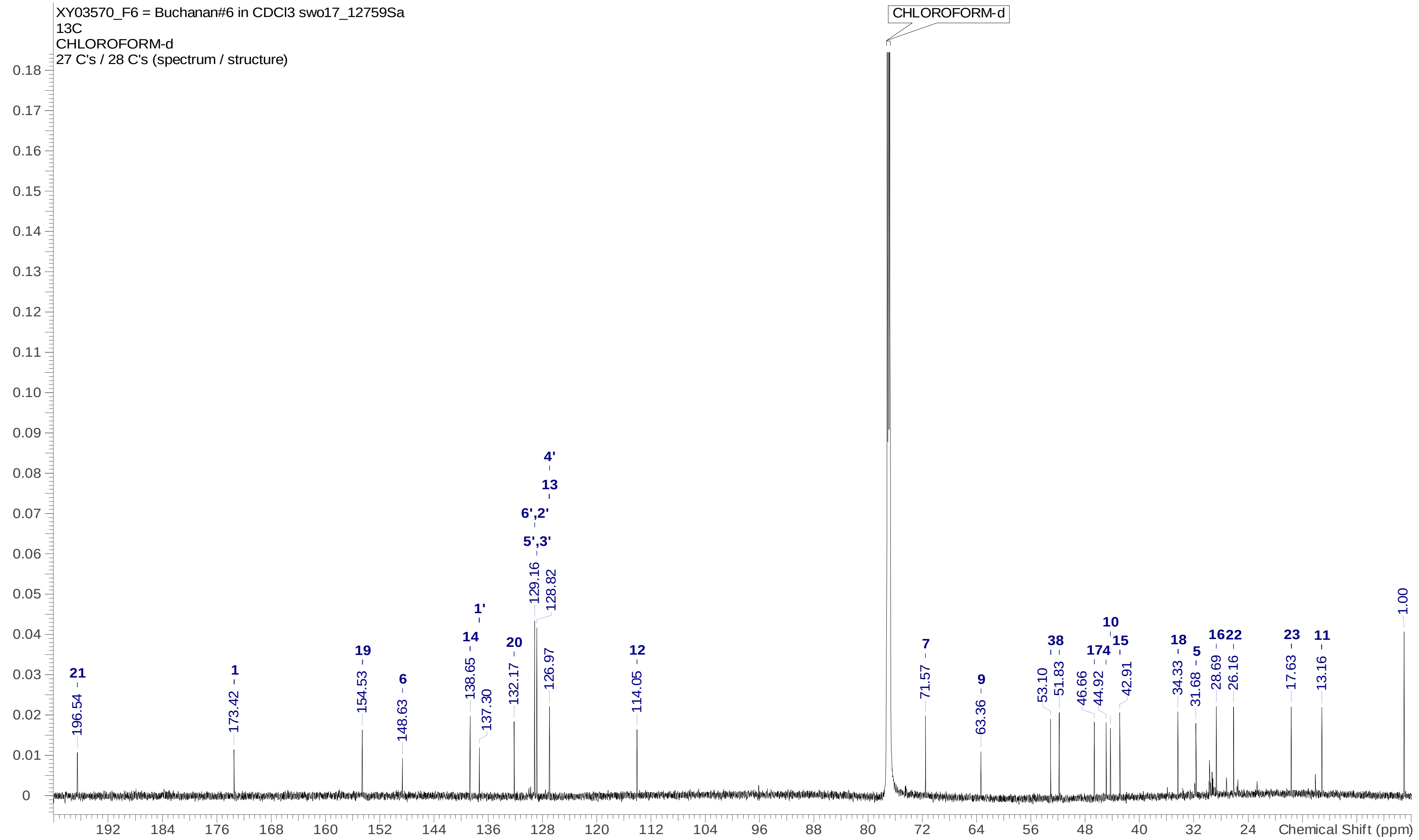

Figure S20. ^{13}C NMR spectrum (175 MHz, $CDCl_3$) of compound **11**.

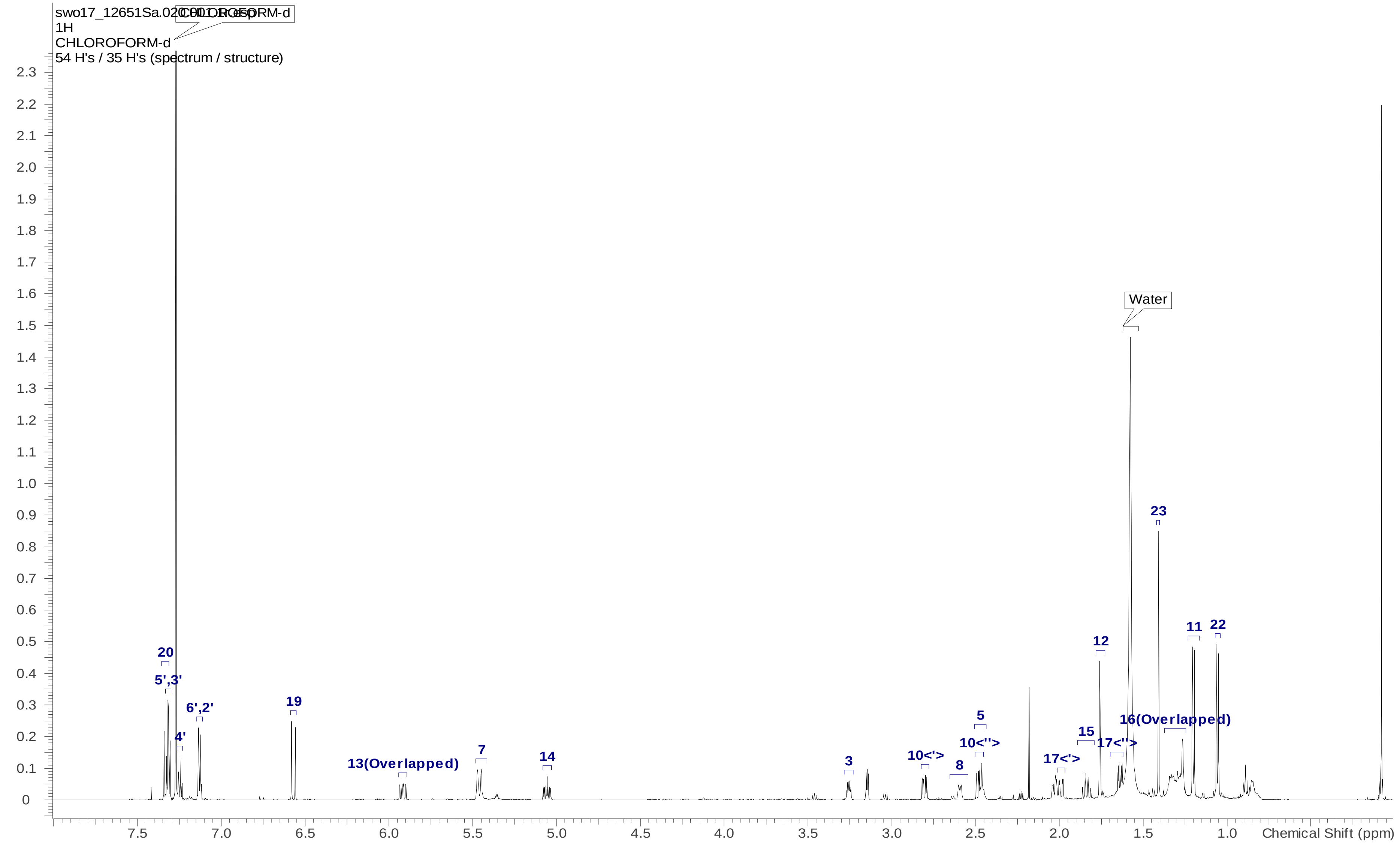

Figure S21. ^{1}H NMR spectrum (700 MHz, $CDCl_3$) of compound **12**.

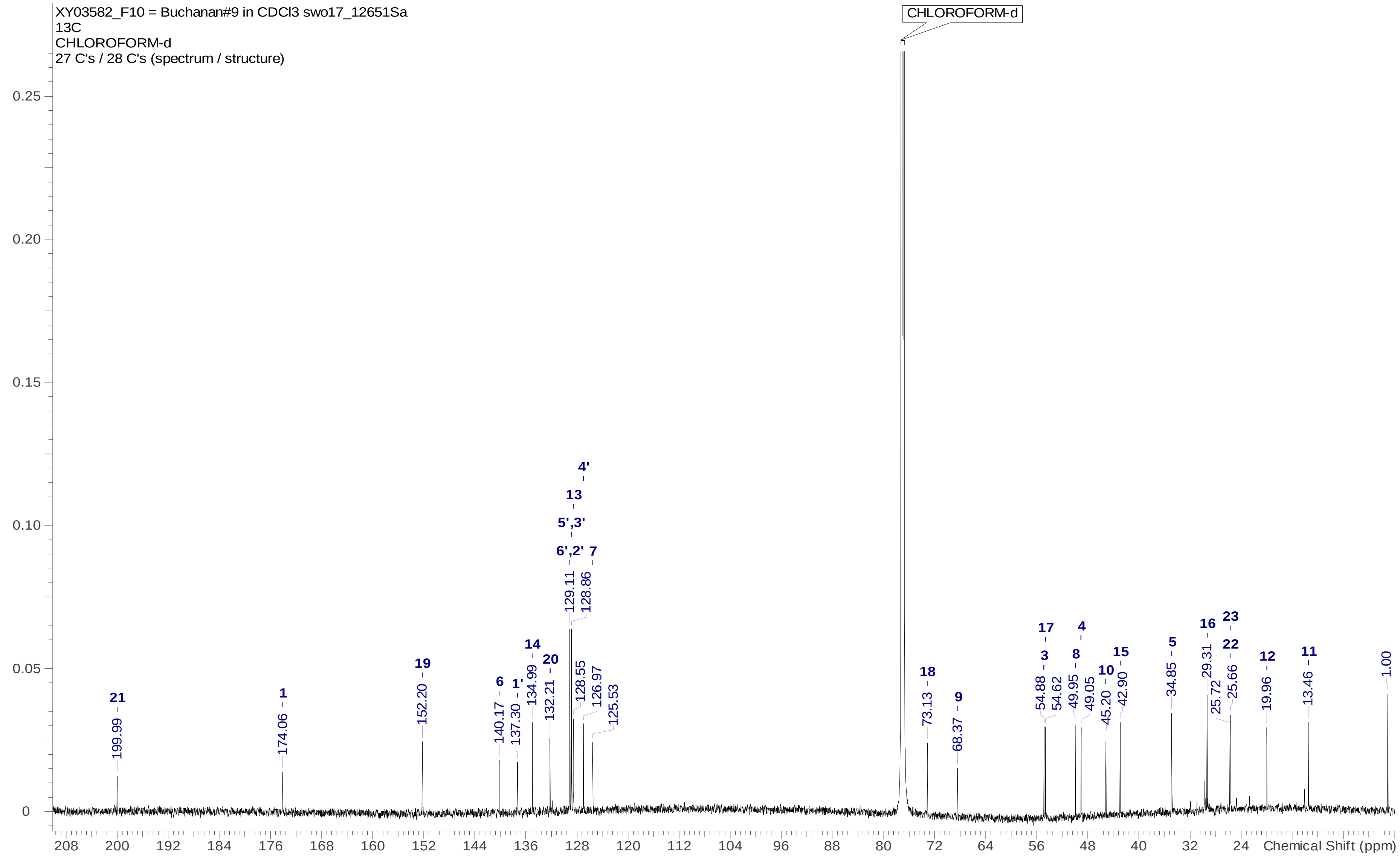

Figure S22. ^{13}C NMR spectrum (175 MHz, $CDCl_3$) of compound **12**.

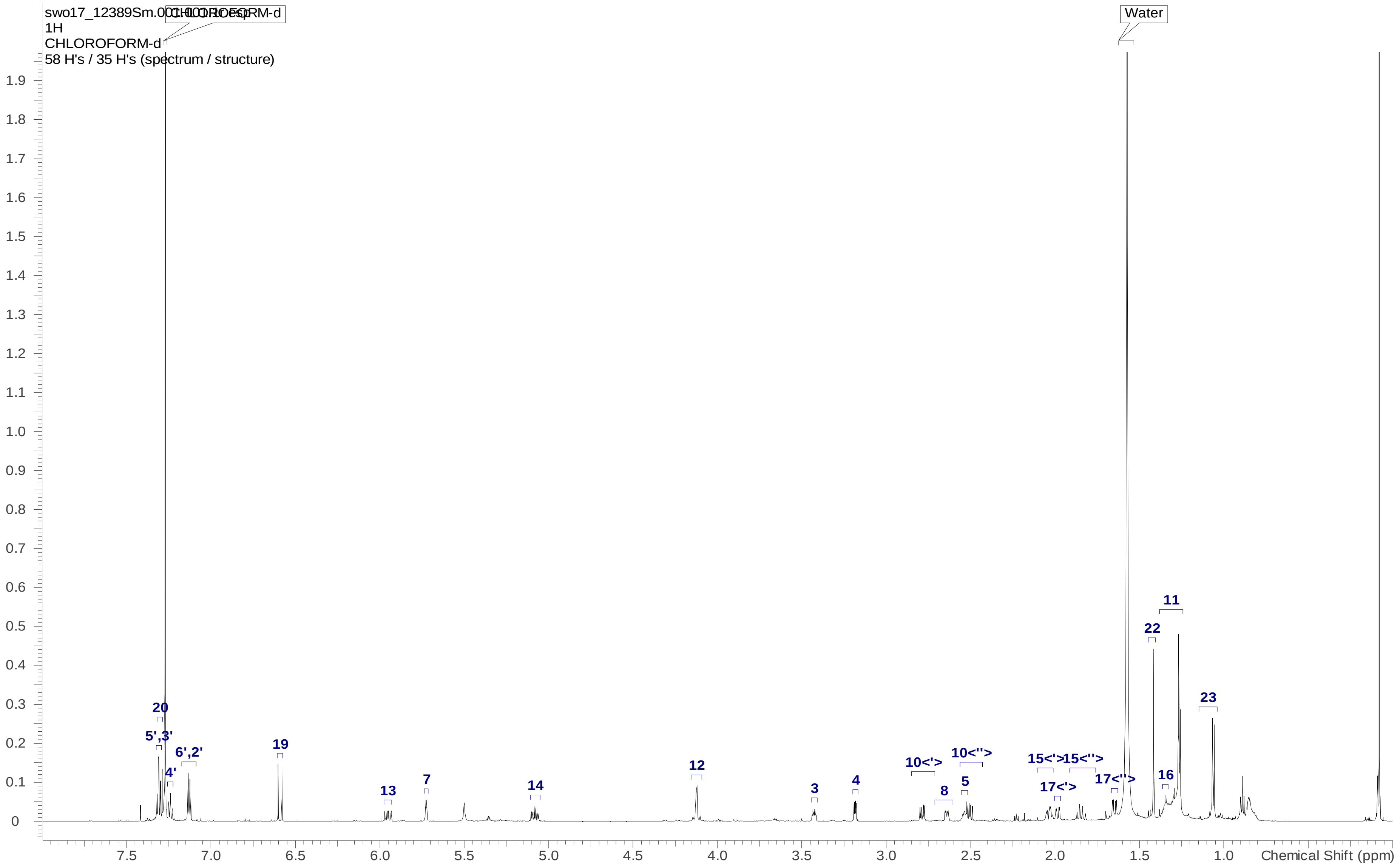

swo17_12389Sm.001.1r.esp
CHLOROFORM-d
1H
CHLOROFORM-d
58 H's / 35 H's (spectrum / structure)
Water
20
5',3'
4'
6',2'
19
13
7
14
12
3
4
10<'>
8
5
10<''>
15<'>
15<''>
17<'>
17<''>
16
22
11
23
Chemical Shift (ppm)

Figure S23. ^{1}H NMR spectrum (700 MHz, $CDCl_3$) of compound **13**.

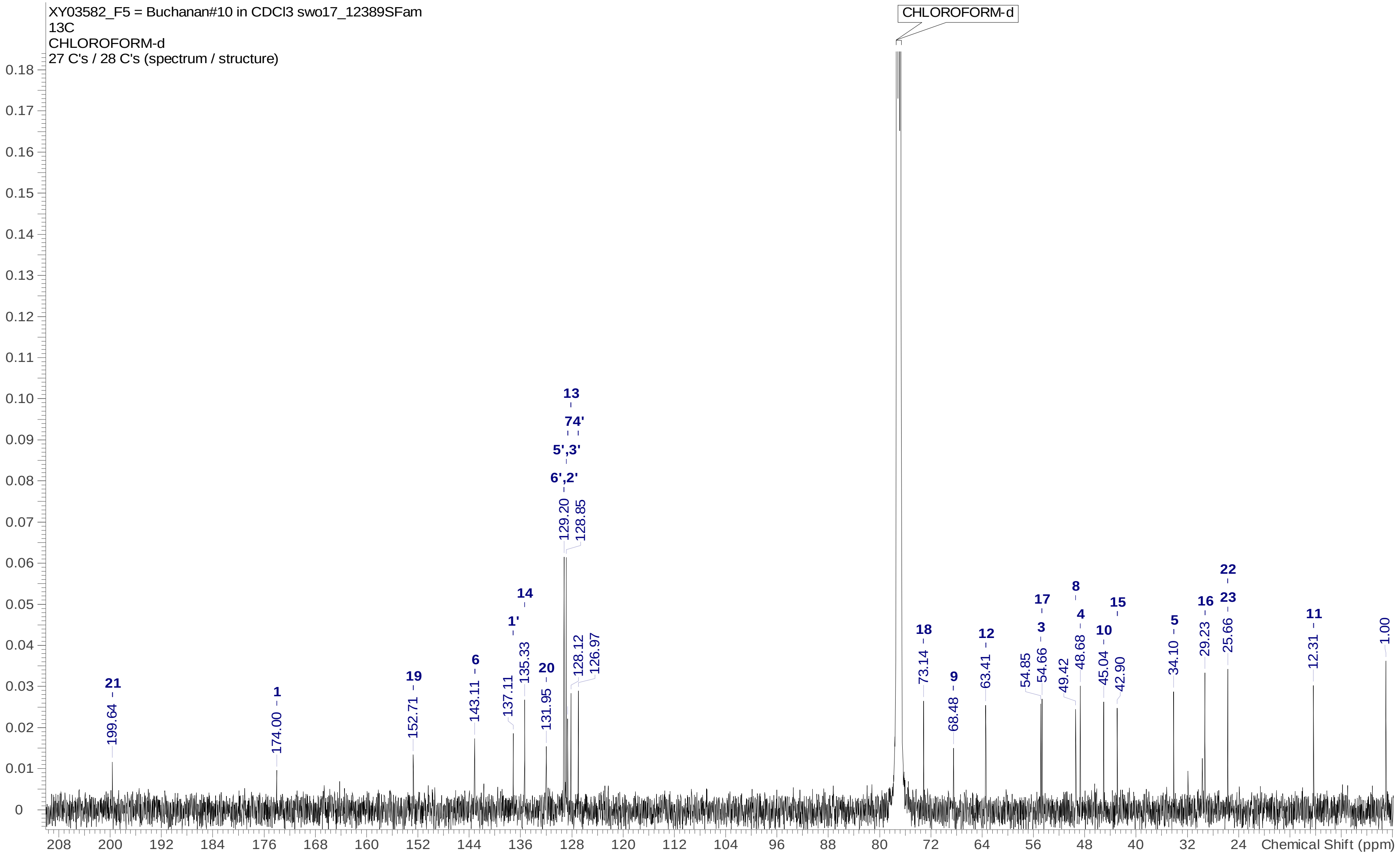

Figure S24. ^{1}H NMR spectrum (125 MHz, $CDCl_3$) of compound **13**.

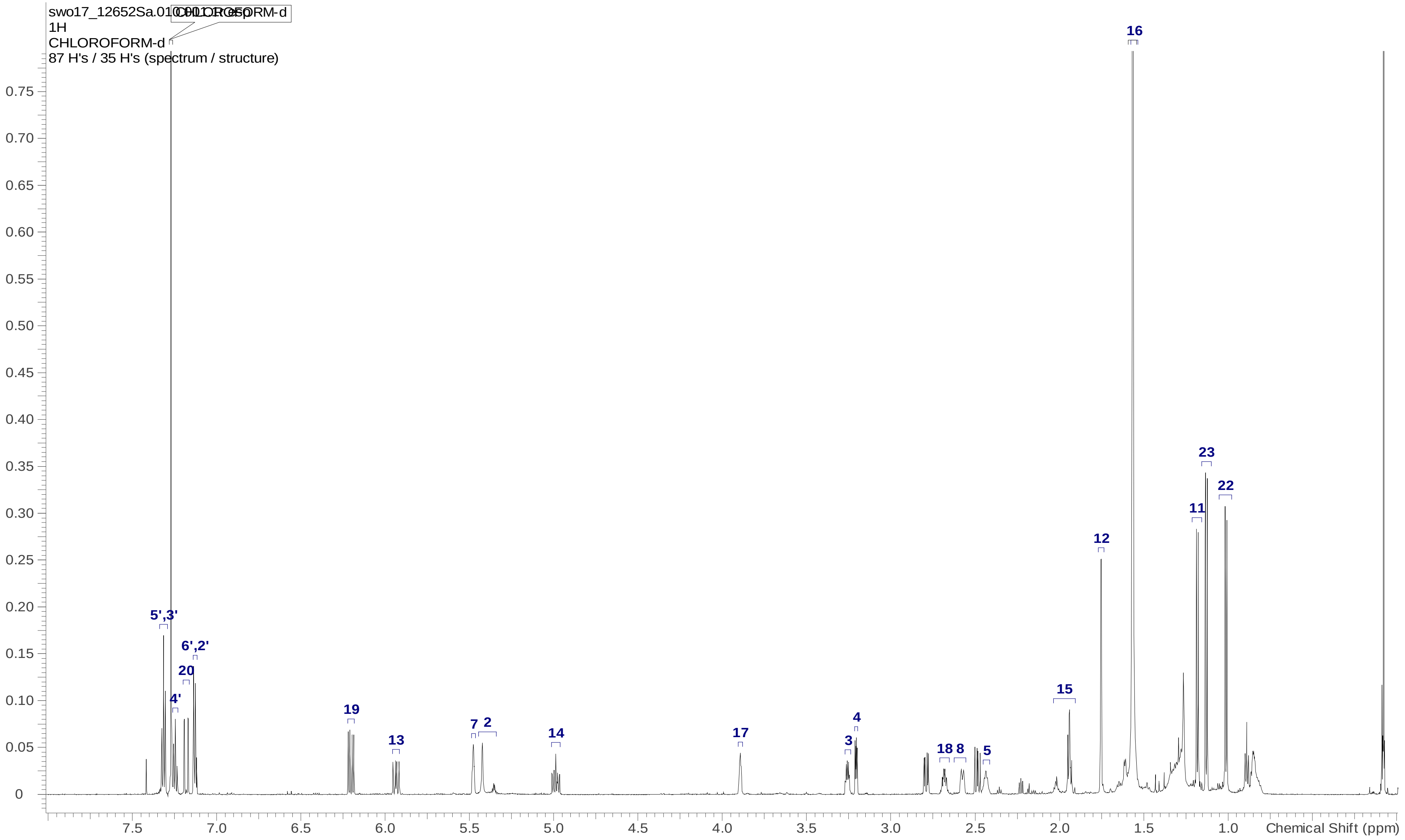

Figure S25. ^{1}H NMR spectrum (700 MHz, $CDCl_3$) of compound **14**.

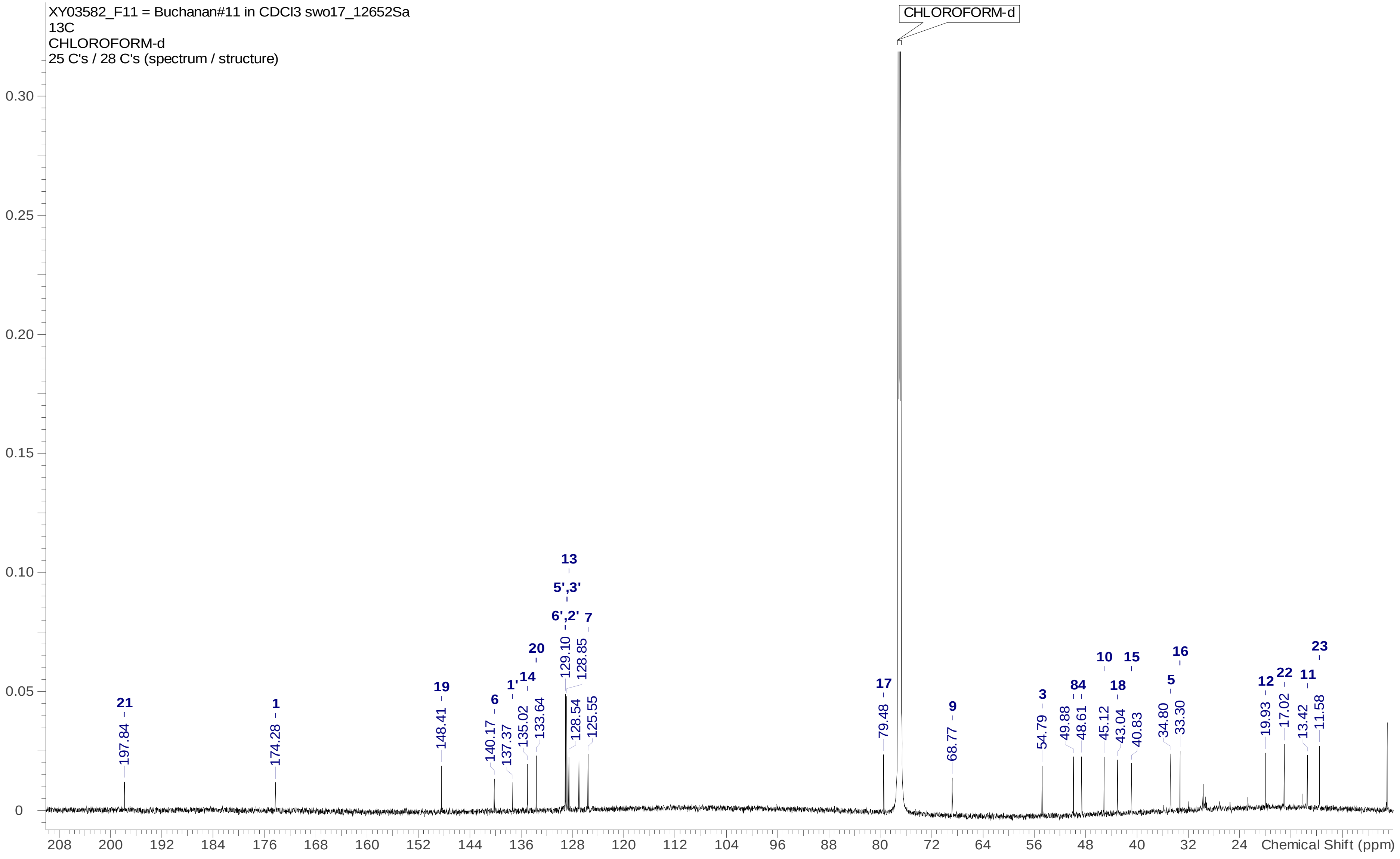

Figure S26. ^{13}C NMR spectrum (175 MHz, $CDCl_3$) of compound **14**.

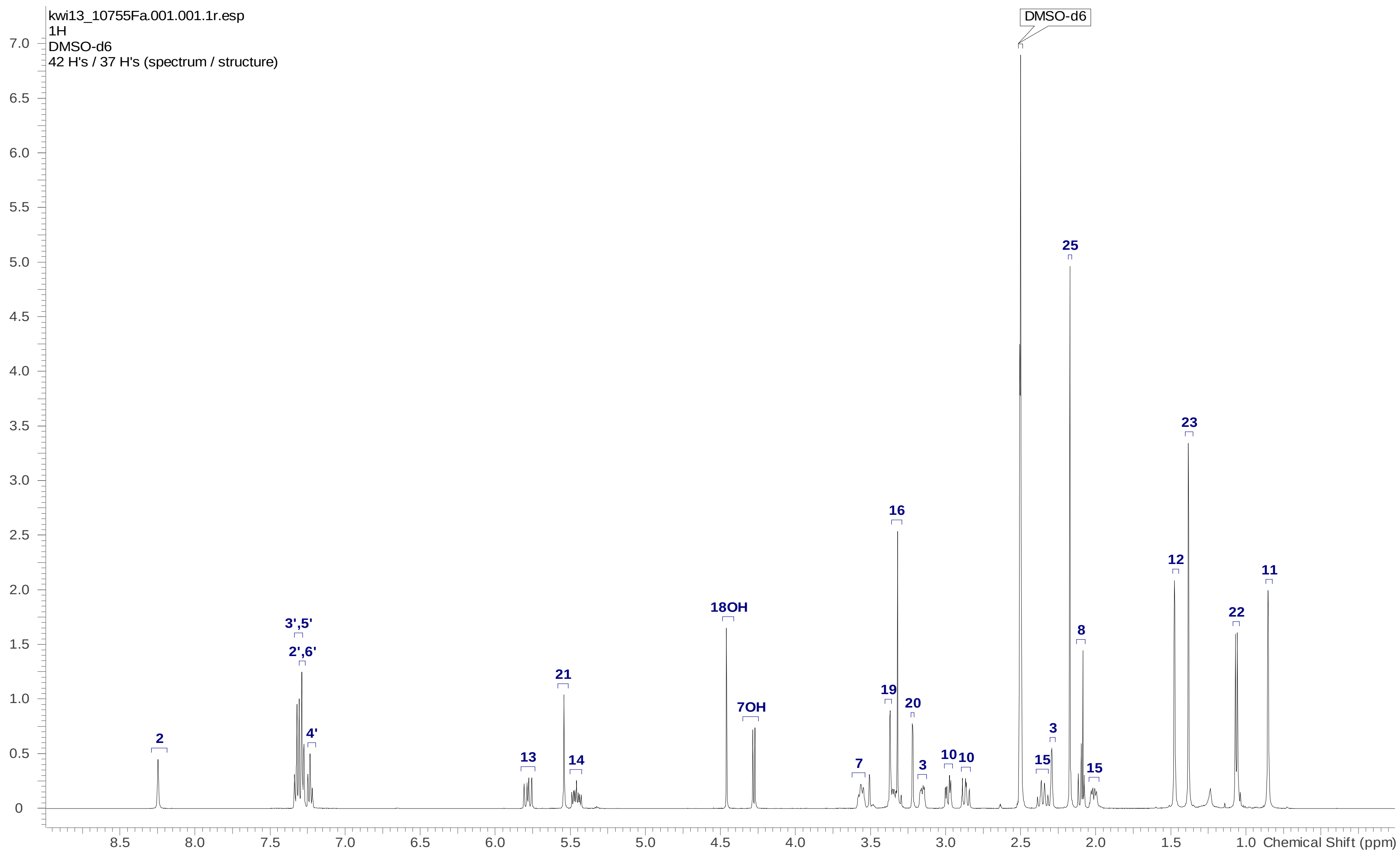

Figure S27. ^{1}H NMR spectrum (500 MHz, DMSO-d_6) of 19,20-epoxycytochalasin C (**15**).

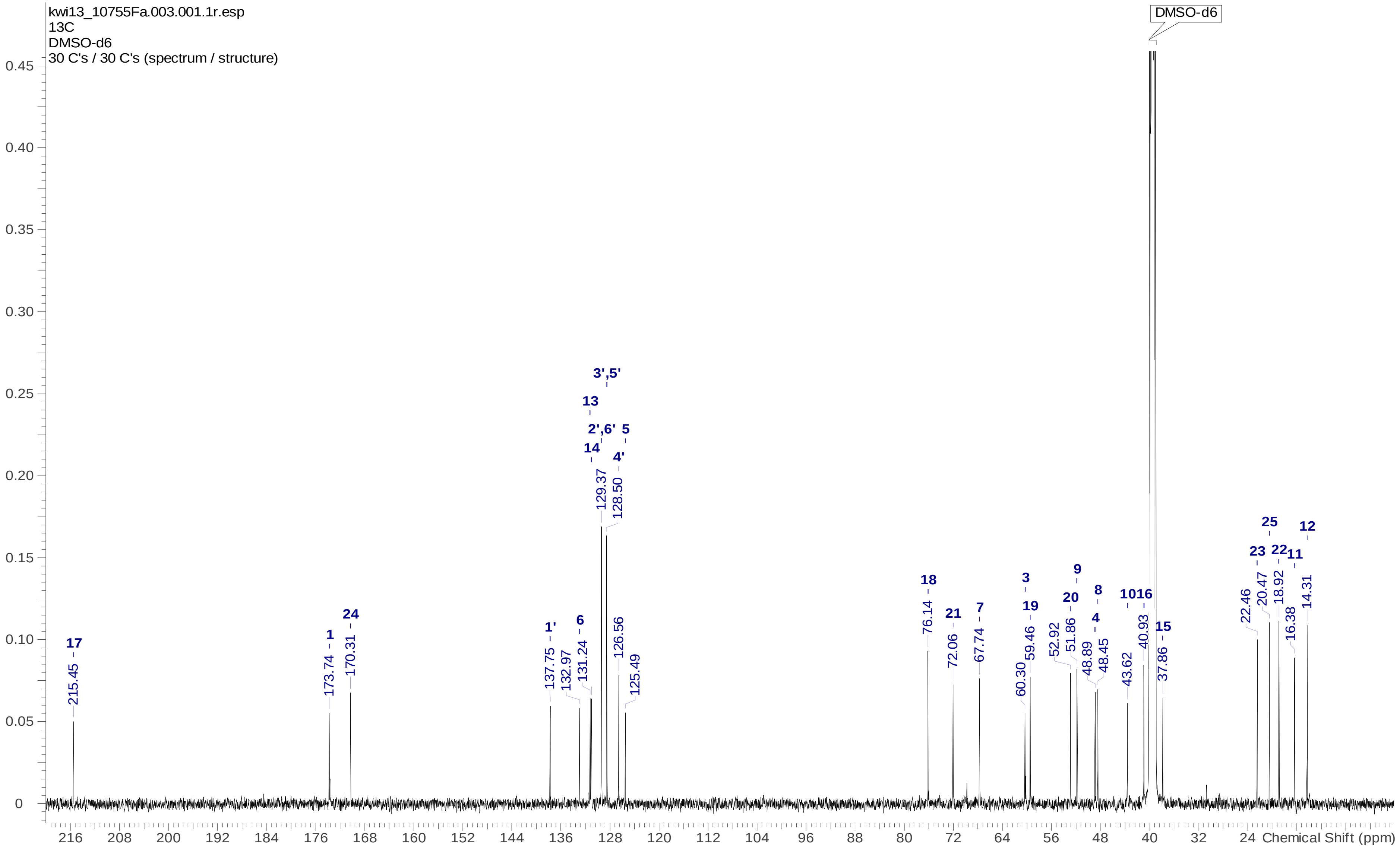

Figure S28. ^{13}C NMR spectrum (125 MHz, DMSO-d_6) of 19,20-epoxycytochalasin C (**15**).

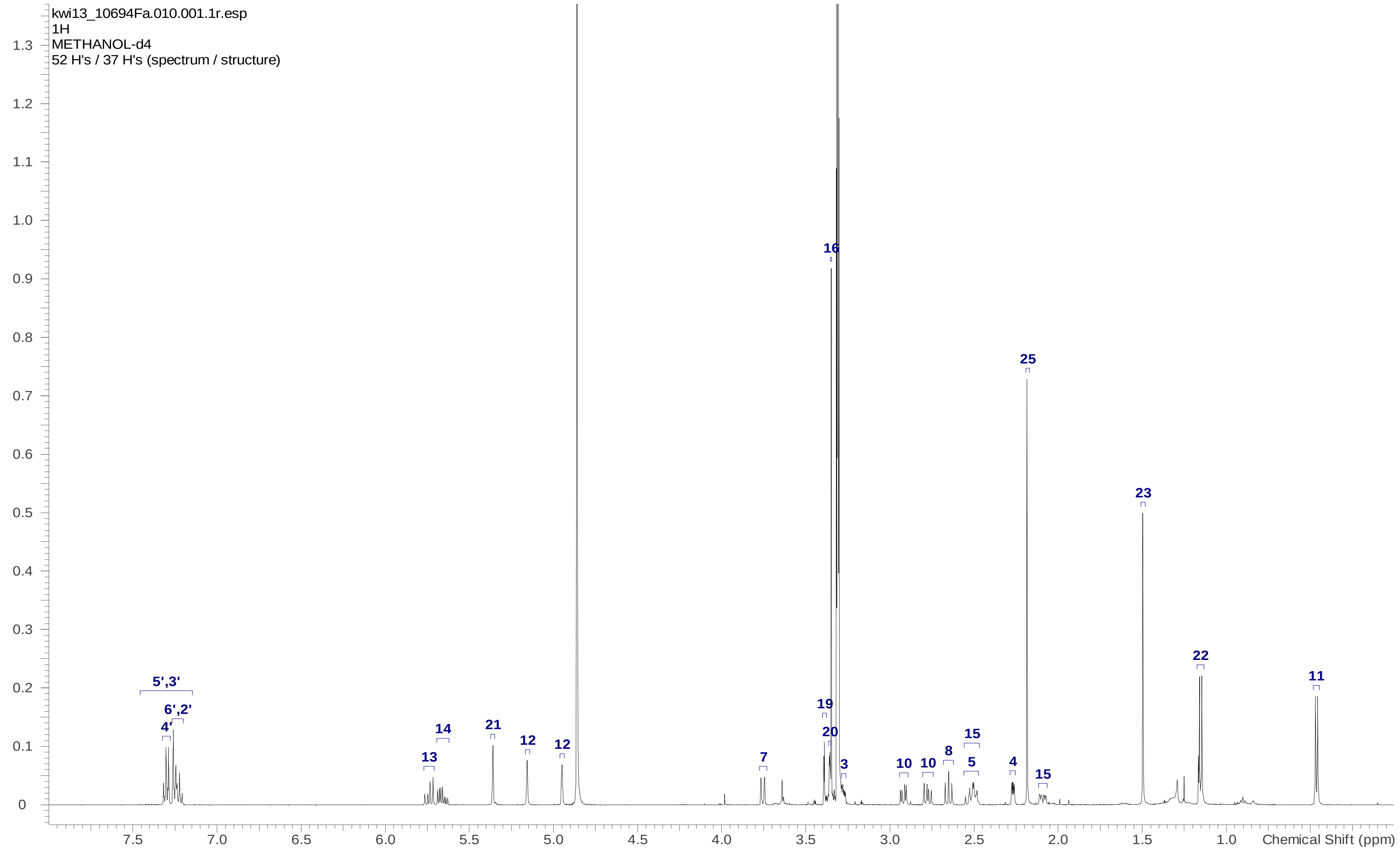

Figure S29. ^{1}H NMR spectrum (500 MHz, CH_3OH-d_4) of 19,20-epoxycytochalasin D (**16**).

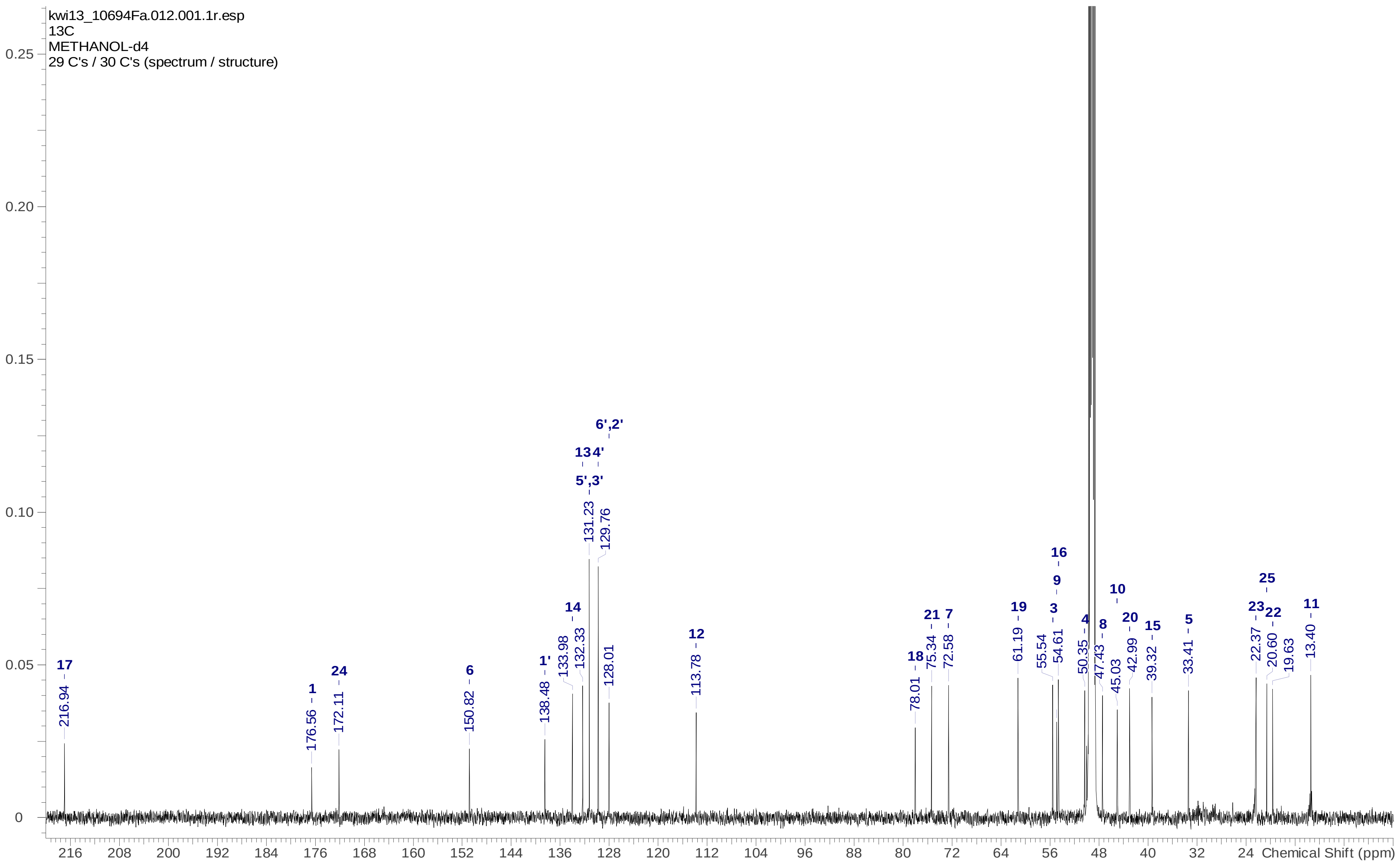

Figure S30. ^{13}C NMR spectrum (125 MHz, CH_3OH-d_4) of 19,20-epoxycytochalasin D (**16**).

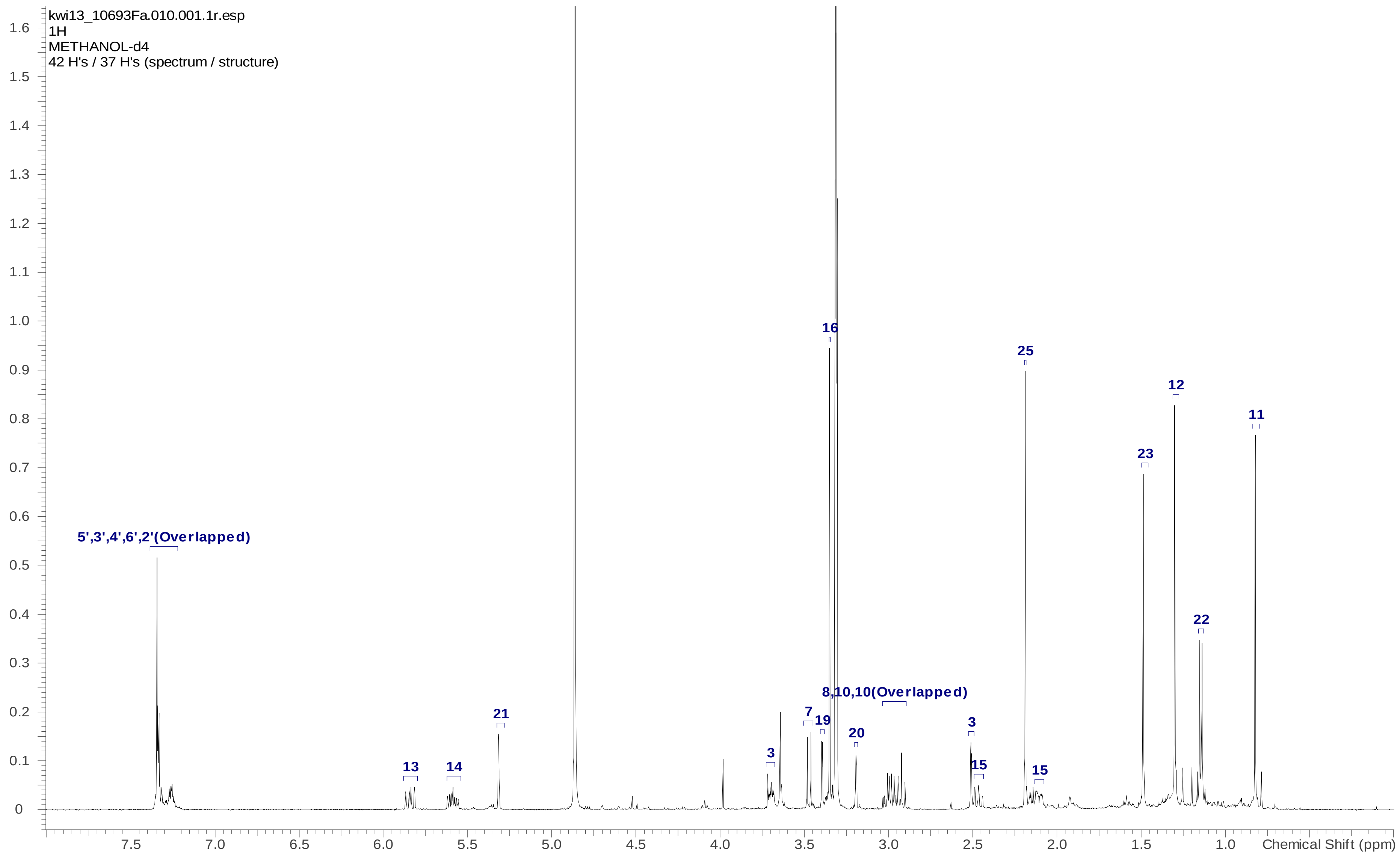

Figure S31. ^{1}H NMR spectrum (500 MHz, CH_3OH-d_4) of 19,20-epoxycytochalasin N (**17**).

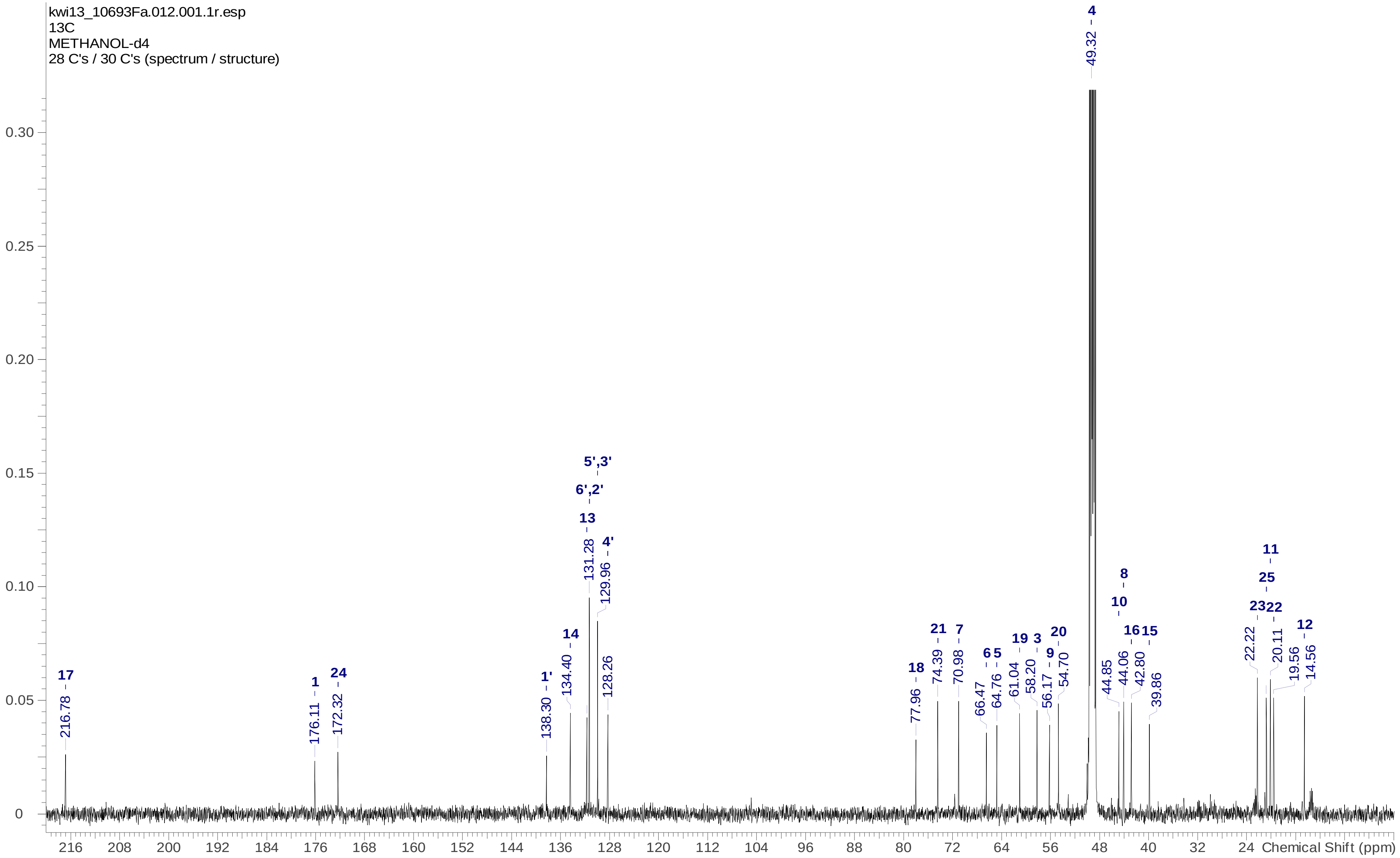

Figure S32. ^{13}C NMR spectrum (125 MHz, CH_3OH-d_4) of 19,20-epoxycytochalasin N (**17**).

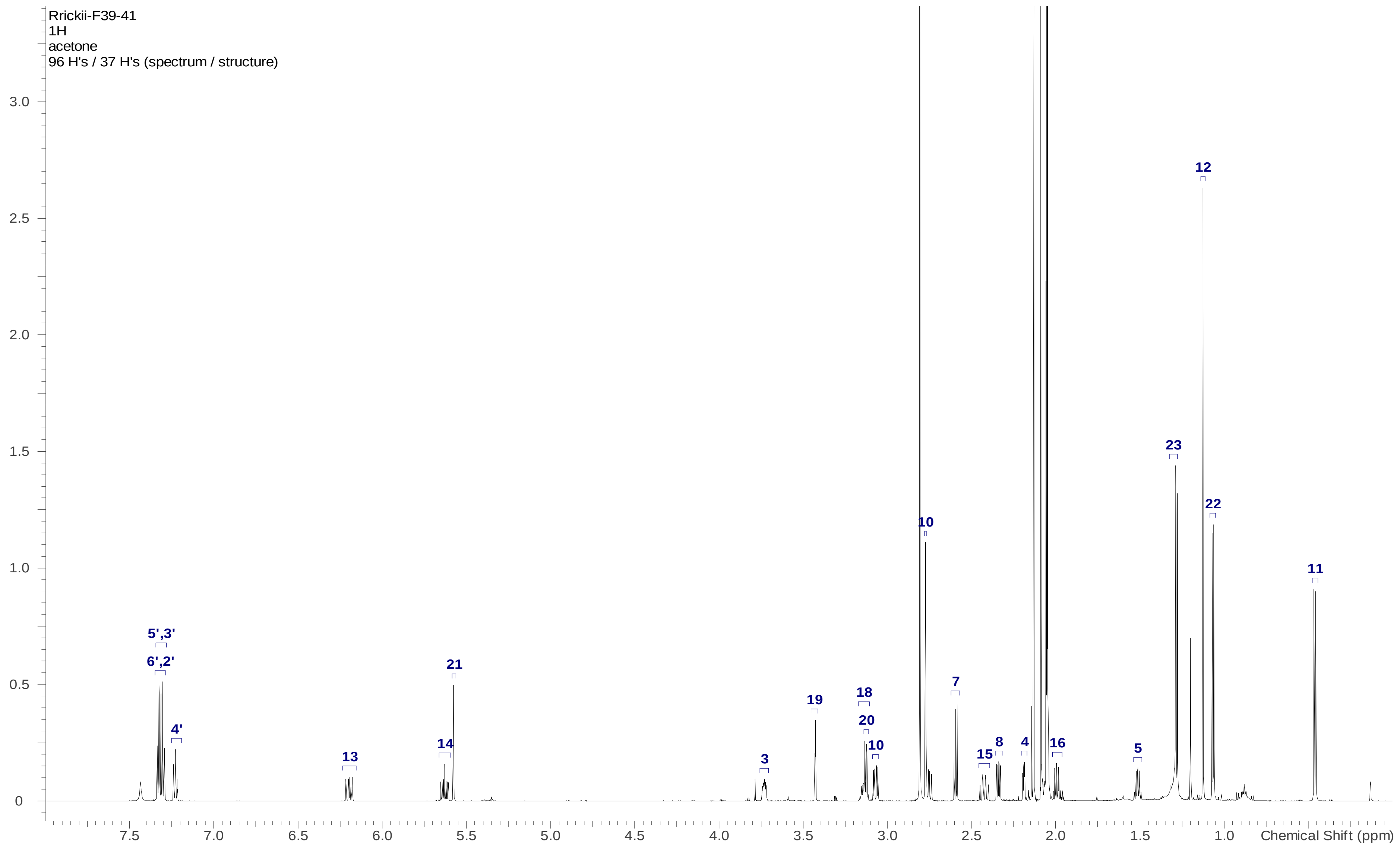

Figure S33. ^{1}H NMR spectrum (500 MHz, acetone-d_6) of 18-deoxy-9,20-epoxycytochalasin Q (**18**).

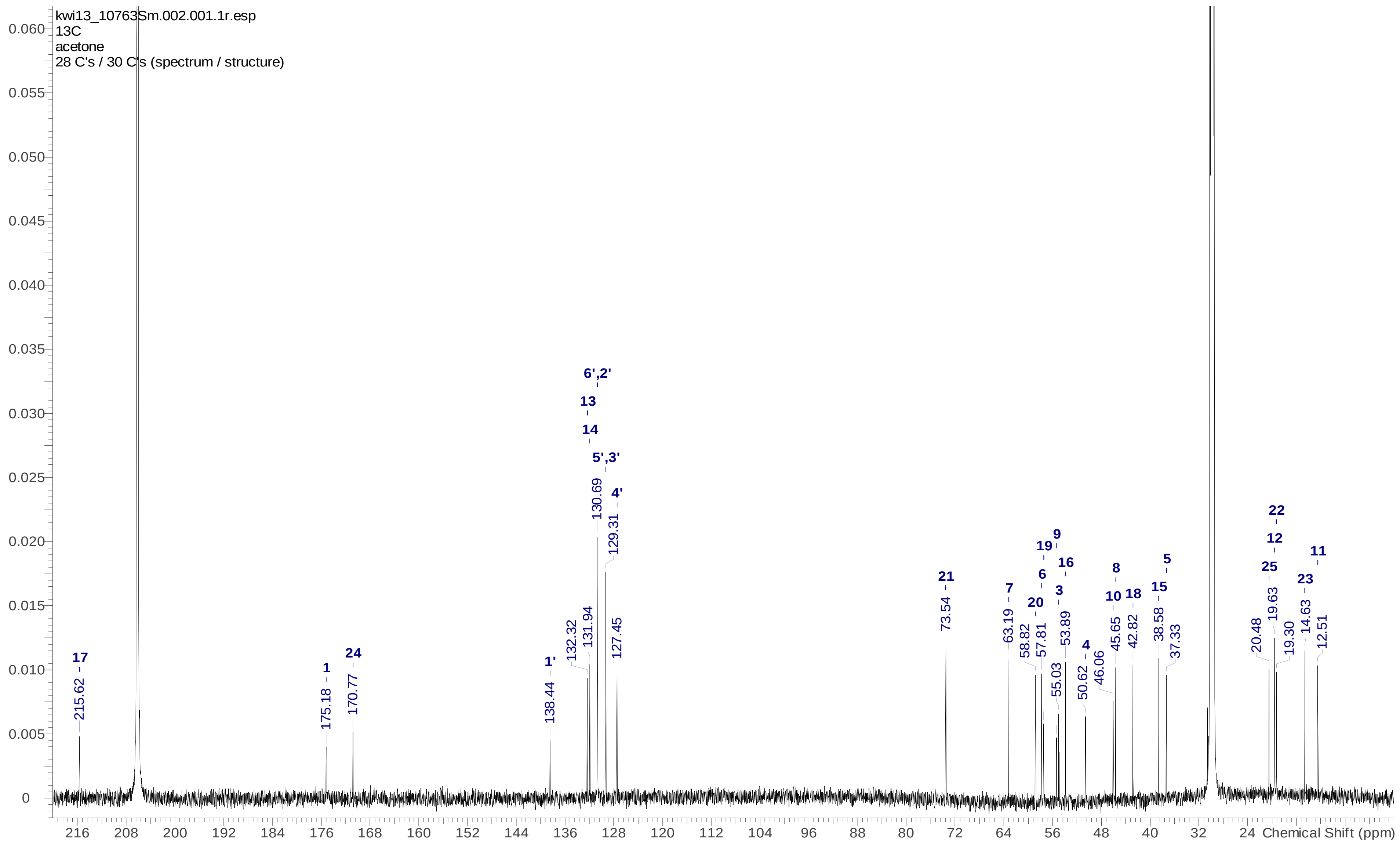

Figure S34. ^{13}C NMR spectrum (175 MHz, acetone-d_6) of 18-deoxy-9,20-epoxycytochalasin Q (**18**).

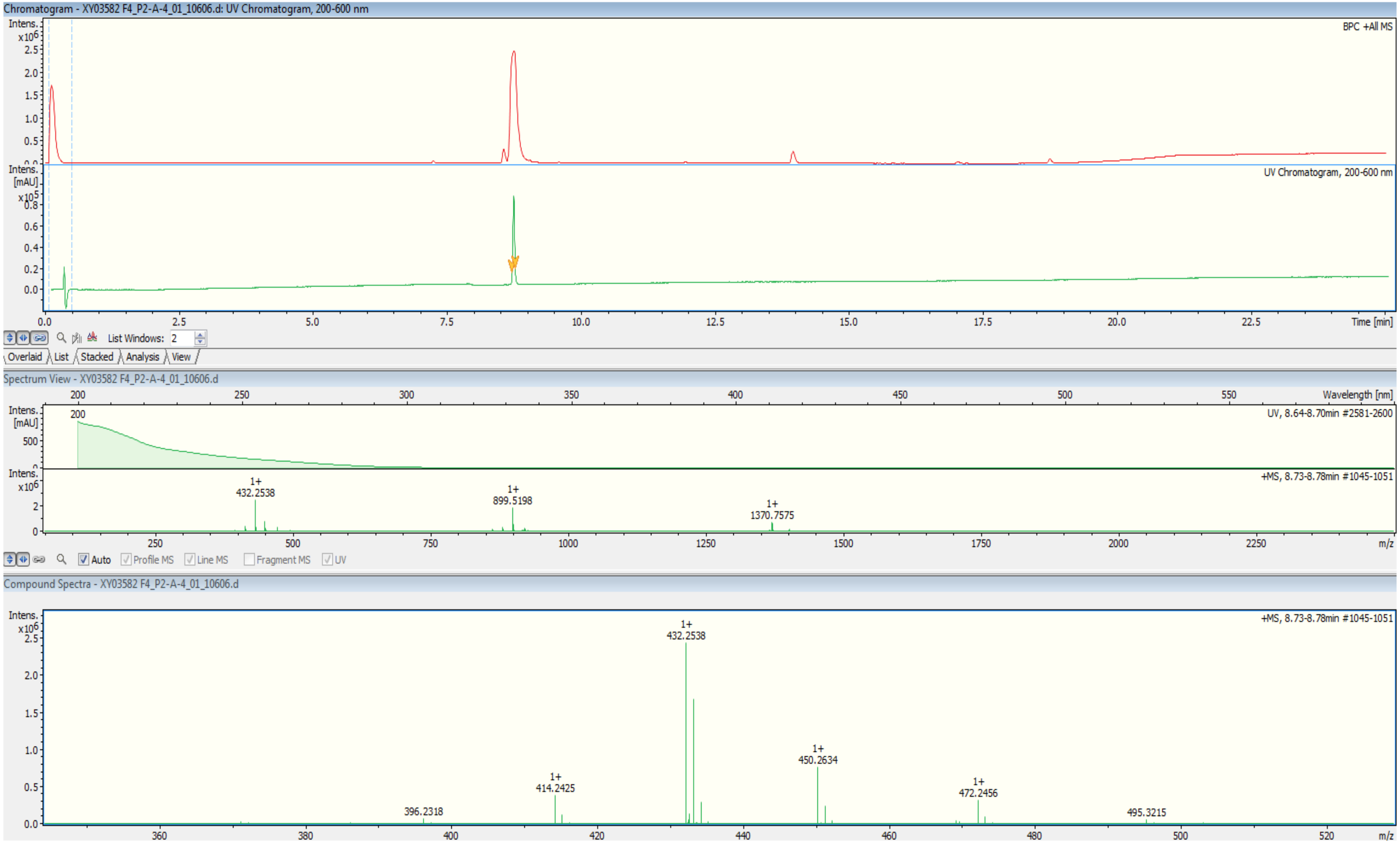

Figure S35. HPLC-HRESIMS data of phenochalasin C (**19**).

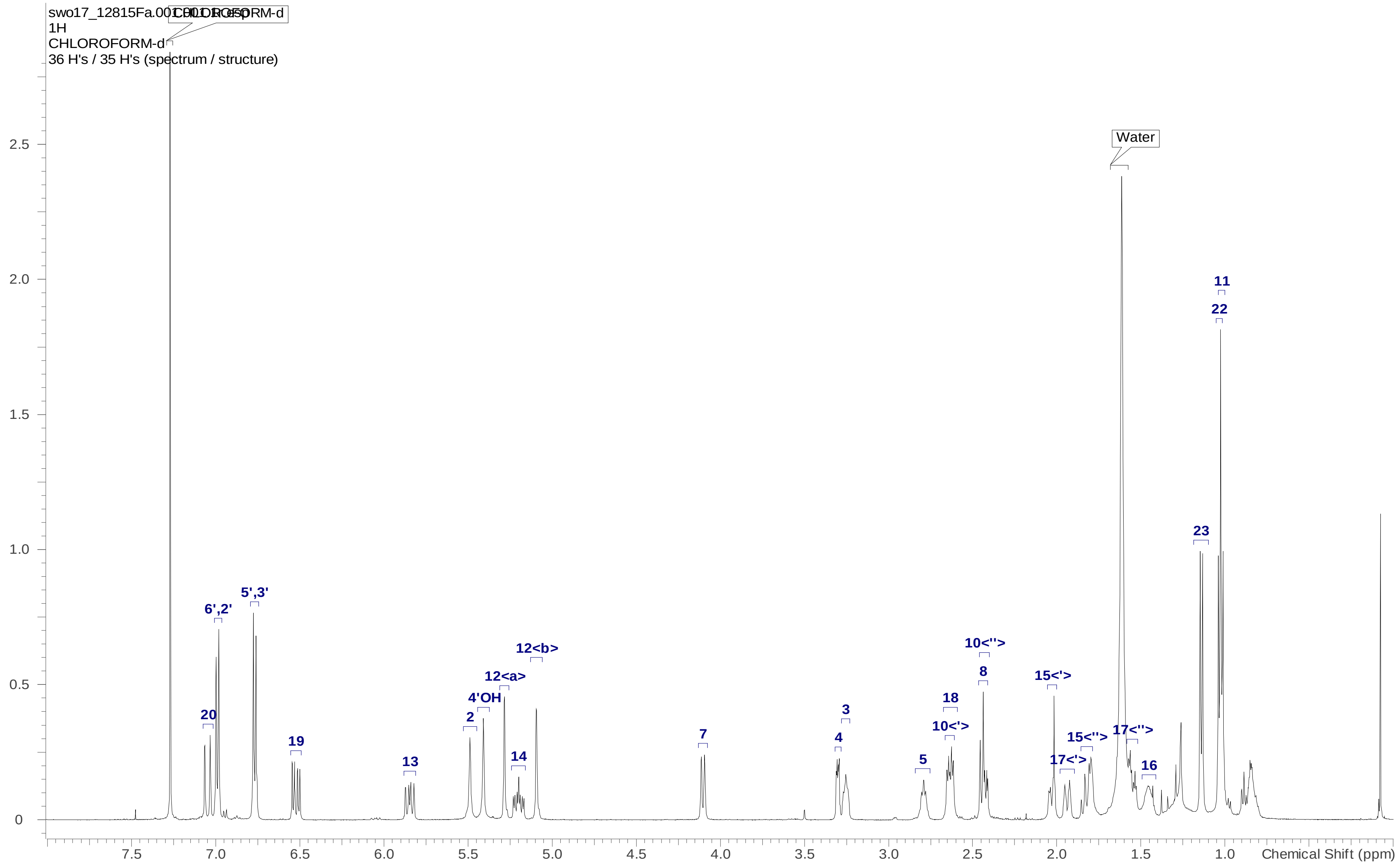

Figure S36. ^{1}H NMR spectrum (500 MHz, $CDCl_3$) of phenochalasin C (**19**).

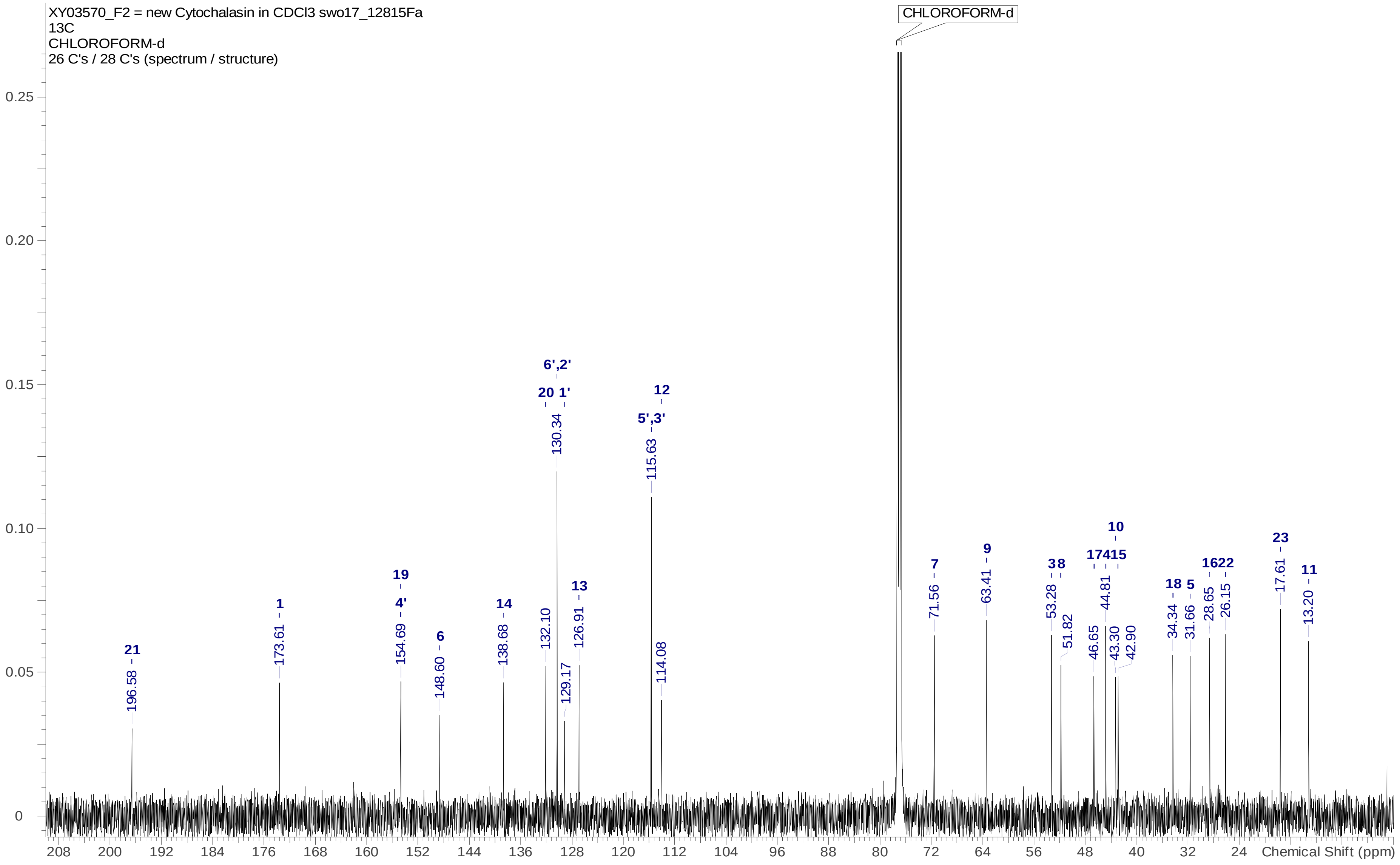

Figure S37. ^{13}C NMR spectrum (125 MHz, $CDCl_3$) of phenochalasin C (**19**).

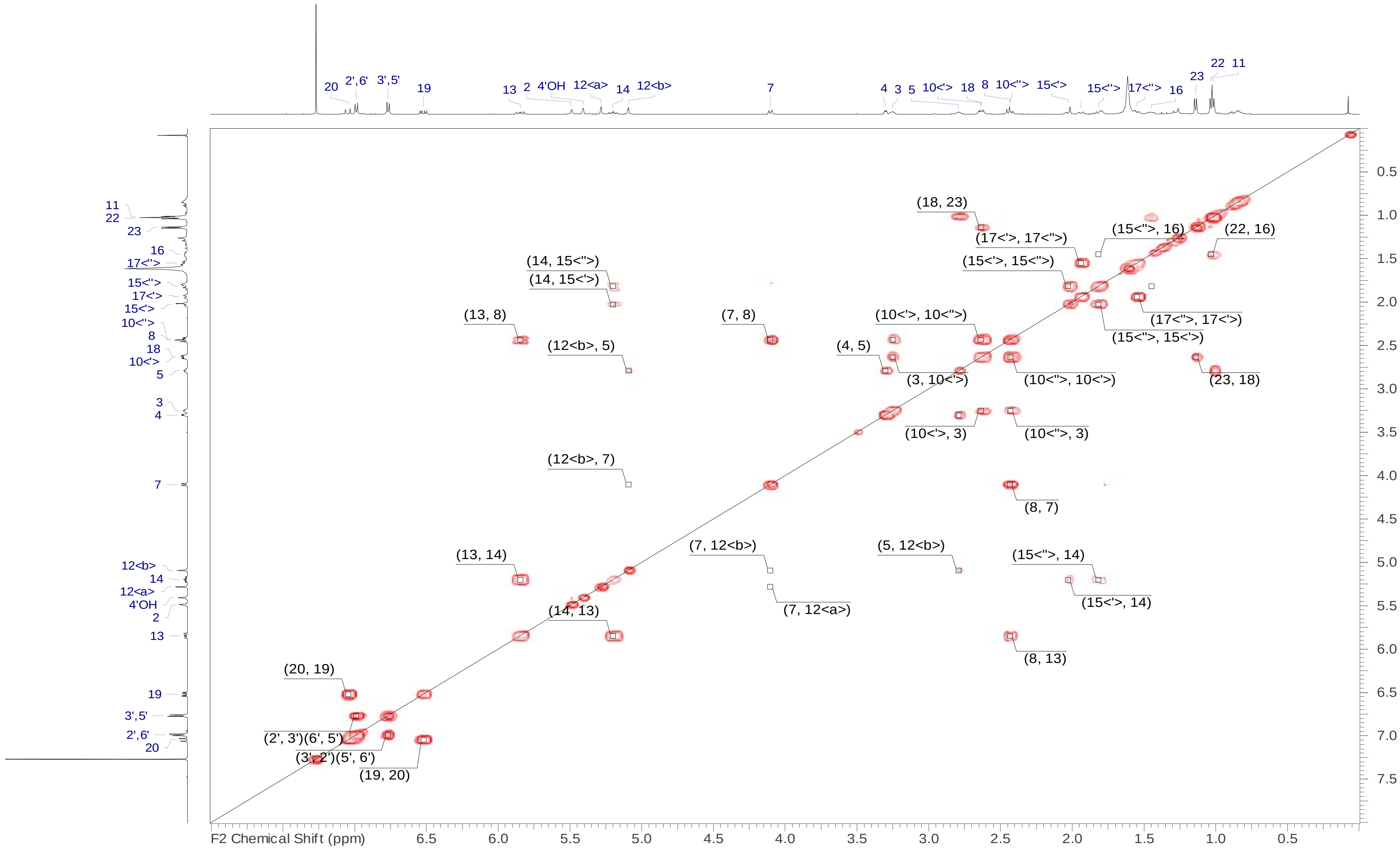

Figure S38. COSY NMR spectrum (500 MHz, $CDCl_3$) of phenochalasin C (**19**).

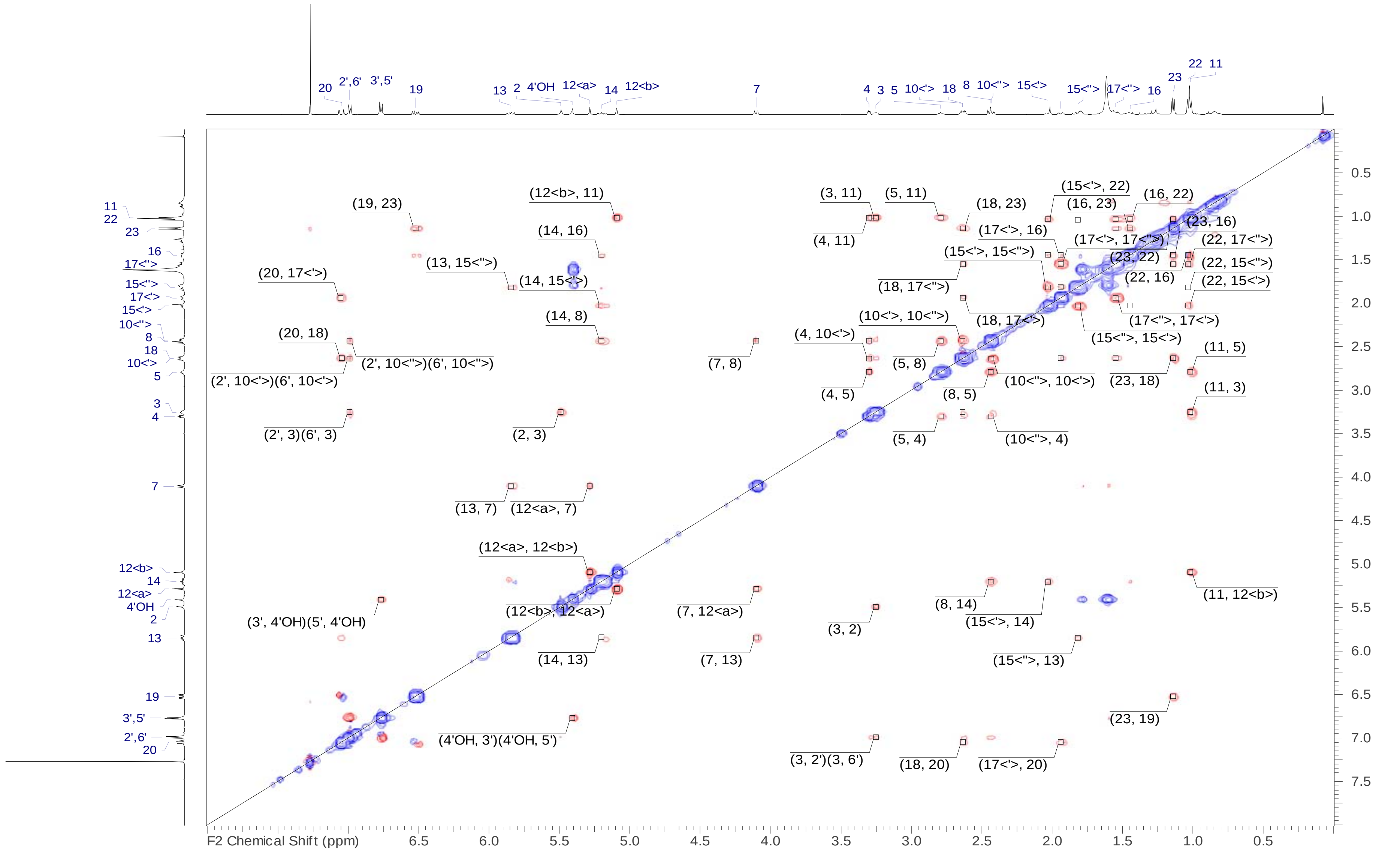

Figure S39. ROESY NMR spectrum (500 MHz, $CDCl_3$) of phenochalasin C (**19**).

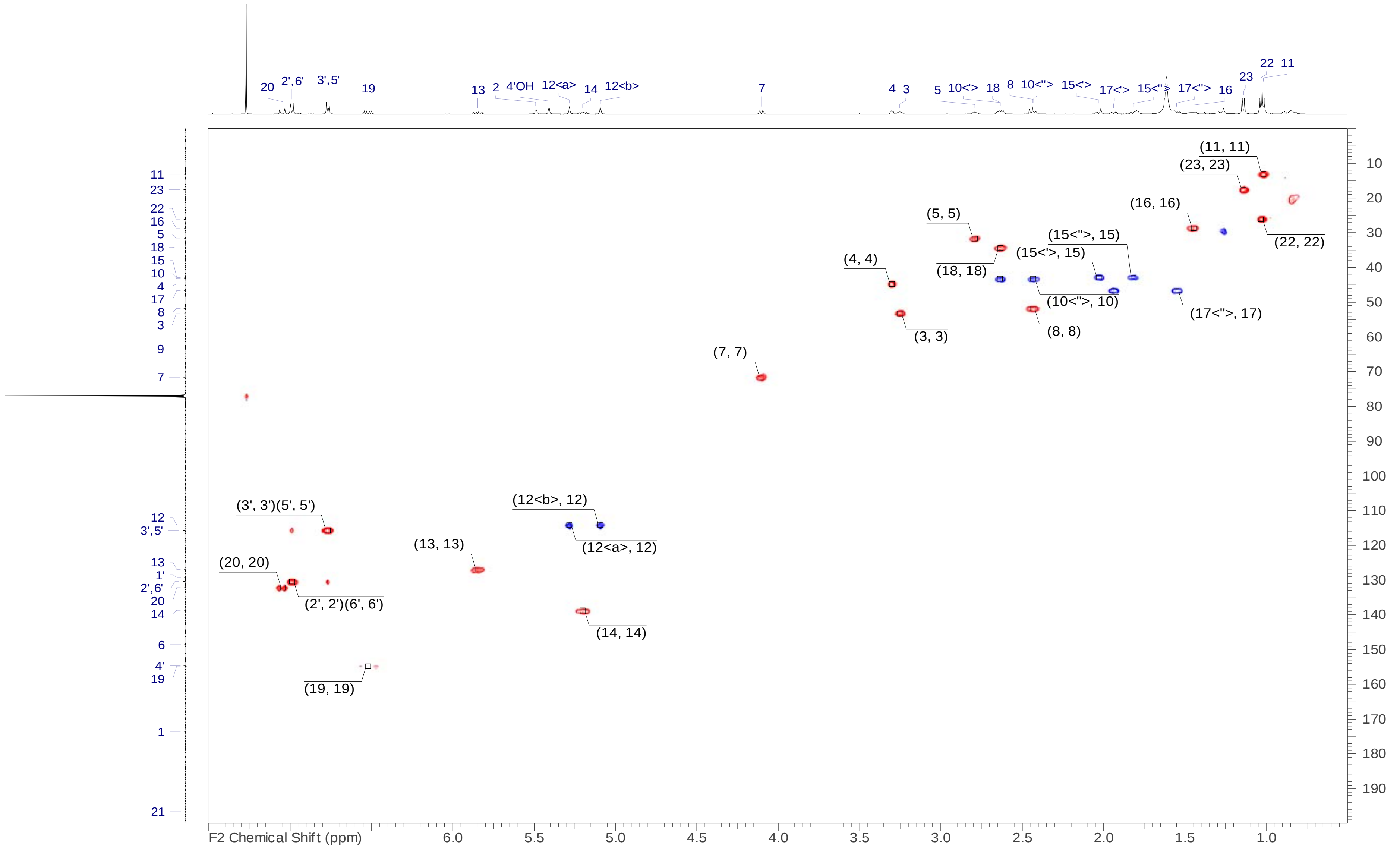

Figure S40. HSQC NMR spectrum (500 MHz, $CDCl_3$) of phenochalasin C (**19**).

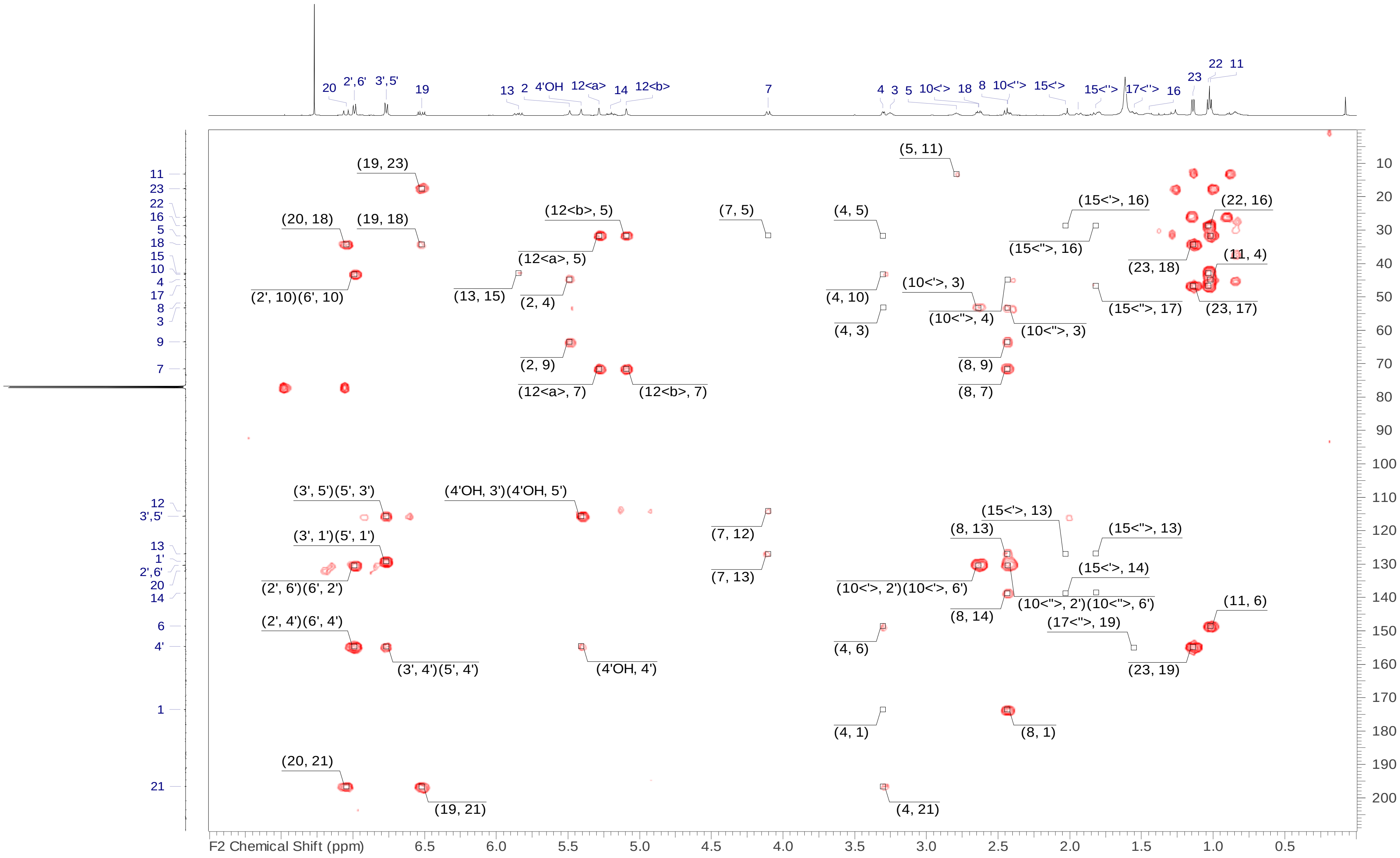

Figure S41. HMBC NMR spectrum (500 MHz, $CDCl_3$) of phenochalasin C (**19**).

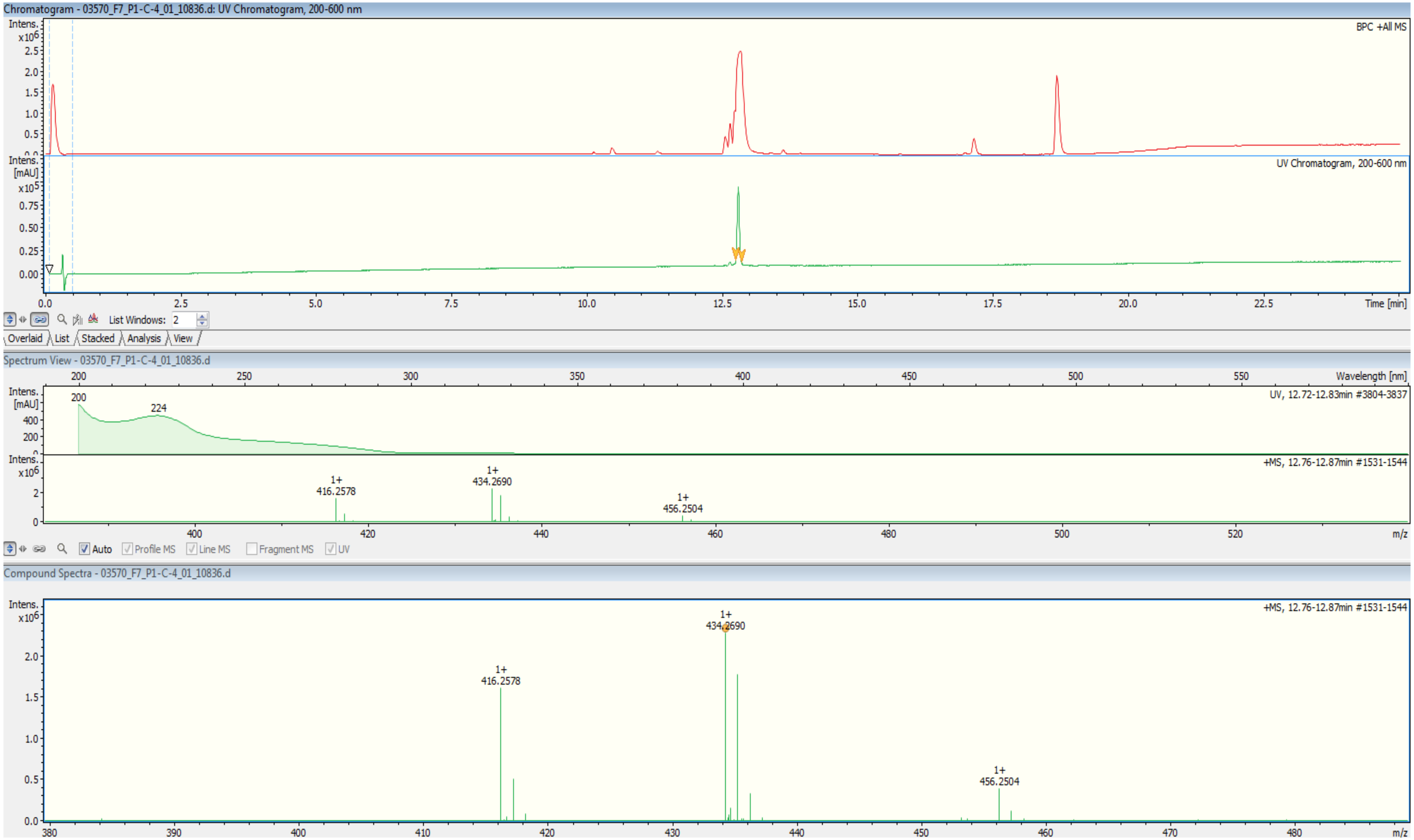

Figure S42. HPLC-HRESIMS data of phenochalasin D (**20**).

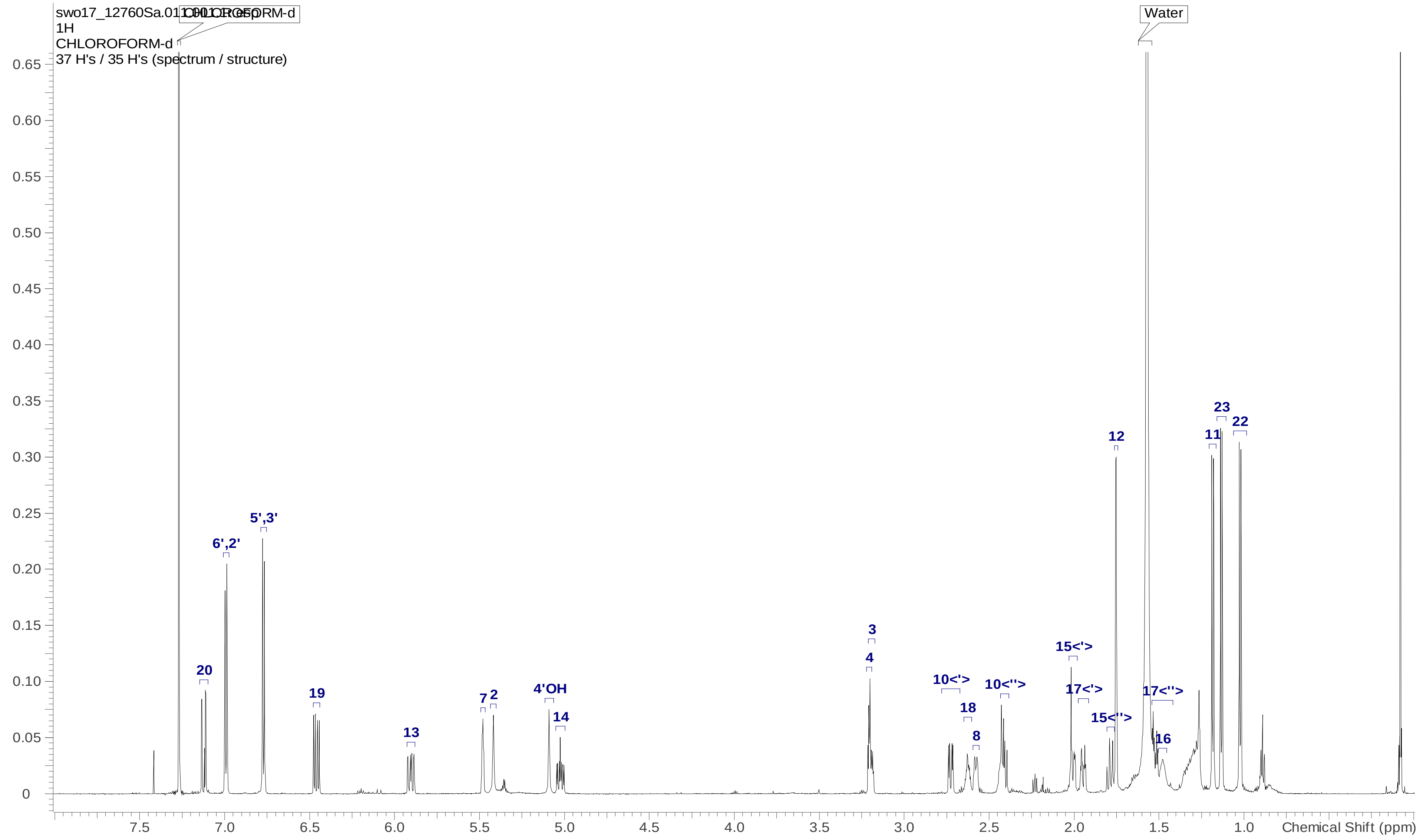

Figure S43. ^{1}H NMR spectrum (700 MHz, $CDCl_3$) of phenochalasin D (**20**).

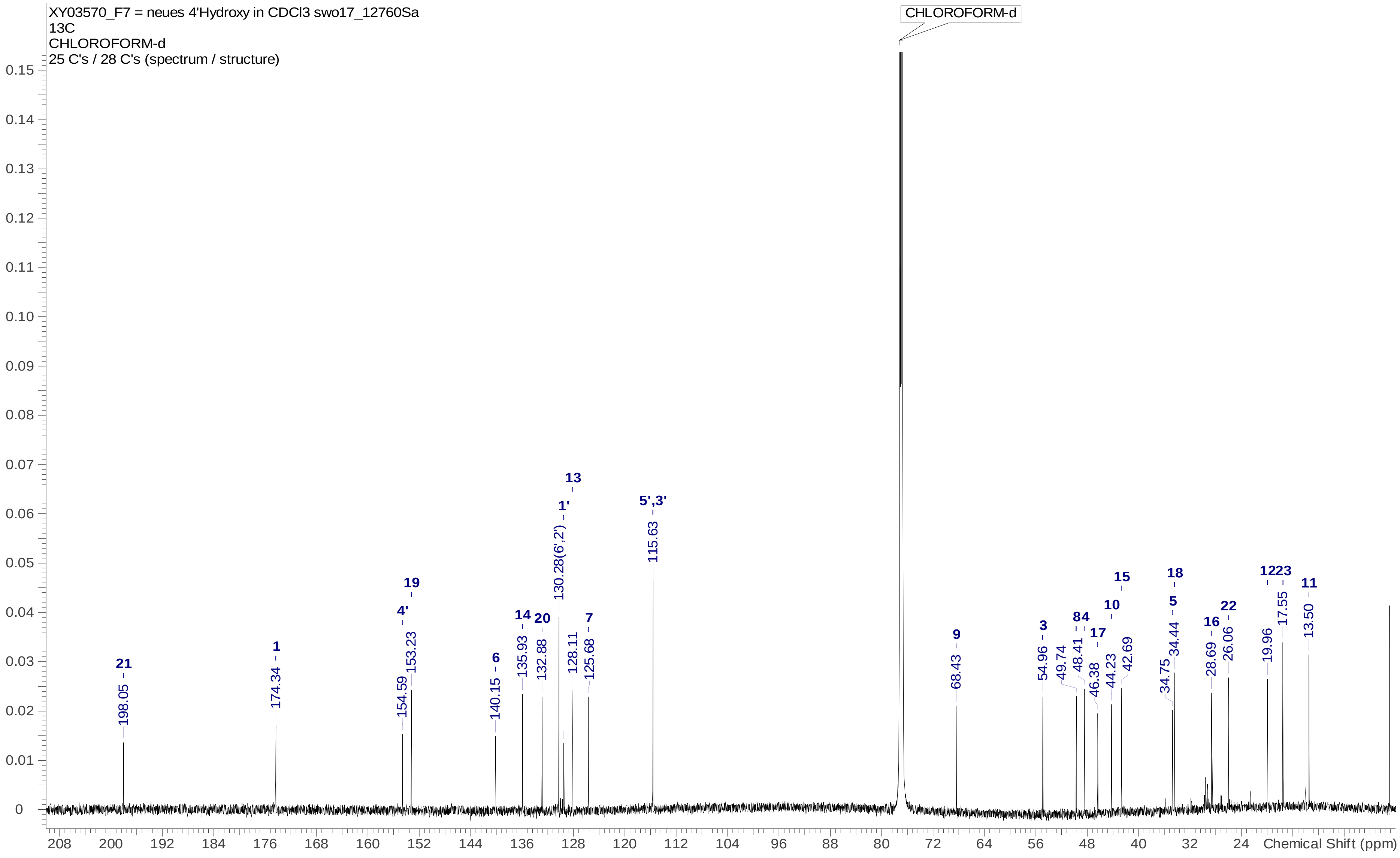

Figure S44. ^{13}C NMR spectrum (175 MHz, $CDCl_3$) of phenochalasin D (**20**).

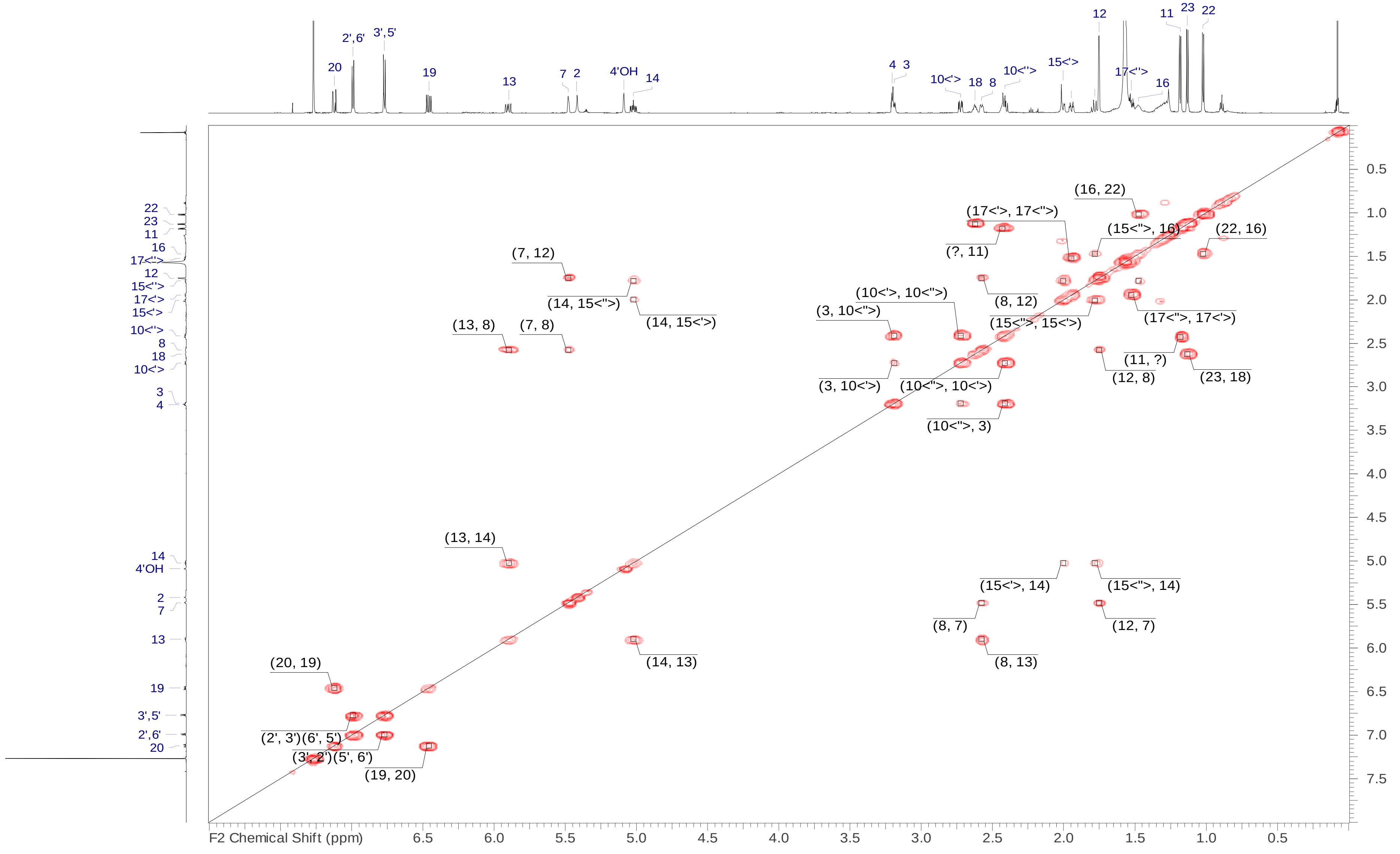

Figure S45. COSY NMR spectrum (700 MHz, $CDCl_3$) of phenochalasin D (**20**).

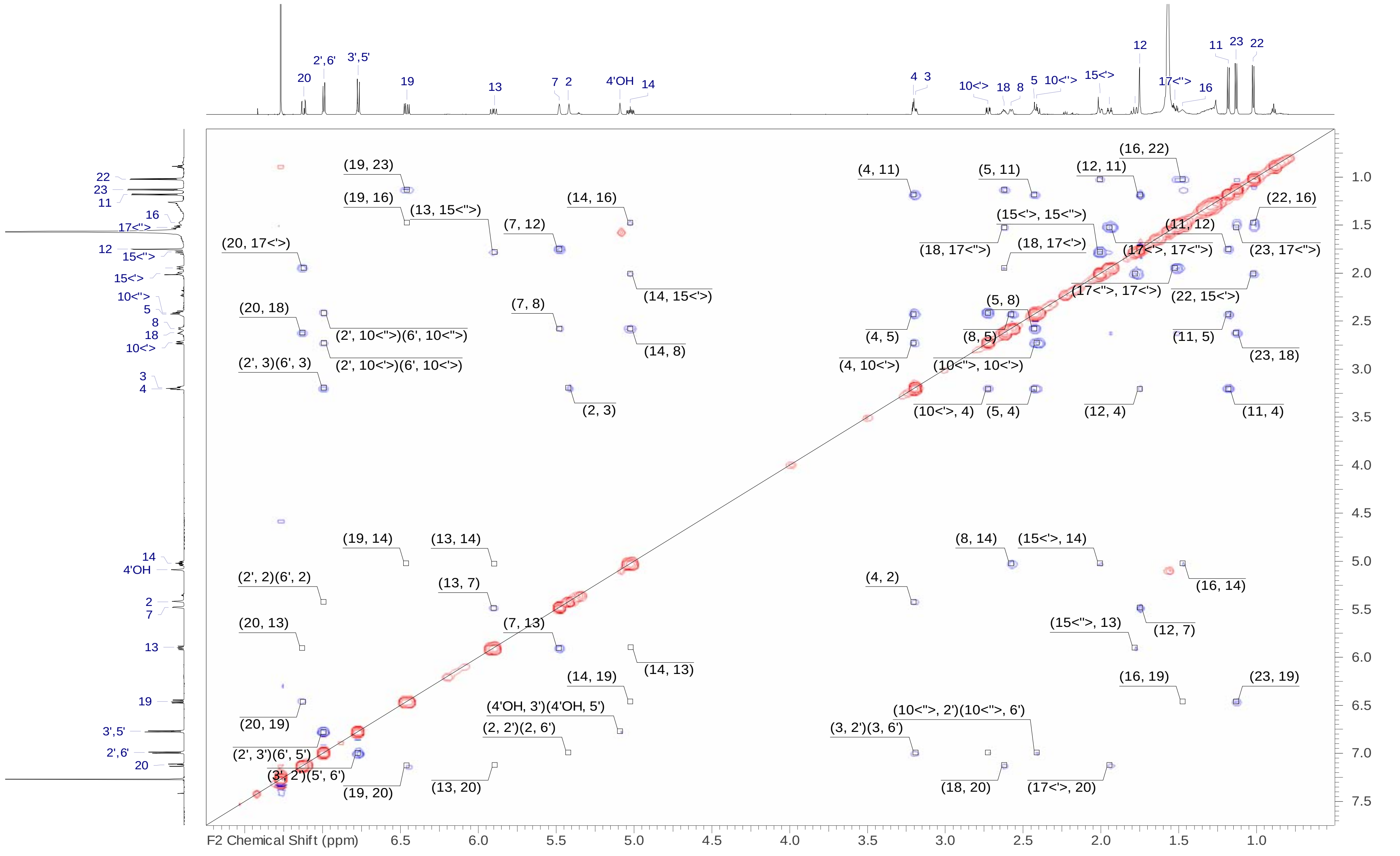

Figure S46. ROESY NMR spectrum (700 MHz, $CDCl_3$) of phenochalasin D (**20**).

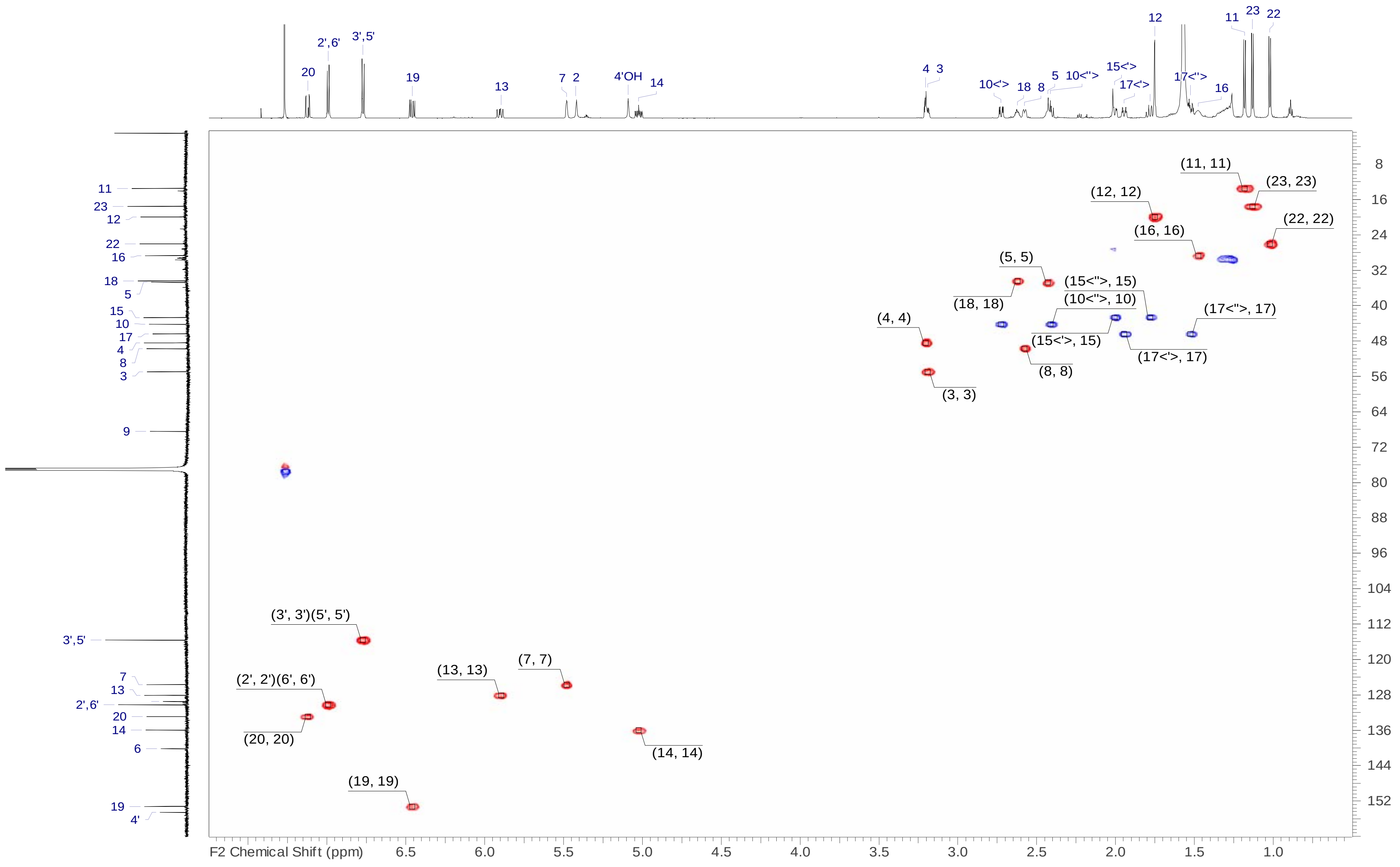

Figure S47. HSQC NMR spectrum (700 MHz, $CDCl_3$) of phenochalasin D (**20**).

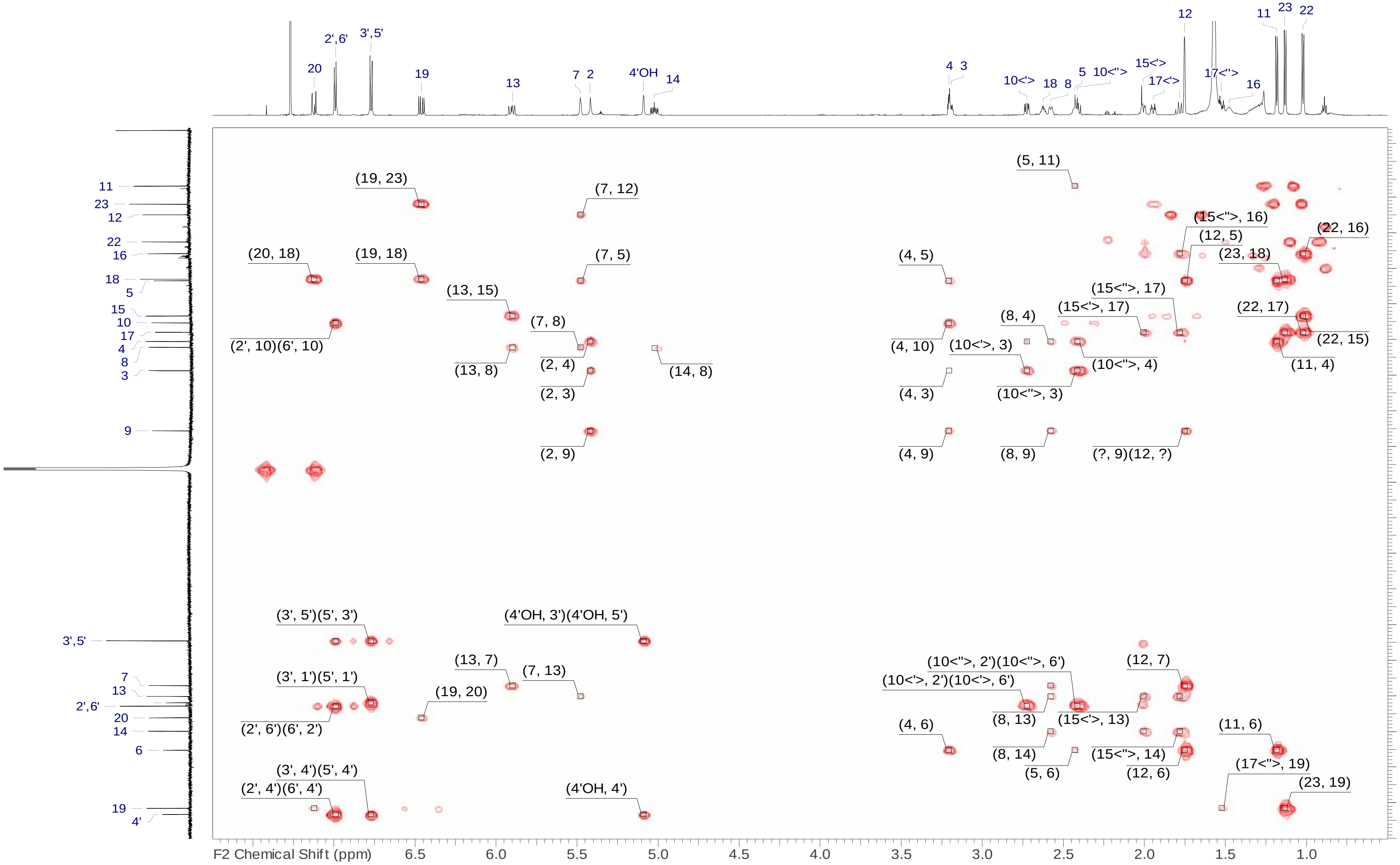

Figure S48. HMBC NMR spectrum (700 MHz, $CDCl_3$) of phenochalasin D (**20**).

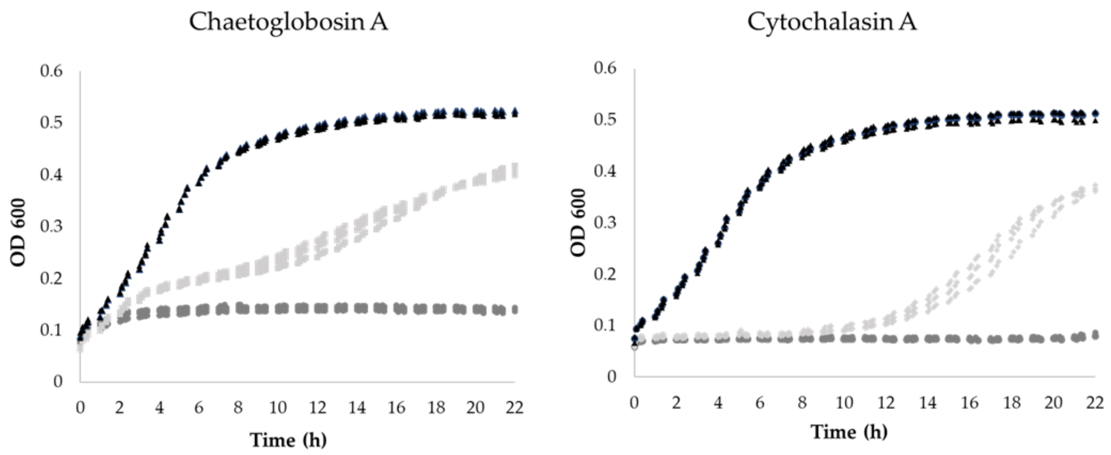

S49. Effects of cytochalasans on the growth of *Staphylococcus aureus*. In chaetoglobosin A: Black triangles are the negative control (methanol), light grayish squares represented the compound at 128 µg ml^{-1} and dark grayish balls represented the compound at 256 µg ml^{-1}, the MIC was 256 µg mL^{-1} (bacteriostatic effect). In cytochalasin A: Black triangles are the negative control (methanol), light grayish diamonds represented 16 µg ml^{-1} and dark grayish balls represented 32 µg ml^{-1} of the compound, the MIC of cytochalasin A was 32 µg ml^{-1} (bacteriostatic effect).

S50. Pictures of the inhibition of biofilm formation from some cytochalasans:

1) (+++) 70-91% of inhibition

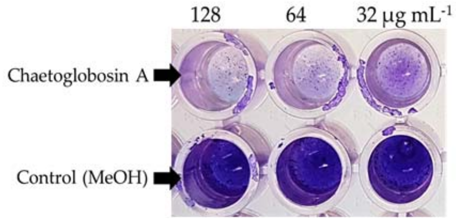

2) (++) 40-70%

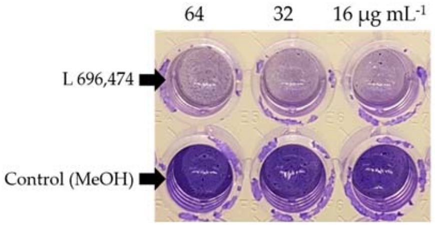

3) (+) 20-40% of biofilm inhibition.

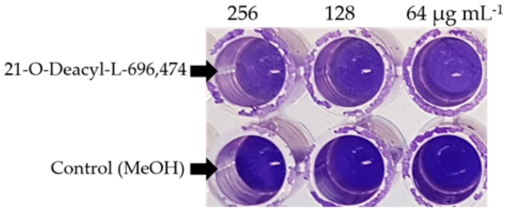

S51. On the table: (+++) 70-91% of inhibition, (++) 40-60%, (+) 20-40% of biofilm inhibition.

2.6 Bioassays

To analyze the minimal inhibitory concentration (MIC) of the cytochalasans, a pre-inoculum of *S. aureus* was cultivated in LB for 24 h, few identical colonies were dispersed in LB medium, diluted with fresh medium to reach the turbidity of 0.5 McFarland and then 150 µl/well were transferred to honeycomb multiwell plates, containing serial dilutions of the cytochalasans (256 to 3 µg mL^{-1}) dissolved in methanol. The microtiter plates were incubated at 37°C in a Bioscreen-C automated growth curve analysis system (Oy Growth Curves AB Ltd, Helsinki, Finland) with linear shaking. During 24 h, the machine measured each 15 min an OD_{600} of bacterial growth per well [7]. To evaluate bactericidic or bacteriostatic effects, aliquots from different concentrations in the wells were inoculated after OD measurements in LB agar for bacterial assays for 24 h. LB medium and methanol were used as negative and tetracycline (100 µg mL^{-1}) as positive controls. Experiments were made in triplicate.

For inhibition of biofilm formation, a pre-inoculum of *S. aureus* grown in CASO with 4% of glucose was adjusted with fresh medium to reach the turbidity of 0.5 McFarland and 150 µl/well were transferred to 96-well tissue microtiter plates (TPP, Switzerland), containing serial dilutions of the cytochalasans (256 to 3 µg mL^{-1}) dissolved in methanol. Plates were covered with a sterile adhesive porous paper (Kisker Biotech GmbH, Steinfurt, Germany). After 20 h, the biofilms in the microtiter plates were indirectly measured by staining with crystal violet. To achieve this the cells were removed after incubation by turning the plate over and shaking out the liquid. Afterwards the plates were gently washed twice with water to remove unattached cells and media components. This was folled by the addition of 125 µL of a 0.1% solution of crystal violet in water to each well and left at room temperature for 10-15 min. Then the plate was rinsed 3-4 times with water followed by drying overnight. For quantification of the biofilms 125 µL of 30% acetic acid in water was added to each well of the microtiter plate to solubilize the dye. After 10-15 min incubation 125 µL of the solubilized crystal violet was transferred to a new flat bottomed microtiter dish. The absorbance was quantified in a plate reader at 550 nm using 30% acetic acid in water as the blank following a published protocol [20]. To quantify the inhibition of the biofilm the formula: [(Control-Blank)-(Sample-Blank)] (%) was used, where: control is the absorbance (550 nm) of the wells containing the negative control (methanol), blank is the absorbance of the wells without the biofilms and the sample is the absorbance of the wells under different concentrations of the cytochalasans. All experiments were performed in triplicates with two repetitions.
